# Spatiotemporal Dynamics of Central Nervous System Diseases: Advancing Translational Neuropathology via Single‐Cell and Spatial Multiomics

**DOI:** 10.1002/mco2.70328

**Published:** 2025-08-19

**Authors:** Mingkai Xia, Quan Liu, Wenli Zhang, Jinwen Ge, Zhigang Mei

**Affiliations:** ^1^ Key Laboratory of Hunan Province for Integrated Traditional Chinese and Western Medicine on Prevention and Treatment of Cardio‐Cerebral Diseases College of Integrated Traditional Chinese Medicine and Western Medicine Hunan University of Chinese Medicine Changsha Hunan China; ^2^ School of Pharmacy Hunan University of Chinese Medicine Changsha Hunan China; ^3^ Hunan Academy of Traditional Chinese Medicine Changsha Hunan China; ^4^ Academy of Chinese Medical Sciences Hunan University of Chinese Medicine Changsha Hunan China

**Keywords:** central nervous system disease, multiomics, molecular mechanisms, precise treatment, single‐cell RNA sequencing, spatial transcriptomics

## Abstract

Central nervous system (CNS) diseases, a leading cause of global disability and mortality, encompass a wide range of brain disorders such as stroke, Alzheimer's disease, Parkinson's disease, and so on. These diseases are characterized by dynamic cellular heterogeneity and disrupted intercellular crosstalk, yet their molecular drivers remain incompletely resolved. Single‐cell RNA sequencing (scRNA‐seq) dissects transcriptional diversity at cellular resolution, while spatial transcriptomics (ST) maps niche‐specific interactions within tissue architecture—complementary approaches that have revealed disease‐associated subpopulations, neural–glial communication, and microenvironmental remodeling. However, standalone omics layers inadequately capture the genetic, epigenetic, and functional cascades underlying CNS pathologies. Here, we highlight the transformative potential of integrating scRNA‐seq and ST with multiomic profiling to delineate spatially orchestrated molecular networks. Such multiomic convergence enables systematic deconstruction of molecular mechanisms and intercellular communication across disease progression. By correlating these signatures with clinical phenotypes, this strategy accelerates biomarker discovery, patient stratification, and therapeutic target identification. We further discuss challenges in data harmonization, subcellular spatial resolution, and computational scalability that must be addressed to realize personalized CNS medicine. This synthesis advocates for interdisciplinary frameworks to translate multiomic insights into mechanistically grounded diagnostics and therapies, ultimately bridging the gap between molecular discovery and precision clinical intervention.

## Graphical abstract

Central nervous system (CNS) diseases are driven by spatiotemporally dynamic molecular networks. Single‐cell and spatial multiomics technologies dissect cellular heterogeneity, microenvironmental remodeling, and intercellular crosstalk. Integration of transcriptomic, epigenomic, proteomic, and metabolomic layers reveals disease‐associated regulatory circuits and accelerates precision diagnostics and therapies.

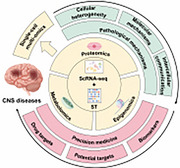

## 1 Introduction

Central nervous system (CNS) disorders, including Alzheimer's disease (AD), Parkinson's disease (PD), and stroke, collectively represent a leading global health burden, driven by complex cellular and molecular interplay that remains poorly resolved. Traditional bulk omics approaches obscure critical cell‐type‐specific dynamics [1], while emerging single‐cell RNA sequencing (scRNA‐seq) and spatial transcriptomics (ST) have begun dissecting CNS pathologies at unprecedented resolution [2, 3, 4]. ScRNA‐seq delineates transcriptional heterogeneity across neuronal, glial, and immune subpopulations in neurodegeneration [5, 6], whereas ST preserves spatial context to map disease‐associated niche interactions [7, 8]. Despite these advances, standalone transcriptomic layers fail to capture the multilayered genetic, epigenetic, and metabolic cascades underlying cellular dysregulation [9, 10]. For instance, posttranscriptional modifications and metabolic reprogramming in AD astrocytes are inadequately profiled by RNA‐centric methods alone [11].

Integrating scRNA‐seq and ST with epigenomic, proteomic, and metabolomic datasets offers a transformative lens to decode spatially orchestrated molecular networks [12, 13]. Such multiomic convergence has revealed regulatory hierarchies in neuroinflammation [14], synaptic metabolic coupling [15], and microenvironmental crosstalk in ischemic stroke (IS) [16]. For example, combined ATAC‐seq and scRNA‐seq identified enhancer landscapes driving microglial activation in AD [17], which underscore the potential to deconstruct CNS diseases across molecular layers, linking genomic variants to functional pathways and clinical phenotypes.

However, critical challenges persist. Technical disparities in multiomic data resolution, spatial granularity, and computational integration limit mechanistic insights. Current ST platforms lack subcellular resolution to localize RNA–protein interactions, while crossmodal data harmonization requires advanced machine learning frameworks [18]. Addressing these gaps is pivotal for translating multiomic signatures into biomarkers and therapies, such as circuit‐specific neuromodulation [19].

This review synthesizes advances in CNS multiomics, compared with the existing researches. Our review not only focuses on the mainstream CNS diseases, but also places significant emphasis on conditions where single‐cell and spatial analyses remain scarce, including epilepsy, autism spectrum disorder (ASD), and amyotrophic lateral sclerosis (ALS). We move beyond merely summarizing applications of individual technologies to pioneer a discussion on the integration of scRNA‐seq and ST data with other multiomics datasets. This deeply integrated analytical strategy surpasses the limitations of single‐technology approaches, aiming to elucidate a more comprehensive understanding of the pathological process in CNS diseases. Furthermore, we specifically highlight findings possessing clinical translational potential, emphasizing their role in precise diagnosis, individual treatment, and drug development. We critically evaluate technical frontiers, clinical translation potential, and interdisciplinary strategies to bridge molecular discovery with therapeutic innovation.

## 2 Single‐Cell RNA Sequencing

**FIGURE 1:**
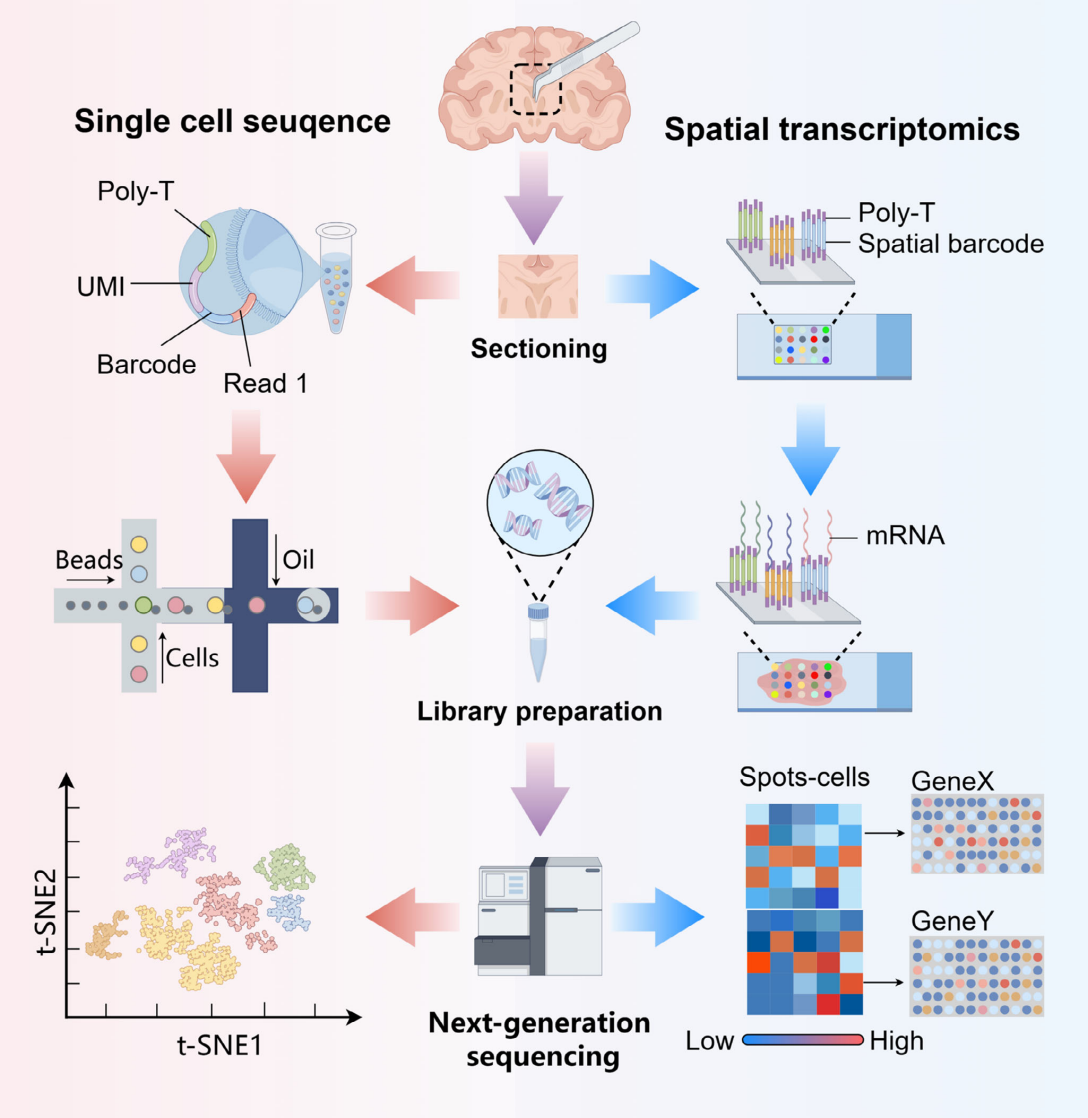
Workflow of single‐cell RNA sequencing and spatial transcriptomics technologies. In single‐cell RNA sequencing, first single‐cell or ‐nuclei suspensions are generated from the tissue, then suspensions are encapsulated in nanoliter droplets containing barcode beads using a microfluidic device. Post cell lysis, capture of polyadenylated RNA, reverse transcription, introduction of unique molecular identifiers (UMIs) and cell barcodes, and cDNA amplification and fragmentation, next‐generation sequencing is performed on cDNA library. After sequencing, a dimensionality reduction algorithm can be used to visualize high‐dimensional single‐cell RNA sequencing data to reveal cell clusters. In spatial transcriptomics, tissues are placed on mRNA capture slides for sectioning, fixing, and permeabilization to release RNA. The poly‐A tail of mRNA binds to the oligonucleotide (dT) terminal segment on the capture DNA probes, which also include locational barcodes. After library preparation and sequencing, computational analysis involves retrieving positional barcodes and tissue coordinates to reconstruct the relationship between transcripts and their locations. This figure was created using Figdraw.

ScRNA‐seq enables high‐resolution transcriptional profiling of individual cells, overcoming the limitations of bulk sequencing in resolving cellular heterogeneity. The advent of scRNA‐seq enables transcriptional profiling at single‐cell resolution, overcoming the limitations of bulk sequencing in resolving cellular heterogeneity. Recent years have witnessed rapid evolution in single‐cell sequencing technologies, marked by significant advancements in throughput, efficiency, gene detection sensitivity, and cost effectiveness. Here, we introduce the fundamental workflow of scRNA‐seq and highlight key technological improvements (Figure 1).

### 2.1 Cell Isolation and Barcoding

Effective single‐cell suspension preparation with preserved viability is critical. Early low‐throughput methods (mechanical micromanipulation, laser capture microdissection) have been superseded by high‐throughput platforms, including microfluidics (e.g., 10x Genomics Chromium) and droplet‐based systems. These technologies coencapsulate cells with barcoded gel beads in emulsions, enabling simultaneous processing of thousands of cells while preserving transcriptomic integrity [20, 21].

### 2.2 cDNA Amplification and Library Construction

Given the picogram‐scale RNA yield per cell, cDNA amplification is essential. Common strategies include polymerase chain reaction (PCR) and in vitro transcription (IVT) [22]. PCR‐based template‐switching oligonucleotide methods dominate due to their efficiency, despite inherent amplification biases (e.g., 3′ coverage skews in IVT) [22]. Unique molecular identifiers are integrated to mitigate PCR duplication artifacts, ensuring quantitative accuracy [23].

### 2.3 Data Analysis and Functional Insights

Post sequencing, raw data undergo quality control, normalization, and dimensionality reduction (PCA, UMAP/t‐SNE) prior to clustering. Differential gene expression analysis identifies subpopulation‐specific markers, enabling pseudotime trajectory inference to map cellular dynamics [24]. Advanced tools like SCENIC reconstruct gene regulatory networks by linking transcription factor activity to expression patterns [25]. Functional enrichment analyses, such as Gene Ontology (GO) and Kyoto Encyclopedia of Genes and Genomes, further contextualize subcluster phenotypes within pathways such as immune activation or cell cycle regulation. This pipeline's resolution has redefined our understanding of cellular diversity, offering mechanistic insights into disease progression and therapeutic targets.

### 2.4 Comparison and Progress of Common scRNA‐seq Technologies

Currently, microdroplet‐based scRNA‐seq, exemplified by the 10x Chromium platform (10X), represents the most widely adopted approach. The 10X protocol enables high cell capture efficiency at relatively low cost while minimizing manual handling, thereby facilitating the generation of large‐scale single‐cell datasets [26]. Consequently, 10X is particularly advantageous for identifying rare cell subtypes and delineating cellular communication networks in complex systems, which is critical for elucidating unique pathological mechanisms in CNS diseases [27]. However, this method exhibits pronounced 3′‐end bias in mRNA reads, potentially reducing gene detection sensitivity [28]. In contrast, Smart‐seq2 generates full‐length transcript libraries with more uniform coverage across mRNA transcripts, demonstrating superior sensitivity for gene detection [28, 29]. This approach is therefore better suited for profiling low‐abundance transcripts and investigating cell–cell interactions [28], making it widely applicable in CNS disease research focused on gene expression dynamics and transcriptional states in specific cell populations [30, 31]. However, compared with droplet‐based methods like 10X, Smart‐seq2 still has lower throughput and high cost, limiting its application in large‐scale studies.

Recent methodological innovations have significantly expanded the resolution of single‐cell transcriptomics. Mahat et al. [32] developed single‐cell global run‐on sequencing (scGRO‐seq), a breakthrough technology that overcomes critical limitations of conventional poly(A)‐dependent scRNA‐seq. By leveraging copper‐catalyzed azide‐alkyne cycloaddition (CuAAC or click chemistry), scGRO‐seq directly captures nascent RNA transcripts genome‐wide within individual cells, enabling unprecedented temporal and spatial analysis of transcriptional dynamics. Notably, the emergence of UDA‐seq, a universal workflow incorporating a postindexing step, has enhanced throughput by systematically adapting existing droplet‐based multimodal methods [33]. UDA‐seq achieves ultra‐high throughput and data quality comparable to 10X while significantly expanding sample sizes and cellular coverage depth. This advancement enables robust identification of rare cell phenotypes, positioning it as an ideal platform for large‐scale, multisample scRNA‐seq studies, such as constructing comprehensive cellular atlases of the CNS.

## 3 Technical Advances in ST

This emerging technology was first presented in 2016 [4]. Recently, the mainstream ST technology can be divided into the imaging‐based methods and the sequencing‐based methods (Table 1).

**TABLE 1:** List of spatial transcriptomics technologies.

Method	Name	Sample types	Spatial resolution	Advantages	Limitations	Number of publication^a^	Representative studies	References
Sequencing‐based	Visium	FF	Multicellular	Whole mRNA analysis	Barcoded regions contain multiple cells	255	[34, 35, 36]	[4]
	Slide‐seq	FF	Cellular	High spatial resolution	Not suitable for heterogeneous samples, and time‐consuming	36	[37, 38]	[39]
	HDST	FF	Subcellular	High spatial resolution	Low detection efficiency	13	[40]	[40]
	DBiT‐seq	FF	Cellular	Adjustable spatial resolution	Does not resolve single cell	6	[41]	[42]
	Stereo‐seq	FF	Subcellular	High spatial resolution and capture efficiency	High cost	79	[43, 44]	[45]
	Ex‐ST	FF	Subcellular	High spatial resolution and capture efficiency	Time‐consuming	1	[46]	[46]
	Open‐ST	FF	Subcellular	High spatial resolution, cost‐effectivity, and 3D scalability	Tissue type limitation	2	[47, 48]	[47]
	sci‐Space	FF	Cellular	Retrieve single‐cell level transcriptome	Suffer from RNA integrity issues	2	[49, 50]	[49]
	XYZeq	FF	Cellular	Retrieve single‐cell level transcriptome	Suffer from RNA integrity issues	1	[51]	[51]
	Spotiphy	FF	Cellular	Whole genome coverage and high spatial resolution	Lack of ability to explore unknown cell types and high cost	1	[52]	[52]
ISH‐based	smFISH	FFPE or FF	Subcellular	High sensitivity to detect individual transcripts	Low throughput	31	[53]	[54]
	seqFISH	FF	Subcellular	Multiplexing	High detection error and high cost	37	[55]	[56]
	MERFISH	FF	Subcellular	Multiplexing, and low detection error	High cost	101	[57, 58]	[59]
	TRISCO	FFPE or FF	Cellular	Whole brain spatial 3D imaging	High cost and time‐consuming	1	[60]	[60]
ISS‐based	ISS	FFPE or FF	Subcellular	In situ sequence the individual RNA	Low throughput	20	[61]	[62]
	FISSEQ	FFPE or FF	Subcellular	Unbiased examination of the whole transcriptome distribution	High proportion of signals from rRNA	5	[63]	[64]
	STARmap	FF	Subcellular	Low error rate and the ability to perform 3D spatial analysis of the samples	Low throughput	21	[65]	[66]
	BaristaSeq	Cultured cells	Subcellular	Improve the signal‐to‐noise ratio and read length	Low throughput	2	[67, 68]	[67]
	BARseq	Cultured cells	Cellular	Construct the connection map of neurons with low error and high throughput	Low throughput	7	[69]	[70]
	BARseq2	Cultured cells	Cellular	Ability to correlate the expression of many genes to projections to many targets in the same neurons	Relatively low spatial resolution	1	[71]	[71]
	MiP‐seq	FFPE or FF cultured cells	Subcellular	In situ multiomics sequencing, cost‐effectivity, low error rate, compatibility with other imaging technologies and single‐base resolution	Dependence on the number of probes and restricted ability to directly detect RNA N6‐methyladenosine modification	1	[72]	[72]

Abbreviations: BaristaSeq, barcode in situ targeted sequencing; BARseq, barcoded anatomy resolved by sequencing; DBiT‐seq, deterministic barcoding in tissue for spatial omics sequencing; Ex‐ST, Expansion spatial transcriptomics; FF, fresh‐frozen; FFPE, formalin fixation and paraffin embedding; FISH, fluorescence in situ hybridization; FISSEQ, in fluorescence in situ sequencing; HDST, high‐definition spatial transcriptomics; ISH, in situ hybridization; ISS, in situ sequencing; MERFISH, multiplexed error‐robust FISH; NGS, next‐generation sequencing; seqFISH, sequential FISH; smFISH, single molecular FISH; STARmap, spatially resolved transcript amplicon readout mapping; TRISCO, Tris buffer‐mediated retention of in situ hybridization chain reaction signal in cleared organs.

^a^ All data were systematically retrieved from PubMed, employing the following search query syntax: (method name) AND (spatial transcriptomics) NOT (review[publication type] OR review[Title/Abstract]). The literature search was conducted up to July 23, 2025, ensuring inclusion of the most recent and relevant studies while excluding review articles.

### 3.1 Sequencing‐Based Methods

Different from imaging‐based methods, sequencing‐based methods provide low resolution, but its advantages of large coverage and no bias make it still one of the mainstream methods of ST (Figure 1).

Slide‐seq was developed in 2019 [39], which uses microparticles called “beads” to measure the expression of the whole genome at a resolution of 10 µm. Each bead is coated on the slide and modified with 500 spatial barcode reverse transcription primers. After the capture of mRNA, the barcode on each bead is determined through sequencing by oligonucleotide ligation and detection technology to generate the spatial information of barcode. Shortly after Slide‐seq, high‐definition ST (HDST) was developed [40], with beads loaded in a large number of small holes on the slide. Compared with Slide‐seq, HDST uses smaller beads to construct a higher density array. Deterministic barcoding in tissue for spatial omics sequencing (DBiT‐seq) does not depend on the spatial barcode oligonucleotide array fixed on slide [42]. In DBiT‐seq, arrays are “printed” onto tissues through the microfluidic channel. The microfluidic channel modifies 50 rows of barcodes for tissue sections by negative pressure injection, and followed by 50 rows of barcodes in the vertical direction in the same way. Each point can be identified with a unique pair combination. Stereo‐seq combined DNA nanoball pattern arrays with in situ RNA capture techniques [45]. Compared with other ST technologies, Stereo‐seq had a large field of view, nanoscale resolution, high sensitivity, and uniform capture rate. Expansion ST (Ex‐ST) embeds tissue sections in a polyacrylamide gel to anchor RNA, which overcomes the limitations of array density and achieves higher spatial resolution [46]. Open‐ST transforms Illumina flow cytometers into ST capture areas by utilizing patterned flow cytometry technology to generate densely barcoded regions [47], enabling the capture of polyadenylated RNA from tissue sections. This approach achieves precise identification of distinct cell types and their molecular characteristics at a higher spatial resolution. Furthermore, it allows for the computational integration of two‐dimensional data into three‐dimensional (3D) virtual tissue blocks, which are applicable to a wide range of tissue types.

However, these methods are still inadequate to reflect the actual single‐cell level transcripts in space because cells often span multiple capture regions; hence, transcripts inferred from multiple small pixels have inherent limitations in their correspondence to true single‐cell level data. To overcome this limitation, sci‐Space and XYZeq have integrated the scRNA‐seq with ST technology [49, 51], also using a spatial barcode array, to label cells rather than capturing mRNA, and after assigning spatial barcodes to each cell, scRNA‐seq is used on cells with spatial information. Spotiphy is a toolkit designed to address the trade‐offs in gene coverage [52], capable of transforming sequencing‐based ST data into whole‐transcriptome images with pseudo‐single‐cell resolution. The highlight of this protocol lies in its integration of the most informative genes for each cell type and the nuclear positions into a probabilistic model, which takes into account the contribution of each cell type to gene expression. This enables the deconvolution and decomposition of ST data, generating cell type proportions and inferred scRNA data (iscRNA data). Subsequently, Gaussian processes are employed to interpolate the cell type proportions and iscRNA data in noncaptured regions, producing whole‐slide pseudo‐single‐cell resolution whole‐transcriptome images.

### 3.2 Imaging‐Based Methods

Two main technologies are mature, including in situ sequencing (ISS)‐based methods and in situ hybridization (ISH)‐based ST methods [73, 74, 75].

#### 3.2.1 ISH‐Based ST Methods

The current mainstream ISH method is to combine fluorescence ISH (FISH) with scRNA‐seq, and nucleotide probes with different fluorescent stains with the target RNA, using one color for each hybridization. Eight rounds of hybridization are sufficient to resolve all genes in the human genome. Single‐molecule FISH (smFISH) is the first ST technique started in FISH [54], which used digital imaging microscope to in situ detect oligonucleic acid probes modified with fluorescent in individual cells that hybridize to specific mRNA, thus, can determine the abundance of intracellular transcripts and achieve spatial localization [76]. However, due to the spectral limitation, only a few targets RNA can be detected at a time. Compared with smFISH, the advantage of sequential FISH (seqFISH) is in each hybridization [56], FISH probe is labeled with a single color to each transcript, after hybridization, imaging and probe stripping, the number of F^N^ species transcripts have been barcoded, which can cover the entire transcriptome (F: number of fluorophores, N: number of hybridization rounds). However, due to the high density of mRNA in cells, optical crowding occurs during fluorescence hybridization, which is not conducive to the analysis of mRNA; thus, seqFISH was further developed to seqFISH+ [77], using more than 60 kinds of pseudocolor expanded the barcode base color and divided them into three fluorescent channels; barcodes were generated only within each fluorescent channel, to ensure the building of super resolution image.

Despite seqFISH is able to detect RNA targets at levels of up to 10, 000 transcripts per cell, the researchers have to face questions during the experiment like high detection errors, time consuming, and high costs. Multiplexed error‐robust FISH (MERFISH) was developed in 2015 [59]; during this method, RNA is hybridized with 192 coding probes consisting of a targeting sequence with two readout fluorescent probes. In each round of hybridization, each gene was assigned a binary bit coding; the number of bits correspond to the number of hybridization rounds, then align them with the designed targeting barcode to obtain the specific information of the RNA. Tris buffer‐mediated retention of ISH chain reaction signal in cleared organs (TRISCO) was developed recently [60]; this emerging protocol enhances the RNA probe signals in deep brain regions by adjusting the staining temperature, thereby achieving 3D RNA imaging across the entire brain.

#### 3.2.2 ISS‐Based Methods

The basic principle of ISS technology is as follows: first, mRNA in specific tissues and cells is reverse transcribed into cDNA, the lock probe is specifically combined with the target cDNA sequence, and the probe is loop by DNA polymerase. Through rolling‐circle amplification (RCA), the nucleic acid information of the target region is amplified. Finally, the RCA products were sequenced to achieve decoding of transcriptome information and mapping of spatial information [62]. Fluorescence ISS (FISSEQ) enables nontargeting RNA capture [64], using random primers to generate cDNA and crosslink cDNA to the cellular environment, to loop and link cDNA by single‐stranded DNA cyclase. Spatially resolved transcript amplicon readout mapping (STARmap) improved ISS by applying CLARITY‐based hydrogel‐tissue chemistry, SNAIL probes, and sequencing with error‐reduction by dynamic annealing and ligation [66]. Furthermore, the method also retains the directionality of cells and, therefore, allows for 3D spatial analysis of tissue samples [78]. Barcode in situ targeted sequencing (BaristaSeq) was an improved version of gap padlock probe‐based method and relies on padlocks [67]. During this approach, there is a gap between the two arms of the padlock probe, which DNA polymerase is used to fill before the circularization of padlock probe. This procedure copies a portion of the cDNA into the RCA template, replicated barcodes were then sequenced in situ. Since then, Chen et al. [70, 71] have continued to developed barcoded anatomy resolved by sequencing (BARseq) and BARseq2.

## 4 Integrative Innovation of Single‐Cell and Spatial Multiomics Technologies

The integration of single‐cell and spatial multiomics with epigenomic, proteomic, and metabolomic datasets is revolutionizing our understanding of CNS diseases. Below, we outline key advancements and validated applications supported by published studies.

### 4.1 Epigenomic Integration: Decoding Spatial Regulatory Logic

Single‐cell epigenomics, such as single‐cell assay for transposase‐accessible chromatin using sequencing (scATAC‐seq) and single‐cell cleavage under targets and tagmentation [79, 80], identifies cell‐type‐specific chromatin states, while spatial epigenomics (e.g., spatial‐CUT&Tag and Epigenomic MERFISH) [81, 82] resolves regulatory elements within tissue niches. Combined, these approaches reveal disease‐associated epigenetic dysregulation. For example, by integrating scATAC‐seq data with published snRNA‐seq datasets, researchers constructed gene regulatory networks in adult mouse brain regions, focusing on the transcription factor *Jun*, to investigate its role in spinal cord regeneration [83].

### 4.2 Proteomic Integration: Mapping Functional Crosstalk

Single‐cell proteomics quantifies surface protein heterogeneity, including cellular indexing of transcriptomes and epitopes by sequencing (CITE‐seq) [84] and RNA expression and protein sequencing [85], whereas spatial proteomics localizes intercellular signaling hubs, such as multiplexed ion beam imaging by time‐of‐flight [86], cellular detection by expression of multiple proteins (CODEX) [87], 3D imaging of solvent‐cleared organs profiled by mass spectrometry (DISCO–MS) [88], label‐based unlimited multiplexed DNA‐PAINT (SUM‐PAINT) [89]. Their synergy maps ligand–receptor dynamics across niches, for example, to reveal the distribution, function, and other cell–cell interactions between innate immune cells and surrounding cells (endothelial cells and fibroblasts) at the CNS border [90].

### 4.3 Metabolomic Integration: Linking Metabolic States to Spatial Niches

Single‐cell metabolomics currently encompasses two mainstream methodologies: mass spectrometry‐based single‐cell metabolomics approaches, such as matrix‐assisted laser desorption/ionization–mass spectrometry imaging (MALDI–MSI) [91], and flow cytometry‐based single‐cell metabolic profiling approaches, including Met‐Flow [92]. Both approaches enable the capture of cellular metabolic heterogeneity. While spatial metabolomics maps metabolite gradients across tissue architectures, emerging techniques like spatial single nuclear metabolomics display multiscale and multicolor tissue tomography together with the identification and clustering of single nuclei by their in situ metabolic fingerprints [93]. Integrated analysis of scRNA‐seq, Visium, and air flow‐assisted desorption electrospray ionization (AFADESI)–MSI [94] reveals spatial heterogeneity in gene expression and metabolic alterations following traumatic brain injury. For instance, *S1pr5*‐high regions exhibit elevated phosphatidylcholine PC(44:7) levels, while *Septin4*‐high regions correlate with reduced aspartate distribution.

In a result, integrating single‐cell and spatial multiomics with epigenomic, proteomic, and metabolomic data revolutionizes CNS disease understanding. This synergy deciphers spatial regulatory logic, maps functional cell–cell crosstalk, and links metabolic states to tissue niches, revealing related‐CNS disease mechanisms.

## 5 Molecular Mechanisms of CNS Diseases Suggested by Single‐Cell and Spatial Multiomics

With the decreasing cost of next‐generation sequencing and improvement in computational power, microscopy, and imaging, scRNA‐seq and ST have been widely used to meet the demands of researchers. These methods help identify cell populations associated with CNS diseases such as stroke, AD, PD, and glioblastoma (GBM). Combined with multiomic data analysis methods, researchers can understand the pathological characteristics around the damaged area. These include neuroinflammation, mitochondrial dysfunction, oxidative stress, and other repair mechanisms, such as remyelination [95, 96].

### 5.1 Stroke

#### 5.1.1 Ischemic Stroke

**FIGURE 2:**
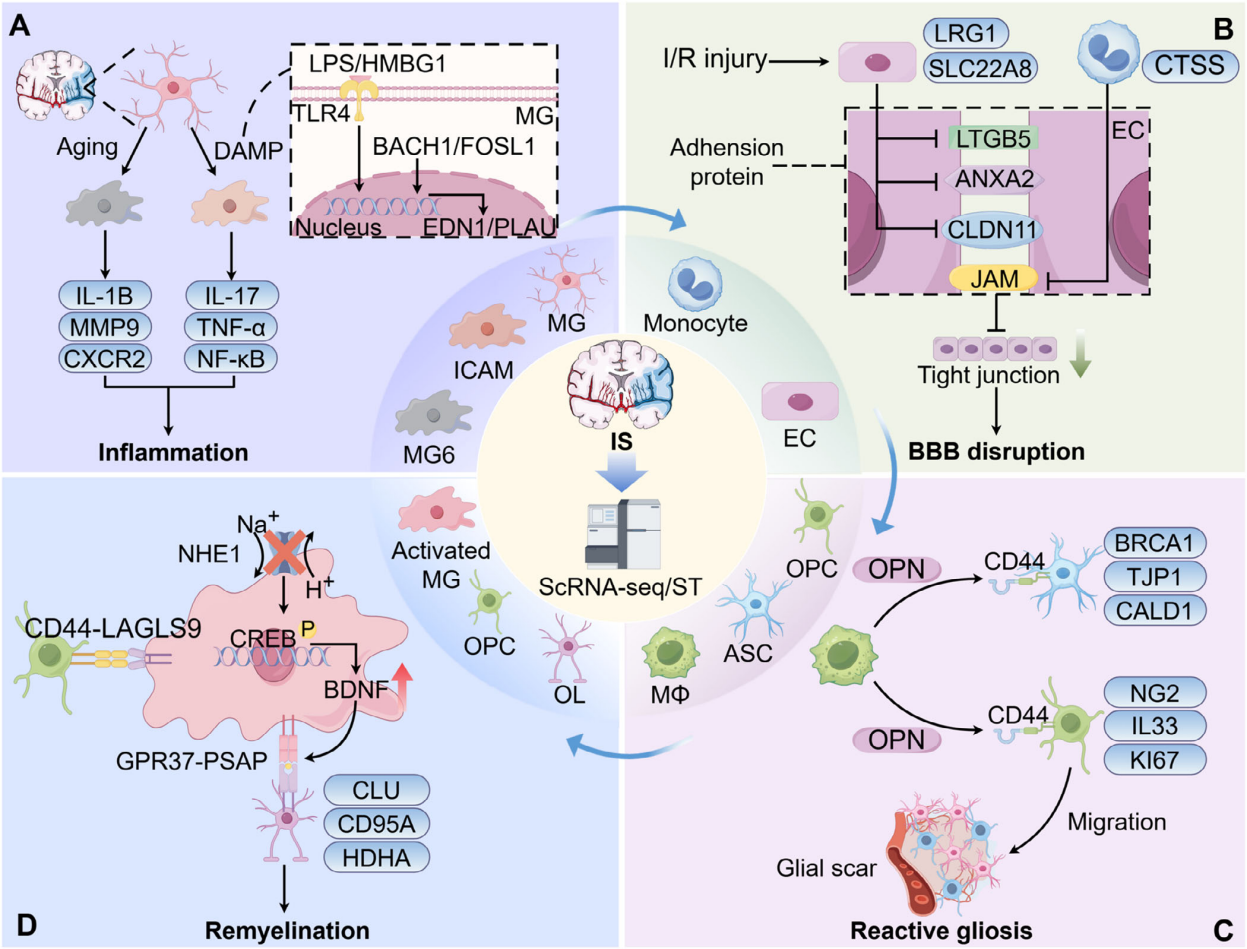
Cellular changes post ischemic stroke revealed by single‐cell and spatial multiomics. (A) At the acute stage of ischemic stroke (IS), microglia (MG) in the ischemic core changes to ischemic core‐associated microglia (ICAM), which has high expression of proinflammatory genes. Additionally, a subset of MG in the aged brain post IS also highly express proinflammatory genes. (B) Ischemia–reperfusion injury post IS activates endothelial cells and monocytes to degrade adhesion proteins, thereby undermining the integrity of the blood–brain barrier. (C) During the subacute stage of IS, myeloid cells induce the migration of proliferating glial cells to the lesional rim by osteopontin–CD44 signaling, promoting the formation of glial scars. (D) During the chronic stage of IS, MG promotes the proliferation of OPC through LAGLS9–CD44 signaling. Na^+^/H^+^ exchanger‐1 (NHE1) in MG regulates intracellular pH to mediate demyelination effect, selective deletion of NHE1 can regulate MG‐oligodendrocyte interaction through CREB1 signaling to promote oligodendrocyte proliferation and remyelination. ANXA2: annexin A2; ASC: astrocyte; BACH1: BTB and CNC homology 1; BBB: blood–brain barrier; BDNF: brain derived neurotrophic factor; BRCA1: breast cancer 1; CALD1: caldesmon 1; CLDN11: claudin 11; CLU: clusterin; CREB: cAMP‐response element binding protein; CTSS: cathepsin S; CXCR2: C‐X‐C motif chemokine receptor 2; DAMP: damage associated molecular pattern; EC: endothelial cell; EDN1: endothelin 1; FOSL1: FOS like antigen 1; GPR37: G protein‐coupled receptor 37; HMBG1: high mobility group box‐1 protein; ICAM: ischemic core‐associated microglia; IL‐17: interleukin‐17; IL‐1B: interleukin‐1β; I/R: ischemia/reperfusion; IS: ischemic stroke; JAM: junctional adhesion molecule; LAGLS9: galectin 9; LPS: lipopolysaccharide; LRG1: leucine rich alpha‐2‐glycoprotein 1; LTGB5: integrin subunit beta 5; MG: microglia; MMP9: metallopeptidase 9; MΦ: macrophage; NF‐κB: nuclear factor kappa‐B; NHE1: Na^+^/H^+^ exchanger‐1; OL: oligodendrocyte; OPC: oligodendrocyte precursor cell; PLAU: plasminogen activator; PSAP: prosaposin; SLC22A8: solute carrier family 22 member 8; TJP1: tight junction protein 1; TLR4: toll‐like receptor 4; TNF‐α: tumor necrosis factor‐alpha. This figure was created using Figdraw.

During the acute phase of IS, intense neuroinflammation is associated with neuronal damage and worse neurological outcomes [97]. Microglia play a crucial role in poststroke immune inflammation [98]. However, the heterogeneity and involved mechanisms of microglia remain unclear. In an experiment combining scRNA‐seq and ST to sequence microglia in the ischemic hemisphere of middle cerebral artery occlusion (MCAO) mice, researchers identified two spatially specific subpopulations as ischemic core‐associated microglia (ICAM) and ischemic penumbra‐associated microglia [99]. Gene set variation analysis indicated that compared with the other clusters, the expression of proinflammatory and inflammatory response genes increased in ICAM. In 2022, researchers developed a new mouse model of late reperfusion transient IS, allowing them to create a comprehensive profile of immune cell populations in the brains of aged mice affected by stroke at single‐cell resolution [100]. At least six microglial subsets were discovered in the aged brain following stroke. Among them, MG6 cells expressed high levels of *Cxcr2*, *S100a8*, interleukin (IL)‐1β, and matrix metallopeptidase 9 (MMP9), demonstrating a unique “neutrophil‐like” phenotype and were considered to represent a stroke‐specific state of microglia in the aged brain after stroke. Furthermore, microglia in the aged brain poststroke have been found to significantly upregulate *interferon alpha‐inducible protein 27 like 2A* (*Ifi27l2a*) [101]. The upregulation of *Ifi27l2a* expression is consistent with increased expression of proinflammatory cytokines such as *Il1b*, *Cst7*, and *Lyz2*, suggesting that *Ifi27l2a* may be involved in promoting inflammatory responses following stroke. Hemizygous deletion of *Ifi27l2a* (*Ifi27l2a^±^* mice) reduced cerebral infarct volumes, neuroinflammation, and motor function deficits, indicating that *Ifi27l2a* is a key mediator of stroke‐ and aging‐related neuroinflammation (Figure 2A).

Blood–brain barrier (BBB) damage is another marker of early brain damage in IS [102], leading to the entry of numerous peripheral substances and immune cells into the brain parenchyma and eventually aggravating the neurological function deficit [103]. Translocation and degradation of tight junction proteins mediated translocation across cells and paracellular barrier opening are two crucial modes of permeability [104]. Leucine rich alpha‐2‐glycoprotein 1 (LRG1) is considered an induced signaling molecule following cerebral ischemia/reperfusion (I/R) injury [105]. A 2023 study found that LRG1 deletion reduced brain edema and infarct size following cerebral I/R injury and improved neurological function [106]. ScRNA‐seq analysis revealed that LRG1 knockout could promote the BBB by upregulating tight junction proteins, such as claudin 11, integrin β5, protocadherin 9, and annexin A2. Peripheral immune cells exhibit a dual role in IS, influenced by the microenvironment and the poststroke time window [107, 108]. Single‐cell transcriptome sequencing of peripheral blood from mice at various time points after I/R identified a monocyte subset with high expression of *cathepsin S* (*Ctss*). After *Ctss* knockout, the infarct area, neurologic function scores, apoptosis, and vascular leakage were significantly reduced. Additionally, *Ctss* could disrupt BBB by binding to the junctional adhesion molecule family proteins to cause their degradation. Furthermore, analysis of three transcriptome sequencing datasets from mouse MCAO/reperfusion (MCAO/R) mice identified eight genes that were downregulated in endothelial cells [109]. The transcriptome analysis of the BBB (cortex) and non‐BBB (lung) endothelial from E13.5 mice identified 2102 genes that exhibited upregulation and may be associated with the integrity of the BBB. By intersecting the downregulated eight genes with the 2102 BBB‐related genes, researchers found that *solute carrier family 22 member 8* (*Slc22a8*) was specifically expressed in endothelial cells and pericytes, and its expression significantly decreased after MCAO/R. The use of a lentivirus carrying Tie2 to exogenously overexpress *Slc22a8* improved the levels of *Slc22a8* and tight junction proteins after MCAO/R. This intervention also reduced BBB leakage and activated the Wnt/β‐catenin signaling pathway, suggesting that the downregulation of *Slc22a8* induced by MCAO/R may be a crucial mechanism in BBB disruption (Figure 2B).

Directing at the subacute phase of IS, researchers utilized scRNA‐seq and smFISH to uncover the diversity of microglia and monocyte‐derived cells [110]. Monocyte‐derived cells were observed to differentiate along two primary trajectories poststroke: one giving rise to macrophages with pronounced phagocytic and metabolic characteristics, and the other differentiating into dendritic cells (DCs) endowed with antigen‐presenting capabilities. These cells exhibit specific spatial distributions within the poststroke brain and play a pivotal role in the immunomodulatory processes of brain repair by regulating their transcriptional profiles and differentiation trajectories. Furthermore, an increase in both microglia and monocyte‐derived macrophages was noted, sharing identical transcriptional signatures, suggesting that macrophages may compensate for microglial insufficiency. Besides, during the subacute phase, reactive glial cells surrounding the lesion core transiently proliferate and form glial scars [111, 112]. Following ischemic injury, gene expression profiles and spatial information for astrocyte clusters at different time points were revealed [113]. ScRNA‐seq and ST inferred breast cancer 1 (BRCA1) as a specific transcriptional regulator implicated in the proliferation of cluster 12, a spatiotemporally restricted subgroup associated with proliferation putatively. Moreover, researchers have integrated multiple platforms to investigate cellular heterogeneity after IS [114]. As a spatially resolved single‐cell omics platform, tissue‐digital microfluidic isolation of single cells for ‐Omics (tDISCO) can use transcriptomics and proteomics to distinguish between proximal and distal astrocytes. Using tDISCO, researchers found that proximal astrocytes exhibited differences in lipid shuttling, with an enriched expression of *apolipoprotein* (*Apoe*) and *fatty acid binding protein 5* (*Fabp5*). Similarly, another study has revealed a single‐cell resolution transcriptomics dataset that explores the acute response of the rat brain to infarction using snRNA‐seq [115]. They found that infarction‐restricted proliferating oligodendrocyte precursor cells (OPCs), mature oligodendrocytes, and reactive astrocytes share transcriptional similarities in response to ischemic injury. OPCs and reactive astrocytes are involved in shared immuno‐glial crosstalk with stroke‐specific myeloid cells. Within the perilesional zone, osteopontin (OPN)‐positive myeloid cells accumulate closely to CD44^+^ proliferating OPCs and reactive astrocytes. In vitro, OPN enhances the OPC migration but does not affect their proliferation (Figure 2C).

The loss of brain function following an IS is often difficult to recover. However, rehabilitation can partially improve the functional prognosis of patients [116, 117], suggesting the existence of cerebral self‐recovery mechanisms postinjury [118, 119]. These recovery processes are driven by various neurotrophic factors or regulators of neurite projection or synaptogenesis in the injured area [120, 121]. By integrating spatial and single‐cell transcriptomics, researchers confirmed the spatial annotation of gene expression associated with ischemia in the peri‐infarct area of the ischemic hemisphere [122]. Ligand–receptor interaction analysis in cell communication indicated the galectin‐9 to cell‐surface glycoprotein CD44 (LGALS9–CD44) as a key signaling pathway following ischemic injury, with microglia and macrophages serving as the primary sources of galectin‐9 poststroke. Extracellular vesicle‐mediated delivery of LGALS9 improved long‐term functional recovery in mice with photothrombotic stroke, while CD44 knockdown partially reversed these therapeutic effects, inhibiting oligodendrocyte differentiation and myelin regeneration. Microglial Na^+^/H^+^ exchanger‐1 (NHE1) protein, encoded by *Slc9a1*, has been implicated in white matter (WM) demyelination in IS [123]. To investigate the underlying mechanisms, researchers performed scRNA‐seq on WM tissue from conditional *Slc9a1* knockout (cKO) and wild‐type mice 3 days poststroke [96]. They identified five microglia and five oligodendrocyte subpopulations, with the MG3 and OL5 subpopulations expanded in NHE1–cKO WM tissue. These subpopulations exhibited a series of myelin sheath support genes. Additionally, CellChat [124] analysis revealed enhanced interaction of the prosaposin (in microglia)/G protein‐coupled receptor 37 (GPR37) (in oligodendrocyte) ligand–receptor pair in NHE1‐cKO brain WM, suggesting a potential mechanism for promoting OPC proliferation and differentiation, thereby enhancing WM myelin regeneration (Figure 2D). To determine the mechanism of age‐related decline in cerebrovascular and WM repair/regeneration after stroke, another study performed single‐cell transcriptomic profiling of brain tissue from young adult and aged mouses following ischemic injury [125]. The data revealed that the interaction between microglia/macrophages (MG/MΦ) and endothelial cells/oligodendrocyte lineage cells can promote tissue repair process. Notably, transplantation of young MG/MΦ into aged postischemic mice rejuvenated their behavioral performance.

#### 5.1.2 Hemorrhagic Stroke

As the main type of hemorrhagic stroke, ICH is characterized by bleeding into the brain parenchyma, with mechanisms of its damage involving the complex interplay between edema, inflammation, iron‐induced injury, and oxidative stress [126]. Based on scRNA‐seq and ST, a recent study discovered that during the acute phase following ICH, myeloid cells and lymphocytes associated with macrophages exhibit close interactions in the choroid plexus region of the brain ventricles [127]. Specifically, *Lgmn* ^+^Macro^−^T cells activate microglia through the secreted phosphoprotein‐1 (SPP1)–CD44 pathway, leading to the release of proinflammatory cytokines and chemokines, thereby exacerbating local inflammatory responses. Similarly, by analyzing the single‐cell suspensions from the hemorrhagic region of the ipsilateral striatum in a WM hemorrhage (WMH) model mouse [128], researchers revealed that post‐ICH WMH displayed a transition of homeostatic microglia into proinflammatory, anti‐inflammatory, and proliferative states, thereby affecting lipid metabolic pathways. Besides, myeloid cells amplified the expression of chemokines associated with ferroptosis pathways. Macrophages exhibited M2 phenotype predominance, characterized by their anti‐inflammatory properties. An increase in OPC proliferation aligned with enhanced ribosomal signaling, suggesting a potential reparative response following WMH.

Post ICH, perihematomal edema (PHE) encompasses complex pathophysiological mechanisms that are currently poorly understood. Given the role of PHE in secondary brain injury, the development of effective immunomodulatory therapies targeting PHE may offer a novel therapeutic approach for ICH. ScRNA‐seq was conducted on a small amount of edematous tissue surrounding the hematoma from three groups of patients (0–6, 6–24, and 24–48 h post‐ICH) with basal ganglia hemorrhage, revealing a comprehensive landscape of various immune cell populations in human PHE tissue at a single‐cell level [129]. During the progression of PHE, a SPP1 pathway was identified as a key mechanism for communication among microglial subclusters. Additionally, OPN produced by microglia can regulate the immune environment in PHE tissue by interacting with CD44‐positive cells. This finding expands our understanding of the immune microenvironment within PHE tissue.

### 5.2 Alzheimer's Disease

AD is a progressive neurodegenerative disease characterized by the formation of amyloid plaques and neurofibrillary tangles in the brain. While more refined classifications of AD genotypes and phenotypes are now available, the disease is broadly categorized into highly permeable autosomal dominant AD (ADAD) or sporadic AD, which arises from a complex interplay of genetic risk and environmental factors. However, the dysregulated pathways driving both sporadic AD and ADAD remain unknown, as do the molecular consequences of their slightly different complications. Single‐cell and spatial multiomics may uncover the underlying genotypes and cell networks associated with AD etiology and pathological features.

#### 5.2.1 Risk Factors

**FIGURE 3:**
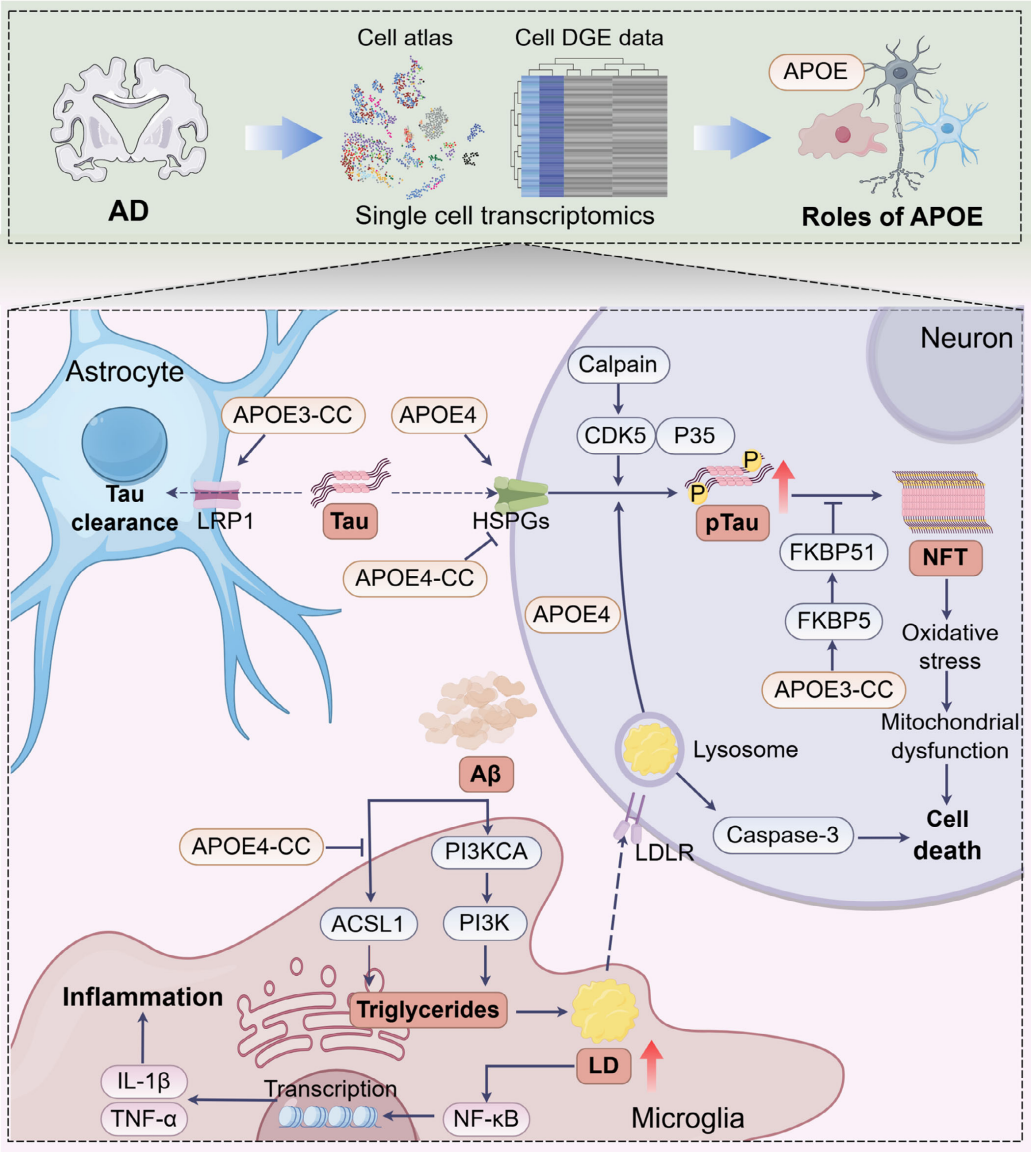
Roles of APOE genes and its variants in Alzheimer's disease. APOE4 can promote tau uptake by neurons, while APOE4–CC variant can inhibit this process, additionally, APOE3–CC variant can clear tau by promoting tau uptake by astrocytes. In neurons, tau protein is phosphorylated and polymerized to form neurofibrillary tangles (NFTs), and APOE3–CC variant can activate FKBP51 to inhibit the aggregation of ptau. The increase of NFTs can activate a series of complex stress responses, leading to neuronal death. Extracellular Aβ is mainly cleared by microglia. Aβ can activate ACSL1 and PIK3CA in microglia to synthesize triglyceride, thereby promoting lipid droplet (LD) formation. APOE4–CC variant can inhibit the activation of ACSL1 by Aβ. On the one hand, intracellular LD can promote microglia‐mediated inflammation, on the other hand, LD can shuttle to the extracellular space and be engulfed by neurons, thereby promoting the phosphorylation of tau in neurons, this process is mediated by APOE4. ACSL1: acyl‐CoA synthetase long‐chain family member 1; AD: Alzheimer's disease; APOE4–CC: APOE4–Christchurch; Aβ: amyloid‐β; CDK5: cyclin‐dependent kinase 5; FKBP5: FKBP prolyl isomerase 5; HSPG: heparan sulfate proteoglycans; LD: lipid droplet; LDLR: low‐density lipoprotein receptor; LRP1: LDL receptor related protein 1; NFT: neurofibrillary tangle; PI3K: phosphatidylinositol 3‐kinase. This figure was created using Figdraw.

APOE4 is the strongest genetic risk factor for late‐onset AD and has been reported to exacerbate AD‐related pathologies [130, 131], including amyloid‐β (Aβ) plaques, tau tangles, neurodegeneration, and neuroinflammation [132, 133, 134, 135, 136]. Blanchard et al. [137] combined with snRNA‐seq and lipidomics analysis, confirmed that *Apoe4* could lead to abnormal cholesterol deposition in oligodendrocytes, thus affecting the myelination function of oligodendrocytes and leading to cognitive decline. Integrating phosphoproteomic and proteomic analyses, *Apoe4* was found to cause early disruptions in the BBB transcriptome *Apoe4* knock‐in mice compared with *Apoe3* [138]. In middle‐aged *APOE4* mice, endothelial cells upregulated *Tspan3* and *Tcf20* while pericytes upregulated *Cyr61*, increasing the expression of BBB‐degrading enzymes MMP1 and MMP3 and decreasing the expression of the extracellular matrix protein collagen 1 [139]. This dysregulation may exacerbate BBB permeability, leading to a damaged response. *APOE4* has also been linked to lipid droplet accumulation in microglia and neurotoxic microglial‐derived factors [140]. SnRNA‐seq revealed a microglial state in AD characterized by the expression of the lipid droplet‐associated enzyme *acyl‐CoA synthetase long‐chain family member 1* (*Acsl1*), with *Acsl1*‐positive microglia being most abundant in patients with AD with the *Apoe4/4* genotype. Mechanistically, Aβ induced *Acsl1* expression, triglyceride synthesis, and lipid droplet accumulation in an APOE‐dependent manner. Additionally, conditioned media from lipid droplet‐containing microglia promoted tau phosphorylation and neurotoxicity in an APOE‐dependent manner. Notably, a single residue changes between the APOE4 and APOE3 isoforms alters protein structure and function in two domains [141, 142], thereby directly or indirectly modifying the binding of APOE to various cell surface receptors [143, 144]. Recently, the homozygous *Apoe3–Christchurch* (*Apoe3–CC*) variant was reported to confer resistance to early‐onset AD caused by the PSEN1–E280A mutation [145]. Integrating snRNA‐seq data from the frontal and occipital cortices of a patient with the *Apoe3–CC* variant revealed an upregulation of LDL receptor related protein 1 in astrocytes and FKBP1B in excitatory neurons (Ex), facilitating tau uptake and spreading of tau within neurons [146, 147]. Similarly, the *Apoe4–CC* variant was found to effectively prevent APOE4‐driven AD pathology [148]. SnRNA‐seq indicated that *Apoe4–CC* mutation increased the abundance of disease‐protective cell population while reducing the abundance of disease‐associated cell population in a gene‐dose‐dependent manner, thereby preventing *Apoe4*‐induced AD pathology (Figure 3).

Obesity is another major risk factor for AD and its most common complication [149, 150]. Notably, a high‐fat obesogenic diet was reported to accelerate recognition memory impairment in five human AD‐linked mutations (5xFAD) mice [151]. In obese 5xFAD mice, hippocampal cells exhibited minimal diet‐related transcriptional changes, while the spleen's immune landscape exhibited aging‐like dysregulation of CD4^+^ T cells. Through plasma metabolite analysis, free N‐acetylneuraminic acid (NANA) was identified as the metabolite linking recognition memory impairment in mice to an increase in spleen immunosuppressive cells. snRNA‐seq revealed that mouse visceral fat macrophages may serve as a potential source of NANA. Furthermore, NANA was found to suppress CD4^+^ T cell proliferation in mice and humans, inducing immunosenescence, and accelerating recognition memory damage in 5xFAD mice. Peripheral inflammation may affect the CNS and be a factor in AD pathophysiology [152]. Mice exposed to *Staphylococcus aureus* (Staph) through long‐term intranasal exposure have been reported to display an increased amyloid plaque burden along with increased plaque‐associated microglia in amyloid precursor protein (APP)/PS1 mice [153]. To investigate whether acute peripheral Staph infection can lead to specific molecular changes at amyloid plaque sites, researchers utilized ST to define unique Aβ plaque‐associated molecular signatures for the Staph group and phosphate‐buffered saline (PBS) group. They found that many genes related to damage‐associated microglia were highly expressed in plaques from the Staph group than in those from the PBS group. These genes are involved in intracellular vesicular transport and immune signal transduction. Furthermore, compared with PBS, the expression of glial fibrillary acidic protein was higher in Staph plaques, indicating an increase in astrogliosis following acute Staph exposure.

#### 5.2.2 Amyloid‐β Deposition

Aβ is the primary component of amyloid plaques in AD, and its accumulation is considered a molecular driver of the onset and progression of AD [154]. The production of Aβ in the brain has mainly been believed to occur solely in neurons. However, a gene set enrichment analysis (GSEA) of the single‐nucleus transcriptome profile from the cortex of a patient with AD revealed a significant positive enrichment of Aβ gene sets in oligodendrocytes, including *Rab11a*, *Lrrtm3*, and *App* [155]. Single‐cell analysis of H1 embryonic stem cell line‐derived oligodendrocytes and Ex indicated that Aβ peptides derived from oligodendrocytes contributed to AD‐related amyloidosis similar to Ex. Oligodendrocytes have also been reported to promote Aβ plaque formation in the brains of AD mice by expressing *β‐site APP‐cleaving enzyme 1* (*Bace1*) [156], the sole β‐secretase responsible for Aβ production. SnRNA‐seq results revealed that the deletion of *Bace1* in oligodendrocytes upregulates the expression of genes associated with Aβ generation and clearance, such as *Adam10*, *Ano4*, *Apoe*, *Il33*, and *Sort1*. Disease‐associated astrocytes were identified in AD mice, characterized by a large increase with age [157]. In AD, DISCO–MS revealed heterogeneity of Aβ plaques in different brain regions, as well as significant involvement of *S100a*, *Ywhaz* (14‐3‐3), vesicle fusion and transport, myelin sheath function, and the complement system related family members in early AB plaque development [88]. Based on snRNA‐seq, researchers have identified that the coexpression of lncRNA‐*small nucleolar RNA host gene 14* (*Snhg14*), *myocardin related transcription factor* a (*Mrtfa*), and *Mrtfb* across multiple cell types is strongly associated with AD progression [158]. In neuronal and glial cell subpopulations coexpressing lncRNA‐*Snhg14*, *Mrtfa*, and *Mrtfb*, calcium signaling and mitogen‐activated protein kinase (MAPK) pathways were found to be significantly enriched, which have been previously linked to Aβ plaque generation [159]. Molecular docking confirmed that lncRNA–*Snhg14* can interact with both *Mrtfa* and *Mrtfb*, potentially facilitating crosstalk between neurons and glial cells.

A single‐nucleus transcriptomic study of brain microvasculature characterized the functional role of AD risk genes in the microvascular cells of the brain [160]. Integrating quantitative immunohistochemistry, researchers have determined that the pathological expression of connective tissue growth factor and secreted protein acidic and cysteine rich (SPARC) impairs AD angiogenesis by counteracting the increased expression of proangiogenic factors, including *Fgf2*, *Hif1a*, and *Angpt2*, as pathological tissue Aβ and tau levels increase. Another study on cellular senescence examined postmortem brains from nondiseased controls (NDC) and donors with AD using imaging mass cytometry and snRNA‐seq [161]. Compared with NDC, the number of glial cells immunostained for galactosidase beta and p16^INK4A^—two widely used immunohistological biomarkers of senescence—was higher in AD, with the most notable increase observed in microglia. Gene set enrichment and pseudotime trajectories revealed extensive DNA double‐strand breaks, mitochondrial dysfunction, and endoplasmic reticulum stress associated with increased Aβ, leading to premature senescence in microglia.

#### 5.2.3 Tau Aggravation

Misfolded tau is another key pathological feature in AD [162]. Currently, using scRNA‐seq and ST, researchers have acquired a deeper insight into the biomarkers associated with tau pathology. A recent study involving 337, 512 brain myeloid cells used snRNA‐seq to identify microglial populations associated with tau pathology [163]. They used expression patterns of early tau progression to identify genes whose expression was reversed along the spreading of spatial tau pathology. Moreover, they revealed the potential involvement of these “phasic” genes in the conversion of microglial subtypes to a diseased state. Notably, these transiently upregulated genes displayed significant overlap with genes exhibiting early tau pathology‐driven dysregulation, such as *Bach1* and *Prr5*, demonstrating opposite expression patterns in the early stages of AD. The suppressor of MEK1 (SMEK1) is another regulatory protein of tau pathology, with its N‐terminal ran‐binding domain facilitating the binding to *kinesin family member 2A* (*Kif2a*) [164]. Researchers have recently identified a novel neuronal cluster with neurodegenerative features in *Smek1* ^−/−^ mice through single‐cell sequencing, characterized by cytoplasmic aggregation of *Kif2a*, axonal growth defects, and impaired mitochondrial axonal transport. *Smek1* deletion in mice activated glycogen synthase kinase 3β (GSK3β) through the protein kinase B (AKT) pathway, leading to excessive phosphorylation of tau at GSK3β sites. Conversely, *Kif2a* downregulation may ameliorate tau hyperphosphorylation and axonal growth defects in sh*SMEK1* cells. Various types of neurons exhibit varying susceptibility to tau protein attacks [165]. To determine whether this susceptibility is due to differences in neuronal types or protein expression levels, scRNA‐seq profiles of mice were utilized to analyze individual cell types [166]. Tau protein was found to be highly expressed in nonsusceptible brain areas and cell types, indicating that its expression levels in brain regions are unrelated to susceptibility, while neuron type does affect their susceptibility. Furthermore, whole‐brain scRNA‐seq of human P301L tau transgenic *Drosophila* revealed significant vulnerability of dopamine (DA) neurons and enhanced resilience of GABAergic neurons in substantia nigra. Screening predicted markers for tau vulnerability and resilience identified tau toxicity modifiers related to pathways and processes, such as “synaptic, ” “neuronal excitability, ” and “intracellular Ca^2+^ homeostasis.” This indicated that genes related to prominent vulnerability/resilience to pathogenic tau are also genetic modifiers of tau‐induced neuronal dysfunction.

### 5.3 Parkinson's Disease

Prominent pathological hallmarks of PD are the loss of dopaminergic neurons (DaNs) in the substantia nigra dense body (SNc) and Lewy body formation in DaNs and cortical regions. Neuroinflammation also plays a critical role in PD pathophysiology. Single‐cell and spatial multiomics could further reveal new aspects of PD pathogenesis and identify intrinsic properties of cell types and subtypes.

#### 5.3.1 Selective Neuronal Vulnerability

DaNs demonstrate different vulnerabilities to degeneration. DaNs in the substantia nigra pars compacta (SNc) are severely affected, while ventral tegmental area (VTA) DaNs show great resistance to degeneration. To reveal cell intrinsic properties underlying the differential vulnerability of SNc and VTA DaNs, LCM‐seq has been used to reveal the transcriptional profiles of DaNs in the SNc and VTA, and bootstrapping coupled with DEseq2 demonstrate distinct differentially expressed genes (DEGs) for the SNc and VTA, which plays a crucial role in differentiating subpopulations of neurons, particularly in disease contexts [167]. The SNc markers SLIT1 and atpase sarcoplasmic/endoplasmic reticulum Ca^2+^ transporting 3 (ATP2A3) were downregulated. SLIT1 appears to block neurite extension of DaNs [168], while lower level of ATP2A3 results in increased levels of cytoplasmic Ca^2+^ [169], potentially serving as robust indicators of the vulnerability of SNc dopamine neurons. By contrast, STRING analysis highlight differences in regulation of mitochondrial stability, apoptosis, neuronal survival, cytoskeleton regulation, extracellular matrix modulation as well as synapse integrity, which could explain the relative resilience of VTA DaNs [167]. Moreover, scRNA‐seq and ST identified a single subtype confined to the ventral tier of SNpc was strongly enriched in the SOX6_AGTR1 pathway, which was highly susceptible to loss in PD. Epigenomic profiling (ATAC‐seq) revealed accessible chromatin regions near nuclear receptor subfamily 2 group f member 2 (NR2F2) and tumor protein p53 (TP53) in these cells, linking genetic risk to transcriptional dysregulation [6]. TP53 has been implicated in motor neuron death [170] while overexpression of NR2F2 has been found to repress expression of genes encoding cytosolic aldehyde dehydrogenases, which enhances oxidative stress and promotes mitochondrial dysfunction in PD [171]. Besides, a calcium‐dependent secretion activator 2 (CADPS2)^−high^ subtype believed to be a degenerated DaN in PD was identified [172]. CADPS2 has previously been linked to to catecholamine uptake and genetic PD [173, 174], but the role of CADPS2 in the pathogenesis of PD still remains unknown.

Nevertheless, it remains unclear when and how this diversity among the neurons is generated. ScRNA‐seq allows tracking later maturation and continuous generation of gene expression diversity in midbrain dopaminergic(mDA)neurons to provide a comprehensive transcriptomic map describing mDA neuron maturation [175]. Researchers analyzed mDA neurons in Pitx3eGFP mice at six different time points from embryonic days 13.5 to postnatal days 90 by scRNA‐seq131. Validated by histological characterization, network analysis divided the defined seven neuronal subpopulations into two major branches of neurons expressing paired like homeodomain 3 (PITX3). Five of them expressed DA markers, while two expressed glutamatergic and GABAergic markers, respectively [175].

#### 5.3.2 Lewy Pathology

Progression of Lewy pathology to cortical regions is strongly associated with dementia of the most severe symptoms of PD [176, 177, 178, 179]. ST revealed molecular changes in cortical neurons susceptible to Lewy pathology and aggregates [180]. Layer 5 intratelencephalic and Layer 6b neurons, as Ex, are vulnerable to developing Lewy pathology. Lewy‐associated molecular dysfunction from aggregates revealed that neurons with aggregates downregulate mitochondrial, synaptic, ubiquitin–proteasome, cytoskeletal, and endo‐lysosomal genes while upregulating DNA repair and complement/cytokine genes. Recent research has drawn similar conclusions through independent analysis and imaging of ST datasets [181]. Researchers reveal that Layer 5 intracortical neurons are particularly vulnerable to Lewy pathology and display overexpression of *Snca*. However, this accounts for some but not all variance in pSyn pathology. *Plk2* expression indicates vulnerability to cortical and hippocampal pSyn pathology in α‐syn‐transgenic mice. It may have a potential for explaining differences in pSyn vulnerability with some cell types, as researchers observed a significant difference in *Plk2* expression between L5 IT Ex, *pSyn* ^+^/human synuclein alpha (hSNCA)^−^ low and *pSyn* ^−^/hSNCA^−^ high cells. Consequently, ST discloses the definite location and explains the molecular patterns of cortical neurons with vulnerability to α‐synuclein pathology in PD.

#### 5.3.3 Neuroinflammation

**FIGURE 4:**
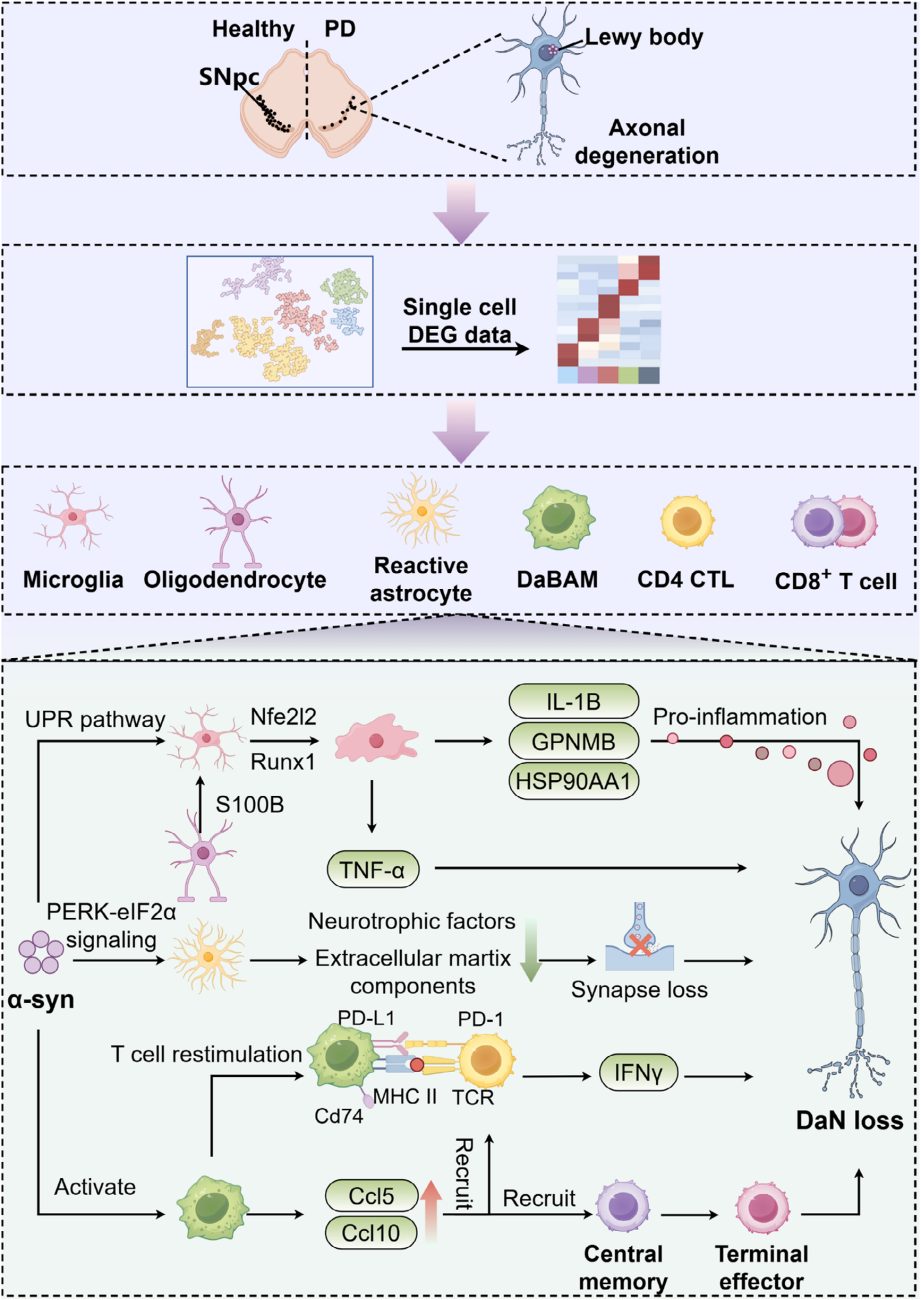
Mechanism of α‐syn and neuroinflammation in Parkinson's disease revealed by Single‐cell and spatial multiomics. PD is commonly characterized by a loss of DaNs in the SN and the presence of α‐syn Lewy pathology. Glial cells and immune cells are found express specific transcriptional programs and pathways associated with PD. Microglia are activated by UPR pathway and activation is probably regulated by NFE2L2 and RUNX1 in MPTP model. Activated microglia highly express IL‐1β, GPNMB, and HSP90AA1 leading to proinflammation in IPD. S100B‐high oligodendrocyte overexpress S100B that mediate RAGE/TNF‐α pathway, causing neuronal loss. PERK–eIF2α signaling turns astrocyte into UPR‐reactivity state, which reduces production of neurotrophic factors and extracellular matrix components thus leading to synapse loss of DaNs. α‐expression leads to a damage‐associated activation state of BAMs, which express genes for T cell recruitment, antigen processing and presentation. CD4^+^ CTLs remarkably expand and highly express cytotoxic genes, while CD8^+^ T cell undergo a continuous transition from central memory to terminal effector T cells with cytotoxicity. CTL: cytotoxic T lymphocyte; DEG: differentially expressed gene; DaBAM: disease‐activated border‐associated macrophage; DaN: dopaminergic neuron; GPNMB: glycoprotein nonmetastatic melanoma protein b; HSP90AA1: heat shock protein 90 alpha family class a member 1; IFNγ: interferon gamma; IL‐1β: interleukin‐1 β; MHC I: major histocompatibility complex class I; Nfe212: nuclear factor erythroid 2‐related factor 2; PD: Parkinson's disease; PD‐L1: programmed death‐ligand 1; PD‐1: programmed cell death protein 1; PERK–eIF2α: protein kinase r‐like endoplasmic reticulum kinase‐eukaryotic initiation factor 2 alpha; RUNX1: runt‐related transcription factor 1; S100B: s100 calcium‐binding protein beta; TCR: T‐cell receptor; TNF‐α: tumor necrosis factor alpha; UPR: unfolded protein response. This figure was created using Figdraw.

Neuroinflammation is one of the established pathogeneses of PD. Microglia, an immune cell that innately resides in the brain, develops and increases the release of IL‐1β and tumor necrosis factor‐α (TNF‐α) cytokines [182], which are involved in PD progression. Anti‐TNF‐α therapy may protect DaNs [183]. Recently, a study identified the activation states of microglia in the N‐methyl‐4‐phenyl‐1, 2, 3, 6‐tetrahydropyridine (MPTP) model, which may be mediated by two key transcriptional factors, nuclear factor erythroid 2‐related factor 2 (NFE212) and runt‐related transcription factor (RUNX) [184]. The idiopathic PD midbrain was obtained by single‐nucleus RNA sequencing, revealing panglial activation as a central mechanism in the pathology of movement disorders [172]. An association between inflammatory cell activation in the inflammatory response and PD pathogenesis has been demonstrated. Pathway analysis found that microglia in the substantia nigra enriched the risk of PD. It strongly correlated with leucine‐rich repeat kinase 2 (LRRK2), promoted the formation of inflammasomes, and was characterized by elevated levels of *Il‐1β*, *glycoprotein nonmetastatic B* (*Gpnmb*), and *Hsp90aa* [172]. Simultaneously, microglia and other glial cells have been involved in PD. Reactive astrocytes overexpress *Cd44* under the influence of upstream unfolded protein response (UPR) pathway induced by microglia [185]. The enhancement of cytokine release by both may promote higher expression of S100 calcium binding protein B (S100B) in oligodendrocytes. Furthermore, S100B has been found to be associated with neurodegeneration [186]. In PD, researchers have discovered that ablation of S100B protects against MPTP‐induced toxicity by preventing a combination of receptor for advanced glycation endproducts in microglia to reduce TNF‐α [187]. Oligodendrocytes have also been linked to the genetic risk factors associated with PD, with the PD gene *Lrrk2* being highly expressed in oligodendrocytes [188]. Moreover, acquired immunity plays a crucial role in neuroinflammation and the progression of PD. Despite the activation of glial cells, acquired immunity contributes significantly to immune system dysfunction in PD. First, scRNA‐seq and T cell antigen receptor (TCR) profiling discovered that CD4 cytotoxiclymphocytes (CTLs) expand remarkably in PD, originating from Th1 cells. Moreover, CD8^+^ T cells demonstrate continuous progression from central memory to terminal effector T cells, which are also highly express genes of cellular chemokines, adhesion molecules, and their receptors (CCL4, CCL5, CX3CR1, CD99, and SELPLG) [189]. Peripheral B cell subtypes were also involved in the pathogenesis of PD. New insights from scRNA‐seq and B cell receptor sequencing indicate while the overall level of B cells in PD patients remains unchanged or may even decrease [190], the structure of the B‐cell subsets undergoes significant alterations. Among them, naïve B cells were significantly reduced, while a subset of unswitched memory B cells was the most significantly increased. Multiple B cell activation‐related genes such as *Fcrl5*, *Egr1*, and *Cd86* were highly expressed, which may be involved in the production of infiltrative antibodies and damage DaNs by humoral immunity in PD. Additionally, a significant clonal expanded memory B cell population upregulated major histocompatibility complex II (MHC II) genes (*HLA‐drb5*, *HLA‐dqa2*, and *HLA‐dpb1*) and transcription factor activator protein 1 (AP‐1), revealing the activation of B cells and enhancement of antigen presentation capacity of B cells. Finally, the study identified preferential V and J gene fragments of B‐cell receptors in patients with PD as evidence of PD convergent selection. As central mediators, border‐associated macrophages (BAMs) are correlated with peripheral immune systems. Activated by α‐syn, BAMs transition into a “disease‐activated” state (DaBAMs). In this state, they express high levels of genes associated with T cell recruitment (*Ccl5* and *Ccl10*), antigen processing and presentation (*H2‐Aa*, *Cd74*, and *Cd274*), and remodeling of the extracellular matrix (*Mmp14*). Moreover, they are responsible for the recruitment and restimulation of CD4^+^ T cell antigen, which is essential for α‐syn‐mediated neuroinflammation [191]. Notably, the loss of CD4^+^ T cells is neuroprotective [192]. Consequently, the peripheral and tissue‐resident immune systems are vital in understanding the pathogenesis of PD. The interaction between peripheral and tissue‐resident immune systems may be a novel insight to better understand functions of immune system in PD pathology (Figure 4).

### 5.4 Glioblastoma

As an aggressive brain tumor, GBM is considered a persistent challenge in contemporary oncology. Although patients with GBM can receive intensive treatments such as temozolomide chemotherapy,  surgical resection, and radiotherapy [193], GBM inevitably recurs. This poor prognosis is highly associated with the heterogeneity of GBM cells, tumor microenvironment (TME), immune system dysfunction, and genetics. Single‐cell and spatial multiomics offers an opportunity to characterize these cells precisely at a single resolution.

#### 5.4.1 Cellular Heterogeneity

GBMs generally contain at least three different subtypes of typical cells. Every subpopulation varies significantly in their relative frequency in individual tumors. Most GBMs are primarily characterized by one particular subtype. A study combined scRNA‐seq of 20 adult and eight pediatric GBM cases [194], scRNA‐seq and lineage tracing of GBM models, and analysis of 401 TCGA bulk specimens, identifying four main subtypes: neural progenitor‐like (NPC‐like), oligodendrocyte‐progenitor‐like (OPC‐like), astrocyte‐like (AC‐like), and mesenchymal‐like (MES‐like). On the basis of the above research, researchers link high epidermal growth factor receptor (EGFR) amplification to tumors rich in AC‐like cells, platelet‐derived growth factor receptor alpha (PDGFRA) amplification to those with abundant OPC‐like cells, and cyclin‐dependent kinase 4 (CDK4) amplification to tumors with a predominance of NPC‐like cells. Tumors with significant MES‐like cells were characterized by changes of neurofibromin 1 (NF1), infiltration by immune cells, and hypoxia [195]. Similarly, another research elucidated the spatial architecture of GBM and analyzed the spatially resolved transcriptional heterogeneity [196]. By deconvoluting cellular composition, researchers identified five spatially distinct transcriptional programs: The “radial glia” and “reactive‐immune” programs, associated with radial‐glia and inflammation‐related genes, respectively; “development” and “spatial OPC” programs, coinciding with “developmental” signature and NPC‐ and OPC‐like tumor‐cell subgroups; and “reactive‐hypoxia” correlated with hypoxia‐response and glycolytic genes.

Interestingly, glioma stem cells (GSCs) can be designated as any one of these four subtypes. Research indicates that implanting xenografts enriched with cells in specific states into mice results in tumors that preserve the initial cellular ratios of those states. This finding suggests that these states possess self‐replication and differentiation capacities [31]. Another study associated these cellular states with established GSC markers. Marker expression levels were found to be highest for *Egfr* in AC‐like cells, *Cd133* in OPC‐like cells, *Cd24* in NPC‐like cells, and *Cd44* in MES‐like cells, indicating the importance of these markers in distinguishing GSC subtypes [197]. Similar results were concluded by Bhaduri et al. [198], which identified a population of outer radial glia (oRG) cells in GBM resembling those found in normal cortical development, characterized by mitotic somal translocation or “jump‐and‐divide.” Concurrently, transcriptionally diverse cell clusters with RG and developmental gene expression are identified in adult human GBMs, exhibiting unique mitotic behaviors of normal glia cells. These clusters are alternative in quiescent and cycling states, suggesting that radial glia cells may represent the cells of origin or the CSCs at the top of the GBM cell hierarchy [199].

To gain a deeper comprehension of the association between the cerebral cancer cells and the corresponding stages of neural development, a study mapped the developmental stages of the mouse cerebrum from embryonic day 12.5 to postnatal day 365 [200]. This study was conducted using single‐cell transcriptomics on over 100, 000 cells, and this atlas was compared with single‐cell data from over 100 glial tumors. The results revealed that tumor cells exhibit expression patterns intersecting with the temporally constrained embryonic radial glial precursors and their immediate descendant lineages.

#### 5.4.2 Cellular Plasticity

**FIGURE 5:**
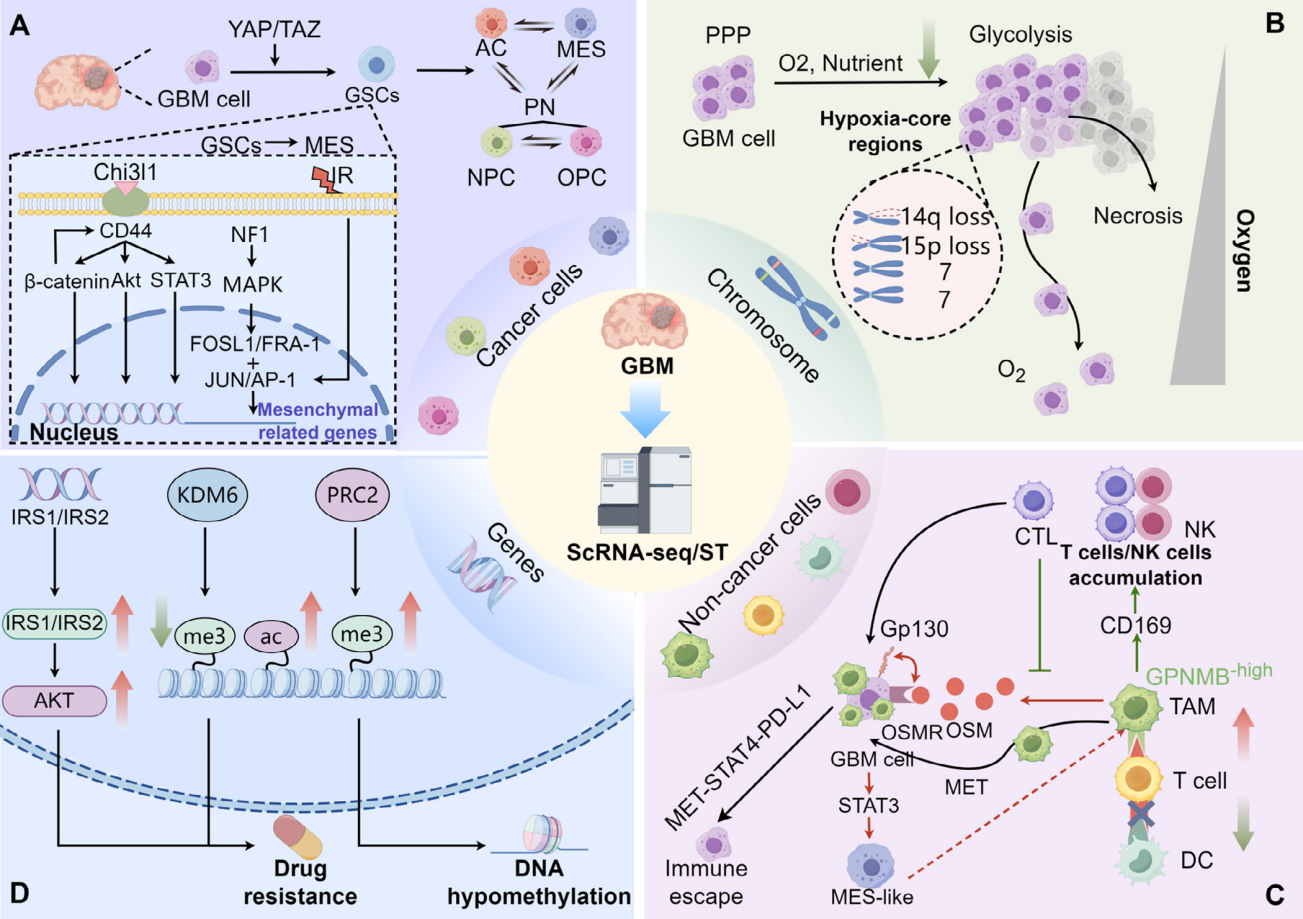
Single‐cell and spatial multiomics reveal cellular changes and mechanisms of glioblastoma. (A) Plasticity, GBM cells can be assigned as NPC‐, OPC‐, AC‐, and MES‐like states, and a single tumor cell potentially generates all four states, among which ChI3L1 and AP1 drive GSCs toward a mesenchymal expression profile. (B) Hypoxia, ST found hypoxia forces the adoption of metabolic programs of growing GBM cells from PPP toward glycolysis, subsequently leading to migration to normoxic regions, and hypoxia response and migration signatures show inversely directed spatial trajectories. (C) Immunosuppression, GPNMB‐high macrophage can ineffectively retain T cells from activating by DCs. Macrophages express a mesenchymal program, inducing a MES‐like state of GBM cells, which also facilitate a transition of macrophages into the MP‐MES state. Furthermore, macrophages enforce glioma immune evasion. However, CD169‐high macrophages promote antitumor immunity against GBM via increasing accumulation of T cells and NK cells. (D) Epigenetics, GSCs reversibly transition to persistent state with drug resistance, which upregulate developmental programs dependent on H3K27 demethylases KDM6A/B and Notch signaling. Meanwhile, copy number of IRS1 or IRS2 are amplified, and activate insulin and AKT signaling programs, which lead to dasatinib resistance of GSCs. GSCs exhibit PRC2 target hypomethylation. PRC2 catalyze H3K27me3 deposition to repress lineage‐specific developmental genes in GSCs for stemness maintenance. AC: astrocyte‐like; AKT: protein kinase B; AP‐1: activator protein 1; CD44: cluster of differentiation 44; CD169: sialic acid‐binding immunoglobulin‐like lectin 1; CTL: cytotoxic T lymphocyte; DC: dendritic cell; FOSL1/FRA‐1: FOS like 1/FOS related antigen 1; GBM: glioblastoma; GPNMB: Glycoprotein nonmetastatic melanoma protein B; GSCs: glioma stem cells; Gp130: interleukin‐6 signal transducer glycoprotein 130; IR: ionizing radiation; IRS1: insulin receptor substrate 1; IRS2: insulin receptor substrate 2; JUN: jun proto‐oncogene; KDM6: lysine‐specific demethylase 6; MAPK: mitogen‐activated protein kinase; MES‐like: mesenchymal‐like; MET: mesenchymal–epithelial transition factor; NF1: neurofibromin 1; NK: natural killer cell; NPC: neural progenitor cell; OPC: oligodendrocyte precursor cell; OSM: oncostatin M; OSMR: oncostatin M receptor; PN: proneural; PPP: pentose phosphate pathway; PRC2: polycomb repressive complex 2; STAT3: signal transducer and activator of transcription 3; TAZ: transcriptional coactivator with PDZ‐binding motif; TAM: tumor‐associated macrophage; YAP: yes‐associated protein. This figure was created using Figdraw.

In addition to cellular heterogeneity, GBM cells exhibit another critical characteristic: state plasticity. GBM cells are able to switch between different cell states; however, each GBM typically has a high abundance of one or two of the four cell states, which is significantly influenced by the genetic factors [194]. However, the molecular factors that characterize GSCs in their native state are still not well understood. Yes‐associated protein/transcriptional coactivator with PDZ‐binding motif (YAP/TAZ) coactivators were identified to regulate cell states by reconstructing the regulatory networks of GSCs [201]. YAP/TAZ are essential for establishing GSC properties in primary cells downstream of multiple oncogenic lesions, and for tumor initiation and maintenance in vivo across various mouse and human GBM models. Therefore, their inhibition irreversibly locks differentiated GBM cells into a nontumorigenic state. Additionally, a high degree of plasticity is responsible for the failure of precision therapies that induce recurrent GBM. Recent studies have revealed that recurrent GBMs feature a shift to a mesenchymal phenotype. Researchers used scRNA‐seq and RNA velocity analyses and identified that incubating patient‐derived GSCs with recombinant chitinase 3 like 1 (CHI3L1) induces significant transitions of cells toward clusters with mesenchymal transcriptomic profiles. The CHI3L1‐CD44 interaction at the GSC surface activates pAKT and p‐β‐catenin signaling pathways and induces CD44 expression in a promesenchymal feed‐forward loop, thereby promoting mesenchymal transition. Besides, ATAC‐seq revealed that CHI3L1 increases the accessibility of promoters containing Myc‐associated zinc (MAZ) finger protein transcription factor footprint. MAZ inhibition downregulated a set of genes highly expressed in cellular clusters and exhibited significant cell state transitions following CHI3L1 treatment. MAZ deficiency mitigated the CHI3L1‐induced increase of GSC self‐renewal [202]. Another study identified that the mesenchymal state is mediated by AP‐1 [203]. During IR treatment, AP‐1 positively regulates the MES hallmarks of inflammation, invasiveness, and IR resistance by cis‐regulatory enhancers. Moreover, the AP‐1 transcription factors, including JUN, JUNB, and JUND, are necessary for the transcriptional activity of fos‐related antigen 1 (FRA‐1) through heterodimerization. As the downstream of the NF1–MAPK–FOS like 1 (FOSL1) signaling pathway, FOSL1/FRA‐1 directly regulates the expression of MES genes by binding to their promoters, including *Cd44*, *Plau*, *Tnc*, and *Itga5* [204]. Loss or mutation of the NF1 copy number increases RAS/MAPK activity, thereby modulating FOSL1 expression, which is critical in regulating MES GBM (Figure 5A).

#### 5.4.3 Hypoxia

Brain tumors exhibit transcriptional adaptations similar to healthy brain tissue, with the local microenvironment significantly influencing tumorigenesis, including cellular communications and metabolism. These are crucial to the dynamic adaptation of brain cancers, facilitating growth, infiltration, and therapy resistance [204]. Hypoxia leads to the forced metabolic deterioration that drives transcriptional adaptation and genomic instability, including altered expression of DNA‐damage‐response and ‐repair genes [205, 206]. This leads to the inhibition of recombination‐mediated repair of DNA double‐strand breaks, increased mutation rates, and copy number alterations (CNAs) [207]. Hypoxia also induced hypermethylation in cell lines with unmethylated O‐6‐methylguanine–DNA methyltransferase promoters and shifted metabolism from the pentose phosphate pathway to glycolysis [208], which facilitated tumor cell migration to normoxic areas [209]. Ravi et al. [210] investigated the spatial correlation between metabolic changes and specific CNAs using MALDI Fourier‐transform ion cyclotron resonance imaging mass spectrometry and scRNA‐seq. They identified three key metabolic subgroups: the pentose phosphate pathway, glycolysis and amino sugar metabolism, and phosphoadenylate metabolism. Hypoxic regions, particularly hypoxia‐core areas, exhibited significant chromosomal changes, including losses of 15p and 14q and gains in chromosome 7, potentially due to subclonal selection. Additionally, hypoxia response and migration signatures were found to have opposed spatial trajectories, indicating that many hypoxia‐affected cells undergo apoptosis, contributing to the necrosis characteristic of GBM (Figure 5B).

#### 5.4.4 Immunosuppression

Cellular interactions between tumor cells and the immune system have increasingly been focused. Tumor‐associated macrophages (TAMs) are essential for promoting tumor progression and including immunosuppression in GBM [211]. The authors identified mesenchymal GBM subtypes expressed higher T cells and myeloid cell markers by Bulk‐seq analyses of TCGA datasets and revealed a strong correlation between macrophages and T cells [212]. They disclosed that *Gpnmb*‐high macrophage subpopulations could ineffectively retain T cells from activating DCs by competing with DCs. Therefore, targeting the *Gpnmb*‐high macrophage subset may benefit in controlling GBM plasticity and facilitate T cell‐based immunotherapy. Wang et al. [213] found that patients with macrophage enrichment exhibited significantly worse survival, and the chemotaxis of macrophages was promoted by mesenchymal–epidermal transition (MET) factor overexpression. Meanwhile, MET induced upregulation of programmed cell death protein ligand 1 (PD‐L1) expression by signal transducer and activator of transcription 4 (STAT4). It is believed that PD‐L1 blockade activates T cells and TAMs, and tumor cells frequently promote their escape from the immune system by upregulating the ligand for programmed cell death protein 1 (PD‐1) and PD‐L1. Additionally, neoadjuvant PD‐1 blockade was found to activate T cells and cDC1, inducing the production of chemotactic and cytolytic factors and the generation of exhausted T cell progenitors [214]. This therapy also systemically activated and expanded T cell clones in the periphery. However, macrophages and monocytes still encompassed the majority of infiltrating immune cells, leading to increased T cell trafficking and immunosuppressive activity. Moreover, macrophages induced proneural‐to‐mesenchymal transition (PMT) [212], which is considered a main cause of radioresistance and tumor recurrence during the malignant progression of GBM [215]. This effect of transition is mediated by macrophage‐derived oncostatin M (OSM), which interacts with its receptors (OSMR or LIFR) in complex with glycoprotein 130 (GP130) and activates STAT3 [216]. A similar relationship was found between intratumoral neutrophils and PMT [217]. Neutrophil‐derived TNF‐α directly drives mesenchymal transition in platelet‐derived growth factor B‐driven primary GBM cells [218]. Interestingly, research validated that cytotoxic T‐cells were enriched in TCGA‐MES tumors. T‐cell‐mediated killing of GBMs might be more efficient against MES‐like states. Another study investigated that the *Cd169* ^+^ macrophages derived from blood monocytes produce proinflammatory chemokines and promote accumulation of T cells and natural killer (NK) cells, contributing to antitumor immunity against GBM [219]. Furthermore, a robust correlation was found between NK‐cell‐derived interferon‐γ (IFN‐γ) and the accumulation of *Cd169* ^+^ macrophages, indicating its potential as a promising therapeutic target. The emergence of *Cd169* ^+^ macrophages provides novel insights into TAMs (Figure 5C).

#### 5.4.5 Epigenetics

Genetic drivers shape the distribution of glioma cell states, affecting the quantity and composition of GBM cells. These drivers also modulate the immune microenvironment, dictating the composition of microenvironmental myeloid cells [220]. Notably, epigenetic mechanisms are crucial for maintaining GSC plasticity and are linked to GBM drug resistance. Polycomb repressive complex 2 (PRC2) has been reported to directly correlate with DNA methylation [221]. PRC2 targeted hypomethylation marks GSCs, sustaining their stemness and reactivation potential [222]. In GBM drug resistance, amplifications of IRS2 and IRS1 confer resistance to dasatinib in PDGFRA‐amplified GSCs, which can reversibly transition to a slow‐cycling state dependent on Notch signaling [217]. These persistent GSCs upregulate H3K27 demethylases KDM6A/B, contributing to H3K27me3 alterations in dasatinib‐treated GSCs [30]. Therefore, the interaction between epigenetics and genetics is crucial for maintaining GBM plasticity and drug resistance along with regulating the distribution of glioma cell states (Figure 5D).

### 5.5 Autism Spectrum Disorder

Although ASD is typically considered in the context of childhood, they may represent lifelong conditions. A fundamental shared characteristic of ASD is the disruption of synaptic circuit formation and balance [223]. For genetically complex disorders like ASD, it remains unclear how diverse genetic risk factors converge on shared transcriptional alterations. Based on established evidence linking contactin‐associated protein‐like 2 (Cntnap2) to ASD [224, 225], a study integrated quantitative proteometabolomic data from Cntnap2 KO mice with multiomics data from ASD patients and forebrain organoids, revealing Cntnap2‐dependent molecular networks in ASD [226]. ScRNA‐seq analysis identified Ex as the major cell type in Cntnap2‐dependent ASD. Ex‐specific DEGs were significantly enriched in axonal structure, synaptic vesicle function, and oxidative phosphorylation. Compared with controls, Cntnap2 KO mice exhibited enhanced lipid metabolism (upregulation of ACAA2, ACADM), but dysregulation of glycolysis and the electron transport chain; downregulation of proteins about synaptic vesicle trafficking, including vesicle recycling, docking, and exocytosis (VAMP2, RAB3A, SNAP25); disruption of cytoskeletal regulatory pathways (RAC/CDC42/PAK, MAPK/ERK), evidenced by downregulation of key molecules like PAK3 and GAP43. Collectively, these findings demonstrate that *Cntnap2* deficiency drives ASD phenotypes by impairing mitochondrial function, synaptic vesicle transport, and axonal development within Ex. Hypoxic conditions are considered an important pathological mechanism in ASD, hypoxia‐inducible factor‐1α (HIF1A) is a key participant in this mechanism [227]. Integrated module analysis and machine learning identified four HIF1A‐associated ASD hub genes: *Cdkn1a*, *Ets2*, *Lyn*, and *Slc16a3* [228]. Furthermore, maternal immune activation during pregnancy is also a significant risk factor for ASD, which is linked to the activation of IL‐6 in microglia [229]. Examination of a scRNA‐seq dataset from ASD patients revealed significant enrichment of these hub genes specifically in reactive microglia and was subsequently validated in animal models [228]. Further in vitro investigations elucidated the potential regulatory impact of IL‐6 on the expression of hub genes through the HIF1A pathway in microglia, specifically, the IL‐6/JUN/HIF1A axis regulates the expression of *Cdkn1a*, *Lyn*, and *Slc16a3* in microglia, thus participating neuroinflammation in ASD.

Recent studies have explored the differential involvement of lamina‐specific and cell type‐specific pathways in ASD. In the frontal cortex of ASD patients, significant glial cell activation is evident, characterized by a marked increase in the proportion of activated microglia and astrocytes [230]. Within microglia, phagocytosis‐related genes (*Tlr2*, *Cd68*) are upregulated. Astrocytes exhibit upregulation of inflammatory cytokines (*Tgf‐β1*). ST analysis reveals that glial activation is concentrated in superficial cortical laminae (Layer 2 and 3, L23), which is colocalized with transcriptomic dysregulation in Ex. Specifically, within the L23 Ex subtype, synaptic genes (*Neto2*, *Grm3*) are significantly downregulated, while genes associated with inflammatory pathways (*Tgf‐β2*, *Spp1*) are upregulated. Gene regulatory network analysis identifies Cux1 as a core transcription factor regulating L23 Ex and its target genes are enriched for ASD risk genes. Runx1 and Jund are identified as drivers of the microglial inflammatory response, considering the activity of Runx1 and Jund is induced by TGF‐β signals [231, 232], TGF‐β signaling may mediate neuron‐glia crosstalk, thereby amplifying the inflammatory response. Similarly, through integrated analysis of single‐nucleus multiomics data, genome‐wide association studies (GWAS), and ST (MERFISH), ASD‐risk genes are found to be significantly enriched in L6 intratelencephalic Ex within the prefrontal cortex and primary visual cortex‐specific L4 intratelencephalic Ex during the second trimester [233].

### 5.6 Amyotrophic Lateral Sclerosis

ALS is a severe motor neuron disease driven by the interplay of central and peripheral immunity. Typical pathological feature of ALS is nuclear depletion and cytoplasmic accumulation of transactive response DNA binding protein of 43 kDa (TDP‐43) [234]. As the primary vulnerable cell type in ALS, motor neurons have been found to be enriched with multiple risk genes. For instance, Thy1high extratelencephalic neurons (ETNs) exhibit elevated expression of ALS risk genes in both ALS patients and healthy individuals [235]. ST data confirm that ETNs are predominantly localized to L5 motor cortex (MCX). Beyond risk gene enrichment, ETNs significantly upregulate stress‐response genes, concurrently, oligodendrocytes downregulate myelination‐associated genes (*Cnp*, *Mbp*), while microglia upregulate proinflammatory genes (*Trem2*, *Apoe*), and these dysregulations are mechanistically linked to neuronal apoptosis, suggesting the selective vulnerability of ETNs arises not only from their intrinsic properties but also from a degenerative cascade triggered by their stressed state. Another study identified a distinct subpopulation of upper motor neurons (*Vat1l*⁺ neurons), which was also localized to L5 primary MCX and enriched with ALS risk genes [236]. Additionally, researchers observed downregulation of tight junction proteins (CLDN5, PECAM1) and HLA‐E in MCX endothelial cells. As a key regulatory factor of NK cell, HLA‐E has previously been proven to be capable of inhibiting NK‐mediated cell lysis [237]. These findings suggest that loss of HLA‐E expression in the brain endothelium may represent a mechanistic pathway for NK‐mediated BBB disruption. Interestingly, four microglial subpopulations exhibiting a DAM‐like phenotype were identified in the developing spinal cord of ALS patients [238]. GO analysis revealed their functional implication in key biological processes, including TYROBP causal network in microglia, responding to unfolded proteins, positively regulating cell death, responding to toxic substances, responding to axon injury, participating in neuron apoptotic processes, and activating T cells, provide valuable insights into the microglial profiles underlying ALS pathogenesis.

The role of peripheral blood immune cells in neuroinflammation during ALS remains controversial. A scRNA‐seq study of peripheral blood mononuclear cells from ALS patients highlighted the involvement of T helper 17 cells (Th17), effector CD8 T cells, and CD16^high^CD56^low^ mature NK cells [239]. The results showed that in rapidly progressing ALS patients, the ratio of Th17 cells (proinflammatory) to Treg cells (anti‐inflammatory) was significantly increased and positively correlated with disease progression rate; the ratio of effector CD8 T cells to naïve CD8 T cells were higher in the rapid progression group, with the ratio positively correlated with progression speed and the proportion of CD16^high^CD56^low^ mature NK cells was higher in the rapid progression group. Combining serum proteomics through a proximity extension assay, researchers identified serum inflammatory proteins positively correlated with the frequency of the above cells, such as IL‐17A–Th17 and CD94‐effector CD8 T cells. A recent study identified a pathogenic CD56^dim^ *FceR1G* ^+^ NK (NK_2) subset expanded in ALS patients, characterized by cytotoxic gene signatures (*Gzmb*, *Nkg7*) and activation of immune‐response pathways [240]. Cell–cell communication analysis revealed a unique ALS‐specific interaction network centered on HLA‐E ligands (expressed by CD8^+^ memory T cells) binding CD94:NKG2C receptors on NK_2 cells, driving proinflammatory signaling. This ligand–receptor axis promotes NK cytolytic activity and lymphocyte proliferation, suggesting peripheral immune hyperactivation. Notably, the proportion of CD56bright NK cells correlated strongly with plasma neurofilament light levels, which may implicate CD56^bright^ NK subsets in neuronal injury possibly via CNS migration. These findings highlight dynamic NK‐T cell crosstalk through specific molecular hubs, positioning peripheral NK subsets as key mediators of neuroinflammation and biomarkers of disease progression in ALS.

### 5.7 Epilepsy

Focal cortical dysplasia (FCD) is the most common cause of drug‐resistant focal epilepsy [241]. Among them, the difference between FCD type 2 (FCD II) lesions and other FCDS lies in abnormal cortical stratification, dysmorphic neurons (DNs), and balloon cells (BCs) [242], which is driven by the developmental somatic activating mutations in mammalian target of rapamycin (mTOR) pathway genes [243]. ST in FCD IIb revealed distinct molecular signatures within DNs and BCs microenvironments [244]. Evidenced by p62 accumulation, DNs region exhibited mTOR pathway hyperactivation, impaired autophagy, and ubiquitin–proteasome dysregulation, and enrichment of genes regulating membrane potential (*Gabrd*, *Ywhah*, *Snap25*), suggesting intrinsic hyperexcitability. BCs region showed pronounced neuroinflammation and complement cascade activation (*C3*, *Clu*, *Serpina3*), indicating BCs function as inflammatory hubs. Shared dysregulation of developmental growth pathways occurred in both regions. Critically, BCs‐driven complement signaling (C3/CLU) potentially modulates microglial activation and astrocyte reactivity. The molecular characteristics of DNs and BCs have also been revealed recently. In FCD IIb, somatic mTOR pathway mutations exist in various cell types [245]. For example, DNs affected by the mutations express genes about neurofilament protein (*Nefm*) and mitochondrial biomass (*Vdac1*), whose molecular characteristics are similar to glutaminergic neurons; The BCs affected by mutations express cellular senescence‐related genes (*Igfbp7*), whose molecular characteristics are similar to astrocytes. Swollen and fragmented mitochondria in DNs can be observed through electron microscope, combined with the upregulation of mitochondrial gene (*Vdac1*) expression and the increase of VDAC1 protein level, it suggests that the excessive activation of mTOR leads to mitochondrial metabolic dysregulation. These spatial mappings identify mTOR‐driven proteomic and mitochondrial homeostasis dysregulation in DNs and BCs as convergent pathways promoting epilepsy in FCD IIb.

As one of the most common types of epilepsy, temporal lobe epilepsy (TLE) is characterized by significant pathological changes in the hippocampus, typically manifested as neuronal loss, gliosis, and increased neural fiber density, collectively termed hippocampal sclerosis [246]. By integrating the results of three sequencing methods (scRNA‐seq, snRNA‐seq, and Xenium), researchers found that in the hippocampus of TLE mice, the loss of inhibitory neurons was the most severe [247]. In terms of transcriptomic alterations, pathways related to gliosis, innate immune response, cytokine response, and phagocytosis are upregulated, marked by the consistent upregulation of *Spp1*, *Trem2*, and *Cd68*, Xenium analysis showed diffuse overexpression of *Spp1* in the damaged hippocampus, indicating significant inflammation. In neurons, the neurotrophin pathway was significantly upregulated, its upstream mediator *Bdnf* is mainly expressed in Ex, with its receptors distributed between inhibitory neurons and Ex. Cell–cell communication analysis revealed the most increased interaction between Ex and microglia in the hippocampus, suggesting that excessive neuronal interactions leading to hypersynchronization may drive glial inflammation and hippocampal sclerosis. Integrated scRNA‐seq and ST in drug‐resistant epilepsy (DRE) reveal a transcriptionally distinct cortical pyramidal neuron subcluster (PY2) characterized by a senescent molecular signature [248]. PY2 highly expresses senescence‐related genes (*p21*, *Nfkbia*, *Ccl2*). Spatial validation via expansion‐assisted iterative FISH [249] confirmed enlarged soma size and colocalization of senescence markers in histopathological neurons from FCD and temporal TLE tissues. This senescence phenotype is driven by chronic seizure activity, as demonstrated in Pten^−/−^ mouse models, where mTOR‐dependent inflammatory cascades trigger neuronal cell‐cycle arrest and secretory phenotype activation. Neuro‐glial crosstalk is implicated through PY2‐derived chemokines, CCL2, which recruit microglia and amplify neuroinflammation via nuclear factor kappa‐B signaling. Crucially, mTOR inhibition through rapamycin downregulate senescence markers, highlighting cellular senescence as a mechanistic link between neuronal hyperexcitability and immune dysregulation in DRE.

## 6 Multiomics Integration Redefining Precision Medicine in CNS Diseases

Most persistent complex diseases have multicellular etiologies. Understanding the perspective of individual cells is crucial for improving our ability to identify the most effective therapeutic targets at the cellular or molecular level. Single‐cell and spatial multiomics allows researchers to identify interactions between different cells in various regions, including signaling pathways, cell adhesion, and cell‐to‐cell communication. This innovative approach reveals genes with upregulated expression levels in specific cells or subcellular structures within the area of interest, uncovering potential targets for therapeutic intervention. In the following sections, our study will provide an extensive review of the clinical application of single‐cell and spatial multiomics in discovering new therapeutic targets and their corresponding treatment strategies for CNS diseases (Table 2).

**TABLE 2:** Therapeutic targets in central nervous system diseases revealed by single‐cell RNA sequencing and spatial transcriptomics technologies.

Disease	Target	Intervention	Species	Mechanism	Therapeutic effect	References
Ischemic stroke	*Lilrb4*	*Lilrb4* overexpression	Mouse	Downregulate CCL2 to mitigate the infiltration and activation of CD8^+^ T cells	Protect against ischemia‐induced brain damage	[250]
	TNF‐α	Liposomes containing clodronate	Mouse	Deplete BAMs to reduce expression levels of TNF‐α	Reduce infarct volume and improve neurological function	[251]
	*Spp1*	KBA–Z‐GS	Mouse	Synergistically regulate the differentiation trajectory of microglia and astrocytes through *Spp1*, thus regulating the inflammatory response of microglia, and the cell metabolism and ferroptosis of astrocytes	Protect against ischemia‐induced brain damage	[252]
	CEBPB	Neutrophil membrane‐camouflaged polyprodrug nanomedicine carrying FTY720	Mouse	Modulate CEBPB‐regulated NLRP3 inflammasome activation and CXCL2 chemokine secretion to reprogram microglia to an anti‐inflammatory phenotype	Anti‐inflammatory	[253]
	FOXP3	/	Mouse	FOXP3 promotes macrophage phagocytosis by promoting cargo metabolism, thus terminating neuroinflammation caused by cell death	Anti‐inflammatory	[254]
	THBS1	Salvianolic acid B	Mouse	Inhibit the interaction between THBS1 and OTUD5, leading to reduce GPX4 ubiquitination, decrease the sensitivity of endothelial cells to ferroptosis	Inhibit BBB disruption to mitigate CIRI	[255]
	PD‐1	sPD‐L1	Mouse, human	Activate PD‐1 to transform monocytes into LY6C^lo^, CX3CR1^hi^, CD43^hi^, PD‐L1^+^ anti‐inflammatory phenotype	Reduce brain edema	[256]
	PLA2G2E	/	Mouse	PLA2G2E metabolizes phosphatidylserine to DGLA and 15‐HETrE, inducing PADI4 to initiate recovery‐associated gene expression in neurons.	Promote nervous system development and synaptic modulation	[257]
	XBP1	XBP1 overexpression	Mouse	By enhancing the healing EC phenotype, XBP1 introduces proangiogenic pathways to promote ECs proliferation and reduce ECs apoptosis.	Facilitate angiogenesis	[258]
	AMPK	Sequential treatment of compound C and metformin	Mouse, human, human iPSC	Regulate Ser 436 phosphorylation state of CBP to coordinate the shift of acetylation between SOX2 and histone H2B, and to regulate SOX2 nuclear cytoplasmic transport during reprogramming/differentiation of pericytes	Promote the transformation of pericytes into functional neurons post ischemic stroke	[259]
Hemorrhagic stroke	THBS1 CD47	THBS1 neutralizing antibody CD47 neutralizing antibody	Mouse, human	Inhibit THBS1/CD47 L‐R pair and promote STAT3/BCL‐2 signaling pathway to alleviate mLVs apoptosis	Promote injury after subarachnoid hemorrhage	[260]
	IGF1 OPN	AAV–IGF1/OPN (early‐stage) PLX3397 (late‐stage)	Mouse	Microglia‐derived IGF1 induces astrocytic scar through mTOR signaling, and OPN increases astrocyte sensitivity to IGF1. PLX3397 depletes microglia and prevents astrocytic scar formation.	Promote more protective scar formation at early‐stage ICH Reverse the overall net effect of scar changes from protective to destructive at late stage ICH	[261]
	EPAC‐1	Celastrol	Mouse	Bind to cNMP, inhibit its interaction with VDAC1, and block the opening of mitochondrial permeability transition pores to ameliorate neuronal mitochondrial dysfunction	Reduce ICH‐induced secondary brain injury	[262]
	*Cacna1c*	/	Mouse	The upregulation of *Cacna1c* enhances the function of calcium channels, leading to hyperexcitability of neurons, which enhances the production and transmission of pain signals.	Facilitate the development of neuropathic pain	[263]
Alzheimer's disease	*APOE4*	Tamoxifen Astrocytic APOE4 knockout PLX3397	Mouse	Neuronal *Apoe4* removal diminish disease‐associated subsets of neurons, oligodendrocytes, astrocytes, and microglia. Astrocytic *Apoe4* removal diminish disease‐associated subsets of neurons, oligodendrocytes, astrocytes, and microglia. Depletion of microglia has been shown to effectively mitigate *Apoe4*‐associated pathologies in AD.	Reduce Aβ‐ and tau‐induced neurodegeneration	[264, 265, 266]
	IL‐3	rIL‐3	Mouse, human	Activate IL‐3/IL‐3R L‐R pair to trigger reprogramming of microglia, endowing them the ability to gather and clear Aβ and tau aggregates	Reduce Aβ burden and inflammation	[267]
	TREM2	MK2206 ATV:TREM2 Lecanemab	Mouse, human	Reduce R47H signaling by AKT inhibition to reduce the expression of inflammatory factors in microglia Activate TREM2 signaling to improve the mitochondrial metabolic of microglia, enhancing microglial uptake of Aβ Activate of microglia to upregulate genes associated with Aβ clearance	Reduce Aβ‐ and tau‐induced neurodegeneration	[268, 269, 270]
	SMAD3 VEGFA	rVEGF	Human, zebrafish	Activate VEGFA to reduce SMAD3	Promote the blood–brain barrier integrity	[271]
	SPP1	SPP1 knockout	Mouse	Inhibit the autocrine and paracrine crosstalk signals between PVM and microglia, reducing microglia‐mediated synaptic phagocytosis	Rescue neurite outgrowth defects	[272]
	LINGO2	LINGO2 shRNA	Mouse, human iPSC	Downregulate LINGO2 to inhibit ERK signaling, thus upregulating p53‐, apoptosis‐, and cellular senescence‐associated pathways	Rescue neurite outgrowth defects	[273]
	CXCR3	MAB160	Human	Inhibit CXCL10 binding to CXCR3, thus attenuating CD8^+^ T cell infiltration and reduce the induction of interferon‐γ and neuroinflammation pathways in glial cells	Reduce neurodegeneration and inflammation	[274]
Parkinson's disease	CD99	CD99 antibody	hESC	Eliminate CPECs via FACS isolation	Promote the purity of DA progenitors	[24]
	CLSTN2 PTPRO	/	Mouse	CLSTN2 and PTPRO as specific surface markers of mDA progenitors facilitate high enrichment of mDA neurons	Exhibit high therapeutic potency in correcting motor deficits	[275]
	WNT1 FGF8B LXRs FGFRs	CHIR99021 and WNT5A CHIR99021 and FGF8B GW3965 PD0325901 and SU5402	hESC	Activate the WNT/β‐catenin and WNT/PCP/RAC1 signaling pathways Activate FGF8b signaling Activate LXRs signaling Inhibit FGFRs signaling	Promote the differentiation of hESCs into functional mDA neurons	[276]
	/	Adeno‐associated virus–intein–split–dCas9	Mouse	Reprogram striatal astrocytes into induced GABAergic neurons, and induce GABAergic neurons integrating into striatal circuits	Alleviate voluntary motor behavior aspects	[277]
	TGF‐β1	Transplantation of hOM–MSC	Mouse, human	TGF‐β1 secreted from hOM–MSC activates ALK–PI3K–AKT signaling in microglia	Enhance neural functional recovery	[278]
	GPR37L1	E.melanin	Mouse	Activate PSAP–GPR37L1 signaling pathway within astrocytes	Reduce synaptic engulfment of astrocytes	[279]
	*Cth*	Astrocytic *Cth* knockdown	Mouse, human	Knockdown of astrocytic *Cth* inhibits YAP–FOXD3 signaling pathway	Reduce the expression of neurotoxic markers in reactive astrocytes	[280]
	JAK1/2	AZD1480	Mouse	Inhibit the JAK/STAT pathway in innate and adaptive cells, downregulating genes related to antigen presentation and neuroinflammation	Reduce neuroinflammation	[281]
Glioblastoma	*Gpnmb*	/	Mouse, human	*Gpnmb*‐high macrophages hinder T cell activation by DCs through competitive interaction	Benefit in controlling plasticity and facilitating T cell‐based immunotherapy	[212]
	MET	MET knockdown	Human	Downregulate STAT4–PD‐L1 signaling activation	Decrease immune evasion	[213]
	CD169 IFN‐γ	/	Mouse, human	CD169 enhances recruitment of cytotoxic immune cells in glioblastoma IFN‐γ produced from NK cells facilitates tumor infiltration of CD169^+^ macrophage	Promote the phagocytosis of apoptotic glioma cells	[219]
	SLIT2	SLIT2‐trapping protein ROBO1Fc	Mouse	SLIT2–ROBO1/2 axis drives macrophage chemotaxis and tumor‐supportive polarization via PI3Kγ	Inhibit macrophage infiltration and tumor growth in glioblastoma mouse models	[282]
	PI3Kγ	TG100‐115	Mouse	Inhibit PI3Kγ to promote microglia/macrophage associated IL‐11 secretion, thus activating the STAT3–MYC signaling in tumor cells	Enhance the antitumor effect of temozolomide	[283]
	CLOCK OLFML3	SR9009	Mouse	Inhibit CLOCK–OLFML3–HIF1A–LGMN–CD162 axis to increase CD8^+^ T cell infiltration, activation and cytotoxicity	Promote immunosuppression	[284, 285]
	CD73	CD73 knockout	Mouse, human	Promote survival in a murine model of glioblastoma treated with anti‐CTLA‐4 and anti‐PD‐1	Hinder tumor progression and immune suppression	[286]
	JAK1/2	Ruxolitinib	Human	Reduce the release of IL‐10 to inhibit JAK/STAT pathway, and promote T cell transformation	Promote immunosuppression	[287]

Abbreviations: AAV, adeno‐associated virus; AKT, protein kinase B; ALK, activin receptor‐like kinase; AMPK, adenosine monophosphate‐activated protein kinase; APOE4, apolipoprotein E4; Aβ, amyloid‐β; *Lilrb4*, *leukocyte immunoglobulin‐like receptor B4*; BAM, border‐associated macrophage; BBB, blood–brain barrier; BCL‐2, B‐cell lymphoma‐2; *Cacna1c*, *calcium voltage‐gated channel subunit alpha1 C*; CCL2, C‐C motif chemokine ligand 2; CEBPB, CCAAT enhancer binding protein beta; CIRI, cerebral ischemia–reperfusion injury; CLSTN2, calsyntenin 2; CLOCK, clock circadian regulator; CPEC, choroid plexus epithelial cell; CTLA‐4, cytotoxic T‐lymphocyte associated protein 4; CXCL2, C‐X‐C motif chemokine ligand 2; CXCR2, C‐X‐C motif chemokine receptor 2; DGLA, dihomo‐γ‐linolenic acid; EPAC‐1, cAMP‐activated exchange protein‐1; EC, endothelial cell; ERK, extracellular regulated protein kinases; FACS, fluorescence‐activated cell sorting; FGFR, fibroblast growth factor receptor; FGF8B, fibroblast growth factor 8b; FOXD3, forkhead box d3; FOXP3, forkhead box p3; GPR37L1, G protein‐coupled receptor 37 like 1; *Gpnmb*, *glycoprotein nonmetastatic B*; GPX4, glutathione peroxidase 4; hESC, human embryonic stem cell; HIF1A, hypoxia‐inducible factor‐1α; hOM–MSC, hypoxia‐preconditioned olfactory mucosal mesenchymal stem cells; ICH, intracerebral hemorrhage; IFN‐γ, interferon‐γ; IGF1, insulin like growth factor 1; LINGO2, leucine rich repeat and ig domain containing 2; IL‐3, interleukin‐3; iPSC, induced pluripotent stem cell; JAK, Janus tyrosine kinase; KBA, 11‐keto‐β‐boswellic acid; LXR, liver X receptor; mDA, midbrain dopamine; MET, mesenchymal–epithelial transition factor; NLRP3, NLR family pyrin domain containing 3; OLFML3, olfactomedin like 3; OPN, osteopontin; OTUD5, OTU deubiquitinase 5; PADI4, peptidyl arginine deiminase 4; PD‐1, programmed cell death protein 1; PD‐L1, programmed cell death protein ligand 1; PI3K, phosphoinositide 3‐kinas; PLA2G2E, phospholipase a2 group IIE; PSAP, prosaposin; PTPRO, protein tyrosine phosphatase receptor type O; ROBO1/2, roundabout guidance receptor 1/2; ROS, reactive oxidative species; SLIT2, slit guidance ligand 2; SMAD3, SMAD family member 3; SOX2, SRY‐box transcription factor 2; *Spp1*, secreted phosphoprotein‐1; STAT3, signal transducer and activator of transcription 3; TGF‐β1, transforming growth factor beta 1; THBS1, thrombospondin 1; TNF‐α, tumor necrosis factor‐alpha; TREM2, triggering receptor expressed on myeloid cells‐2; VDAC1, voltage dependent anion channel 1; VEGFA, vascular endothelial growth factor A; WNT1, Wnt family member 1; XBP1, x‐box binding protein 1; YAP, yes‐associated protein; Z‐GS, Z‐guggulsterone.

### 6.1 Stroke

#### 6.1.1 Ischemic Stroke

The neurovascular damage following IS is primarily caused by a series of inflammatory responses triggered by damage‐associated molecular patterns and reactive oxygen species released from ischemic and hypoxic brain tissues [288]. Moreover, damage or injury to neurons in the brain during adulthood reduces their renewal and regeneration abilities. Therefore, it is pivotal to recognize that the neuroprotection concept in treating IS has shifted from reducing neuronal cell death to activating endogenous neuroprotection [289]. Based on this, the proper termination of neuroinflammation and the activation of endogenous molecules have garnered significant research interest.

Recently, *leukocyte immunoglobulin‐like receptor B4* (*Lilrb4*) in microglia has been found to play a crucial role in the immune response following IS by modulating CD8^+^ T cell infiltration and activation [250]. ST analysis revealed increased expression of *Lilrb4* in the ischemic hemisphere. Furthermore, scRNA‐seq identified a microglial cluster3, which is an ischemia‐associated microglial subcluster with elevated *Lilrb4* expression in the ischemic brain. Microglial overexpression of *Lilrb4* was found to prevent ischemia‐induced brain damage. Furthermore, T‐cell migration assays demonstrated that *Lilrb4*‐KD microglia promoted CD8^+^ T‐cell recruitment and activation in vitro. This effect was mitigated by CCL2 inhibition and recombinant arginase‐1 addition. BAMs, located at the interface between the brain and the periphery, have recently been reported participating in various brain pathologies, however, their role and mechanisms in stroke remain poorly understood. Analysis of two scRNA‐seq datasets revealed comprehensive changes in BAMs following stroke [251]. For instance, under hypoxic conditions, BAMs activated the TNF pathway, and the activity of the transcription factor *Stat3* in BAMs was significantly upregulated, suggesting that *Stat3* inhibition might be a potential strategy for treating BAM‐induced neuroinflammation. Depletion of BAMs using liposomes containing clodronate resulted in a significant reduction in cerebral infarct volume in mice, along with decreased expression levels of TNF and F4/80, indicating that BAM depletion can mitigate poststroke inflammatory responses, which provides a novel therapeutic strategy for stroke treatment. Frankincense and myrrh are two natural substances used in traditional Chinese medicine for treating cerebrovascular diseases [290], and their key components, 11‐keto‐β‐boswellic acid (KBA) and Z‐guggulsterone (Z‐GS), are the active ingredients. Single‐cell transcriptomics has revealed the synergistic mechanism of KBA and Z‐GS on IS [252]. The KBA–Z‐GS treatment can regulate the inflammatory response in microglia, with *Slc1a2* and *Timp1* being the core fate transition genes regulated by KBA–Z‐GS. Additionally, researchers have identified *Spp1* as the target gene for the synergistic protection of MCAO by KBA–Z‐GS. KBA–Z‐GS treatment promoted *Spp1* expression in microglia and astrocytes, confirming *Spp1* as the hub target of KBA and Z‐GS. FTY720 is a United Sates Food and Drug Administration‐approved anti‐inflammatory drug that has exhibited potential neuroprotective properties in ischemic brain tissue [291]. A recent study introduced a nanodrug coated in neutrophil membranes that can migrate to ischemic brain tissue and release FTY720 in situ under increased ROS levels [253]. This nanodrug can deliver 15.2 times more FTY720 to the ischemic brain and significantly reduce the risk of cardiotoxicity and infection compared with intravenous free drug administration. Furthermore, scRNA‐seq analysis found that the nanodrug reprograms microglia to an anti‐inflammatory phenotype by regulating CCAAT enhancer binding protein beta‐mediated NLRP3 inflammasome activation and C‐X‐C motif chemokine ligand 2 (CXCL2) chemokine secretion, thereby alleviating poststroke inflammation.

Forkhead box p3 (FOXP3), the master immune repressive transcription factor of Treg cell, was reported to promote macrophage phagocytosis by enhancing cargo metabolism, thereby strengthening efferocytosis to terminate cell‐death‐induced neural inflammation [254, 292]. FOXP3 expression was regulated by macroautophagic/autophagic protein degradation in resting macrophages. The initiation of LC3‐associated phagocytosis after acute IS competitively occupied the autophagic machinery, thereby allowing FOXP3 activation and FOXP3^+^ macrophages exhibiting superactive phagocytic capacity. However, immune cell infiltration can cause brain microvascular endothelium dysfunction, increase BBB permeability, and develop a driving force for vasogenic (transvascular) edema, which is detrimental to recovery [293]. For instance, integrated with scRNA‐seq, macrophages have been reported to release thrombospondin 1 (THBS1) via exosomes [255], which promotes the ubiquitination and degradation of glutathione peroxidase 4 (GPX4) by binding to OTU deubiquitinase 5 (OTUD5), thereby increasing endothelial cells’ susceptibility to ferroptosis and exacerbating cerebral I/R injury. Targeting this mechanism, salvianolic acid B can inhibit the interaction between THBS1 and OTUD5, reducing the ubiquitination and degradation of GPX4, thereby alleviating endothelial cell ferroptosis and BBB disruption. PD‐1 is an inhibitory immune checkpoint, and monocytes upregulate PD‐1 after acute cerebrovascular injury. A recent study has found that intraperitoneal injection of soluble PD‐L1 (sPD‐L1) can significantly reduce brain edema and improve overall survival rates [256]. However, this reduction is not observed in PD‐1 knockout animals, confirming that PD‐1^+^ monocytes are the therapeutic target of sPD‐L1. ScRNA‐seq revealed that sPD‐L1 treatment induced monocytes matured into a nonclassical LY6C^lo^, CD43^hi^, PD‐L1^+^ phenotype, suggesting the potential of using sPD‐L1 to activate PD‐1 on peripheral monocytes to limit secondary inflammatory damage.

Endogenous molecules in the brain are anticipated to promote the survival and repair of neurons surrounding the injured site and accelerate stroke recovery. Phospholipase a2 group IIE (PLA2G2E) is considered one of the therapeutic targets for IS for metabolizing phosphatidylserine significantly exposed on the dead cell surface in necrotic brain tissues [294]. Nakamura *et al.* discovered that PLA2G2E secreted by neurons surrounding the infarct induced neuronal peptidyl arginine deiminase 4 (PADI4) expression by generating dihomo‐γ‐linolenic acid (DGLA) [257]. Moreover, AI4 is a global transcriptional regulator in neurons surrounding the infarct [295, 296]. ScRNA‐seq and epigenetic analyses indicated that neuronal PADI4 exhibited the potential to activate genes associated with the recovery process following IS through histone citrullination. Researchers found that among various DGLA metabolites, 15‐hydroxy‐eicosatrienoic acid induced PADI4 in peri‐infarct‐surviving neurons enhances functional recovery after IS. Furthermore, another study identified a prominent Treg cell cluster among brain‐infiltrating immune cells during the chronic phase after IS in mice [297]. Treg cell‐derived OPN was found to enhance microglial reparative activity through integrin receptors, thereby promoting oligodendrogenesis and WM regeneration. Increasing the number of Treg cells by administering IL‐2:IL‐2 antibody complexes significantly improved WM integrity and long‐term functional recovery after IS. As a molecule closely related to vascular endothelial growth factor (VEGF) signaling [298], X‐box binding protein 1 (XBP1) has been found to be a key regulator of a novel endothelial cell subtype associated with angiogenesis (termed healing EC) post IS [258]. Analysis of two brain scRNA‐seq datasets of MCAO mice indicated that healing EC highly expressed pan‐endothelial cell markers. The proangiogenesis genes consisted of high stemness and could differentiate into other endothelial cell subtypes. Additionally, healing EC may regulate angiogenesis by secreting signaling molecules (such as XBP1). Comparative imaging based on synchrotron radiation propagation further demonstrated that XBP1 promoted angiogenesis and restored normal vascular conformation, particularly in the striatum and prefrontal cortex under MCAO condition. A recent study reported two distinct pericyte subtypes (*Ng2^+^* and *Tbx18^+^*), possessing different potentials following IS [259]. Besides, *Ng2* ^+^ pericytes exhibit superior neural reprogramming potential in generating new neurons, while *Tbx18^+^* pericytes display dominant multipotency after IS, capable of producing endothelial cells, fibroblasts, and microglia. In neural reprogramming culture models of *Ng2^+^* pericytes from mice and humans, researchers discovered the role of the energy sensor adenosine monophosphate‐activated protein kinase (AMPK) in modulating the reprogramming/differentiation process. AMPK modulators can regulate the phosphorylation status of Ser 436 on cAMP‐response element binding protein (CREB)‐binding protein, coordinate the acetylation shift between SOX2 and histone H2B, and regulate the nuclear‐cytoplasmic shift of SOX2 during the reprogramming/differentiation process of microsphere cells. This helps promote the conversion of pericytes into functional neurons after IS.

#### 6.1.2 Hemorrhagic Stroke

Post SAH, the meningeal lymphatic system within the dura mater transports large molecules from the brain parenchyma outward and conveys cerebrospinal fluid to the surrounding cervical lymph nodes, contributing to the clearance of pathological substances [299, 300]. Previous studies have demonstrated that meningeal lymphatic vessels (mLVs) played a key role in the removal of intracranial waste [301, 302]. However, there is limited literature about the cellular structure and potential gene regulatory characteristics of mLVs post SAH. Recently, changes in the cellular, molecular, and spatial patterns of mLVs post‐SAH were revealed with stLearn [260, 303], and the THBS1–CD47 L‐R pair was predicted to promote mLVs injury. In vivo experiments demonstrated the detrimental effects of THBS1 and CD47 following SAH. GSEA indicated that the apoptosis pathway was significantly activated 24 h postinjury, with experimental results confirming that the THBS1–CD47 L‐R promotes mLVs apoptosis through the STAT3/Bcl‐2 signaling pathway. In addition, researchers revealed that the expression of *S100a6* in the meninges significantly increased 24 h after injury, while the expression of *S100a6* and THBS1 displayed a linear relationship. Prognostic analysis suggested that higher expression of *S100a6* was related to worse prognosis, suggesting that *S100a6* may be a novel biomarker for meningeal lymphatic injury.

Recently, results of cell‐to‐cell interaction analysis and ingenuity pathway analysis canonical pathway analysis based on ST and scRNA‐seq indicated that insulin like growth factor 1 (IGF1)–IGF1 receptor ligand receptor pair may mediate the interaction of microglia with astrocytes [261]. Specifically, IGF1 derived from microglia promotes astrocyte scar formation through the activation of the mTOR signaling pathway in the early stages of ICH. This process helps establish a favorable microenvironment for remyelination and BBB repair, providing a potential therapeutic target for the treatment of early ICH. Due to the ability to penetrate BBB, Celastrol, the primary active ingredient of traditional Chinese medicine *Tripterygium wilfordii* [304, 305, 306], has been found to play a therapeutic role in CNS diseases [307, 308]. In the secondary brain injury following ICH, with the employment of snRNA‐seq (10x Genomics), Celastrol was reported to influence cyclic adenosine monophosphate/cAMP‐activated exchange protein‐1 (EPAC‐1) signaling pathways [262, 309], thereby improving mitochondrial dysfunction in neurons and exerting neuroprotective effects in mice after ICH. Damage or dysfunction within the CNS can lead to persistent chronic pain, with central poststroke pain (CPSP) being a typical manifestation. Thalamic hemorrhage, a specific form of cerebral hemorrhage, is particularly prone to triggering CPSP. Utilizing snRNA‐seq, researchers have conducted a comprehensive analysis of the alterations in neuronal gene expression within a model of CPSP induced by ICH [263]. Through the analysis of DEGs between control and model groups and the construction of a miRNA–mRNA network, *calcium voltage‐gated channel subunit alpha1 C* (*Cacna1c*) has been predicted as a pivotal therapeutic target for CPSP.

### 6.2 Alzheimer's Disease

Selective removal of *Apoe4* from cells can reduce the pathological burden associated with AD. For instance, in 2021, a study employed snRNA‐seq to investigate the cell‐specific role of APOE in neurodegeneration revealed that the removal of astrocytic *Apoe4* reduced tau‐induced synaptic loss and microglial phagocytosis of synaptic components [264]. Moreover, the selective genetic removal of neuronal *Apoe4* can ameliorate the progressive cellular and tissue changes that occur in *Apoe4*‐driven tau pathology models [265]. snRNA‐seq revealed that the removal of neuronal *Apoe4* significantly reduced the subpopulations of neurons, oligodendrocytes, astrocytes, and microglia associated with neurodegenerative diseases, which correlate with the severity of tau pathology, neurodegeneration, and myelin deficits. Notably, a current study has demonstrated that the depletion of microglia can alleviate the deposition of Aβ and p‐tau induced by *Apoe4* neurons [266]. These findings underscore the significance of neuron–microglia interactions in the pathogenesis of AD.

Microglial receptor triggering receptor expressed on myeloid cells‐2 (TREM2) is essential for microglia to respond to neurodegeneration [310]. Combined with immunofluorescence technology, scRNA‐seq analysis determined that IL‐3, as a key medium of astrocyte–microglia communication, can reprogram microglia to improve AD pathology [267]. IL‐3 in the brain is primarily derived from astrocytes, and its receptor IL‐3Rα is located on microglia and regulated by TREM2. Notably, scRNA‐seq was used to assess the effectiveness of TREM2‐activated antibodies. A novel therapeutic candidate, termed antibody transport vehicle (ATV), was characterized as a high‐affinity human TREM2 antibody engineered with a monovalent transferrin receptor binding site in the Fc domain to enable active transport into the CNS [268]. Furthermore, scRNA‐seq and morphometry revealed that ATV:TREM2 shifts microglia to metabolically responsive states, which are distinct from those induced by amyloid pathology. Additionally, ATV:TREM2 promotes microglia proliferation through mTOR and PLCG2 and is biologically distinct from other innate immune stimuli. These findings provide new insights into the mechanisms by which the therapeutic candidate ATV:TREM2 increases microglial functions. Similarly, as a drug inducing passive Aβ immunization, lecanemab treatment has been reported to modulate specific responses in microglia [269]. Through the integration of scRNA‐seq and ST, up‐regulated expression of genes associated with Aβ clearance has been observed in microglia within the brains of lecanemab‐treated patients, such as *Trem2*, and the degree of microglial activation and the efficiency of Aβ clearance have been found to vary across different brain regions. A loss‐of‐function variant of *Trem2* is associated with an increased risk of AD [311], suggesting that activating this innate immune receptor could serve as a potential therapeutic strategy. Patients with AD carrying the *Trem2* R47H variant exhibited defects in microglial transcriptional activation to a certain extent [312], while R47H mice exhibited an increased expression of genes involved in oxidative stress and lipid metabolism in the brain, and decreased expression of genes involved in autophagy, growth factor signaling, and neural connectivity. ST analysis of AD model mice combined with R47H demonstrated that the presence of this variant impeded microglia response to plaque early in the disease and exacerbated peripheral dystrophic neurites. Moreover, the R47H microglia‐induced transcriptional changes include the AKT signaling activation, which is among the TREM2 downstream pathways, and pharmacological inhibition of AKT is a potential therapeutic strategy [270]. In R47H heterozygous tauopathy mice, treatment with MK‐2206 eliminated tauopathy‐dependent microglial subclusters and rescued tauopathy‐induced synaptic loss by inhibiting AKT signaling.

Aβ around cerebral blood vessels is considered both a cause and a consequence of BBB damage, which ultimately serves as an early biomarker for cognitive dysfunction [313]. To reveal the molecular changes underlying BBB dysfunction in AD, snRNA‐seq analysis of 24 cases of AD and control brains was performed, focusing on vascular and astrocyte clusters as the main cell types of the BBB gliovascular unit [271]. The prediction of ligand–receptor interactions indicated an inverse relationship between pericytic SMAD family member 3 (SMAD3) and astrocytic VEGFA. Whole‐brain correlation analysis between blood SMAD3 levels and cerebral Aβ deposition indicated an association between higher blood SMAD3 levels and less brain Aβ. Moreover, VEGFA downregulation may upregulate pericytic SMAD3, ultimately affecting the function of BBB, confirming in human iPSC and zebrafish model experiments.

SPP1, which was increased by perivascular macrophages in patients with AD, was reported to be essential for microglia activation for the phagocytosis of synapses [272]. ScRNA‐seq and computational ligand‐target predictions (NicheNet28) [314] analysis in microglia revealed that perivascular SPP1 induced microglial phagocytosis in the AD mice hippocampus, and loss of SPP1 expression prevented synaptic loss, suggesting a functional role of SPP1 in the crosstalk between perivascular cells and microglia in AD. To study neuronal maturation, cell type‐specific vulnerability or dysregulation in a more physiological in vivo context, iPSC‐derived neural precursor cells carrying the fAD‐associated APP V717I mutation and their isogenic controls were injected into the striatum and cortex of 2‐month‐old, immunocompromised mice, followed by histological and transcriptional analyses on grafted cells [273]. snRNA‐seq analysis identified significantly altered transcriptomic signatures in APP V717I iPSC‐derived neurons, including upregulation of the transmembrane protein leucine rich repeat and ig domain containing 2 (LINGO2). LINGO2 knockdown exerted beneficial effects on APP V717I neurons by affecting cellular signaling pathways, including the extracellular regulated protein kinases (ERK)/MAPK signaling pathway, hence, rescuing neurite outgrowth defects and reversing transcriptional changes associated with synaptic dysfunction.

Adaptive immune cells are increasingly being utilized in the pathogenesis of AD [315, 316]. Previous studies have revealed that higher numbers of T cells are observed in the brains of AD mice models [317, 318]. To investigate the molecular mechanisms involved in the infiltration of peripheral immune cells into the neuropathological environment of AD, Jorfi et al. [274] described a 3D model of the human neuroimmune axis, including stem cell‐derived neurons, astrocytes and microglia, and human peripheral immune cells. Using this model, the researchers found that the number of CD8^+^ T cells was increased in patients with AD and AD‐like transgenic models. The coexistence of CD8^+^ T cells and microglia exerted synergistic effects, which aggravated the neuronal and glial cell damage. ScRNA‐seq analysis revealed that T cell infiltration into AD cultures induced IFN‐γ and neuroinflammatory pathways in glial cells. The C‐X‐C motif chemokine ligand 10 (CXCL10) and its receptor CXCR3 are critical in this process. CXCL10 induces T cell chemotaxis by binding to CXCR3, a receptor expressed by CD8^+^ T cells. Inhibiting CXCL10 binding to CXCR3 with anti‐CXCR3 neutralizing antibody (MAB160) attenuated CD8^+^ T cell infiltration and neurodegeneration.

### 6.3 Parkinson's Disease

Transplantation of cells from fetal brain tissue or from human embryonic stem cells (hESCs) or human pluripotent stem cells (hPSCs), which can generate DaNs, is a critical treatment of PD. A recent study supports the use of stem‐cell‐based models at a single resolution to study human ventral midbrain (VM) development for disease modeling and therapeutics [319]. Researchers demonstrated that cell types derived from hPSCs were similar to those derived from fetal VM development without any aberrant cell types. However, the temporal appearance of different cell types corresponded to the order of progression observed in human fetal VM. Particularly, the molecular profile of DA neurons derived from hPSCs closely matched that of VM‐derived DA neurons. However, the heterogeneity of hPSC‐derived donor cells and the low yield of mDA neurons after transplantation hinder its broad clinical application. ScRNA‐seq characterizes the single‐cell molecular landscape of DaNs and non‐mDA cells during DaN differentiation, which holds promise as a therapeutic strategy by eliminating non‐mDA cells and facilitating the enrichment of DaNs.

Choroid plexus epithelial cells (CPECs) are the main non‐DA cell type, and CD99 is a candidate surface marker for the purification of DA progenitors, which can be used to eliminate CPECs and improve the purity of DA progenitors via fluorescence‐activated cell sorting (FACS) isolation [24]. Moreover, as it is highly expressed in EN1^+^ DA progenitors, leucine‐rich glioma‐inactivated 1 can partially induce cell fate transition toward DA identities. Calsyntenin 2 and protein tyrosine phosphatase receptor type O are identified as surface markers of mDA progenitors, facilitating high enrichment of mDA neurons. mDA neurons are stably enriched in marker‐sorted grafts, while non‐DA populations are consistently depleted [275]. Another study disclosed important developmental factors for the differentiation of hESCs into mDA neurons [276]. ScRNA‐seq revealed high‐quality hESC‐derived cell types that included mDA neurons, resembling the human midbrain with their new protocol. Laminin‐511, along with dual activation of canonical and noncanonical wingless‐related integration site (WNT) pathways followed by GSK3β inhibition and fibroblast growth factor 8b, improved midbrain patterning. Additionally, activation of liver X receptors and inhibition of fibroblast growth factor signaling further enhanced neurogenesis and differentiation.

Typically, stem cells are differentiated and mature under traditional monolayer (2D) culture conditions. However, it may be inadequate for the maintenance of TH neurons. Some cells in 2D culture undergo incomplete differentiation or insufficient patterning toward a VM regional fate. ScRNA‐seq has identified the advantage of 3D organoid‐like cultures in generating and maintaining mature DA neurons [320]. Researchers performed extensive scRNA‐seq analysis to decode VM development in human embryos from onset to peak DA genesis. Consequently, they established a 3D organoid‐like culture system, which profiles functionally mature DA neurons in 3D cultures derived from the human fetal VM. Conversely, an adeno‐associated virus (AAV)‐based strategy carrying intein–split–dCas9 in combination with activators (AAV–dCAS), a novel intervention strategy beyond the restoration of dopamine level, may provide a substitute route for clinical therapies of PD [277]. Single‐cell transcriptome analysis confirmed that AAV–dCAS successfully reprogrammed striatal astrocytes into induced GABAergic neurons. These GABAergic neurons functionally integrate into striatal circuits, alleviating voluntary motor behavior aspect.

Recent research has shifted from cell replacement to multitargeted therapies like immune regulation and paracrine signaling [321, 322]. Exploring immunomodulation in PD can help identify new therapeutic targets and strategies to mitigate or reverse neurodegenerative changes [323]. Persistent inflammatory responses of microglia and astrocytes significantly contribute to PD progression [324]. Intraspinal transplantation of hypoxia‐preconditioned olfactory mucosal mesenchymal stem cells (hOM–MSCs) improves neurologic function recovery and modulates neuroinflammation in PD patients. hOM–MSCs maintain microglia autophagy homeostasis and promote their transformation from M1 to M2 phenotype via TGF‐β1‐activated activin receptor‐like kinase (ALK)–phosphoinositide 3‐kinas (PI3K)–AKT signaling in microglia [278]. Additionally, a recent study used an E. coli MG1655 strain to produce melanin‐containing exosomes, reducing astrocytic synapse engulfment by activating the prosaposin (PSAP)–GPR37L1 pathway and protecting DaNs [279]. Moreover, scRNA‐seq data from PD patients shows that elevated *Cth* expression correlates with neurotoxic astrocyte reactivity. Researchers found cystathionine γ‐lyase (CSE) induces astrocytes' neurotoxic transition via the CSE–YAP–forkhead box d3 (FOXD3) axis in PD, suggesting a potential therapeutic target [280]. Furthermore, Janus tyrosine kinase (JAK)/STAT pathway suppression shows clinical potential in inhibiting innate and acquired immune cell activation and infiltration. Interestingly, scRNA‐seq reveals reduced microglia cluster cell numbers under JAK/STAT suppression, with no transcriptional profile changes [281]. Collectively, these findings suggest that scRNA‐seq can uncover the potential of cell replacement and identify potential targets from immune regulation for PD treatment.

### 6.4 Glioblastoma

TAMs are essential in supporting tumor progression and inducing immunosuppression. Emerging evidence demonstrates that the infiltration of immunosuppressive TAMs likely resulted in GBM being unresponsive to immunotherapy [325, 326]. These findings indicate TAM as a potential therapeutic target for GBM. The following section summarizes current TAM‐targeted therapeutic strategies in GBM.

The first strategy is to inhibit TAM infiltration by targeting the axes between their receptors and ligands. Using the slit guidance ligand 2 (SLIT2)‐trapping protein Robo1Fc effectively inhibits macrophage infiltration and tumor growth in GBM mouse models, significantly improving the antitumor effects by its combination with anti‐PD‐1 and anti‐4‐1BB therapies [282]. Mechanistically, SLIT2 promotes microglia/macrophage chemotaxis and tumor‐supportive polarization via roundabout guidance receptor 1/2 (ROBO1/2)‐mediated PI3Kγ activation in macrophages. Consequently, inhibiting PI3Kγ prevents TAM accumulation in the GBM TME and elevates the antitumor efficacy of temozolomide [283]. Moreover, the properties of driving GBM immunosuppression of clock circadian regulator (CLOCK) have been reported in recent studies [284, 285]. CLOCK and its heterodimeric partner BMAL1 enhance microglial infiltration into the GBM TME through olfactomedin‐like 3 and legumain upregulation. Inhibiting CLOCK–olfactomedin‐like 3–HIF1α–legumain–CD162 axis impairs microglial infiltration and increases CD8^+^ T‐cell infiltration, activation, and cytotoxicity, and synergizes with anti‐PD‐1 therapy. Additionally, studies have revealed additional targetable chemokine‐receptor pairs. For instance, bone morphogenetic protein‐4 (BMP4) promotes astrocytes and suppresses oligodendrocyte differentiation in GSCs [327]. Single‐cell transcriptome profiling indicates that oligodendrocyte transcription factor 1/2 (OLIG1/2) expression can predict the treatment efficacy after BMP4 treatment and upregulation of *Rpl27a* and *Rps27*. This marks the early efficacy of treatment with BMP4, which contributes to precision medicine in that patients could be stratified alternatively in novel clinical trial. Moreover, poor antigen presentation is a major obstacle to immunotherapy of GBM. Single‐cell transcriptomic data suggest that the MET–STAT4–PD‐L1 axis may enforce glioma immune evasion while activation of the MET pathway induces upregulation of PD‐L1, revealing a potential treatment using a combination of MET and PD‐L1 inhibitors [213]. However, targeting the axes of TAM infiltration may not be completely curative. For instance, scRNA‐seq data analysis disclose that GBM tumors from C‐C motif chemokine receptor 2 (CCR2)‐knockout mice harbor a reduced TAM subpopulation with macrophage signatures (*Tgfbi*,  *Clec12a*, and *Fxyd5*) [328]. Conversely, an increased subpopulation with microglia signatures (*Sall1*,  *Tmem119*, and *P2ry12*) indicates that the antitumor effect of CCR2 inhibition can be attenuated by increased microglia.

The second strategy is to target newly discovered TAM subpopulations. For instance, DCs and PD‐L1^+^ TAM are promising targetable cell populations. Researchers have demonstrated that PD‐L1 blockade is more effective than dual cytotoxic t‐lymphocyte‐associated protein 4/PD‐1 blockade in the S28 model. Targeting PD‐L1 combined with stereotactic radiation suggests effectiveness in prolonged survival of mice with intracerebral SB28 tumors at the cost of increased Tregs [211]. Moreover, targeting CD73‐high macrophages through depletion of CD73 extend the survival of GBM‐bearing mice, and anti‐PD‐1 and anti‐CTLA4 therapies further improve this effect [286]. Moreover, CD73 is identified as mainly derived from GBM cells [329]. Therefore, using anti‐CD73 antibodies and CD73 small‐molecule inhibitors plays a dual role in targeting both CD73‐high macrophages and tumor cells, which has gained promising results in preclinical and early clinical trials [330, 331]. CD163^+^HHMOX1^+^ microglia is another newly discovered microglia subpopulation in GBM. It releases IL‐10, which upregulates granzyme B in T cells via the JAK/STAT pathway. JAK1/2‐inhibitor Ruxolitinib boosted T cell activation by reducing immunosuppressive programs in myeloid cells, with patients with GBM clinically living for 2 years after Ruxolitinib treatment [287]. The newly identified GPNMB‐high macrophage plays a crucial role in PMT transition through immune cell‐tumor interplay. Moreover, GPNMB‐high macrophage subpopulations can ineffectively retain T cells from activating by DCs by competing with DCs. Therefore, targeting the GPNMB‐high macrophage subset may benefit in controlling GBM plasticity and facilitate T cell‐based immunotherapy [212]. Consequently, these findings suggest that these TAM subpopulations are immunosuppressive, which are potential therapeutic targets. However, TAMs are heterogeneous, and immune‐activating subpopulations still exist. Newly identified CD169^+^ macrophage subpopulations contribute to antitumor immunity against GBM, facilitating phagocytosis through ligand binding on apoptotic tumor cells and boosting tumor‐specific T‐cell responses through CD169. IFN‐γ from NK cells plays an essential role in the infiltration of CD169^+^ macrophage, and targeting this pathway could promote a proinflammatory TME with glioma [219]. Therefore, these TAM subpopulations are promising targets for the treatment of patients with GBM.

## 7 Conclusion and Perspectives

The integration of single‐cell and spatial multiomics technologies has revolutionized our understanding of CNS diseases, offering unprecedented insights into cellular heterogeneity, microenvironmental dynamics, and molecular cascades driving neurodegeneration, neuroinflammation, and injury. By correlating transcriptional states, epigenetic landscapes, protein interactions, and metabolic fluxes within their native spatial contexts, these approaches have identified novel biomarker, uncovered therapeutic target, and refined patient stratification frameworks.

However, critical challenges and opportunities for innovation remain. For example, (1) *Dynamic and temporal gaps*. Most technologies provide static snapshots, failing to capture real‐time metabolic fluxes or epigenetic remodeling during disease progression. Neurodegenerative diseases like AD require longitudinal multiomics to dissect Aβ plaque evolution and tau propagation. (2) *Data integration complexity*. Crossmodal alignment like transcriptome‐metabolome‐proteome is hindered by technical noise, batch effects, and missing data. Integrating scRNA‐seq with spatial proteomics demands robust normalization algorithms to resolve cell‐type‐specific signaling gradients. (3) *Clinical translation barriers*. High costs, tissue availability, and computational expertise limit widespread adoption in clinical settings.

Several directions hold great promise. (1) *Subcellular and live‐cell multiomics sequencing*. Multiomics in situ pairwise sequencing (MiP‐seq), a method for simultaneous detection of DNA, RNA, proteins, and biomolecules at subcellular resolution [72]. MiP‐seq can be combined with in vivo calcium imaging and Raman imaging to generate spatial multiomics maps of mouse brain tissue and correlate gene expression with neuronal activity and cellular biochemical fingerprints. (2) *Integration of multiomics data*. Advanced computational capabilities and more sophisticated algorithms are required to deeply integrate single‐cell and spatial multiomics data, enabling the construction of comprehensive biomolecular networks and regulatory models. For instance, a recently developed tool, genetically informed spatial mapping of cells for complex traits (gsMap), enables the spatial localization of trait‐relevant cells by integrating high‐resolution ST data with summary statistics from GWAS [332]. GsMap employs a graph neural network to integrate gene expression profiles, spatial coordinates, and cell type prior information. Based on the cosine similarity of the learned embedding vectors, it identifies homogeneous spots for each spot to form a microdomain, then calculates the gene specificity score (GSS) for each spot by comparing the ranked gene expression within the identified microdomain against the ST dataset. Subsequently, the spot‐level GSS is mapped to single nucleotide polymorphisms (SNPs). Stratified linkage disequilibrium score regression is utilized to evaluate the enrichment of trait heritability for the SNP–GSS annotations. Finally, the Cauchy combination test is applied to integrate the *p* values of spots within a spatial region. Researchers utilized gsMap to map human complex traits to brain spatial ST dataset from mouse and macaque, revealing that the cerebral cortex exhibited the strongest associations with the majority of the investigated traits. Furthermore, they spatially localized a specific class of glutamatergic neurons exhibiting a high relevant with depression. (3) *Full‐length ST*. Full‐length transcriptomics enables the acquisition of complete sequences of individual transcript molecules, thereby allowing accurately analyzing the transcript isomers. Current mainstream platforms can achieve high throughput [333], high sensitivity [334], and high accuracy [335] while providing long reading. Applying advancements in full‐length transcriptomics to ST holds the theoretical potential to yield spatially resolved full‐length sequence information. For example, spatial isoform transcriptomics (SiT), which integrates the Visium platform with Oxford Nanopore Sequencing technology, revealed regional isoform switching of the Plp1 across different layers of the olfactory bulb and determined the specific cell types expressing each isoform by leveraging single‐cell datasets [336]. However, SiT's spatial resolution is constrained by the inherent limitations of the Visium platform, and the assignment efficiency of Nanopore sequencing reads to spatial locations is currently lower than that achievable with Illumina sequencing. Consequently, the development of more mature full‐length ST technologies represents a highly promising avenue for development. Progress in such technologies will empower researchers to gain deeper insights into the mechanistic roles of posttranscriptional modifications in CNS diseases. (4) *Advance of open‐source repositories*. Establish global consortia to standardize data formats and share multiomics datasets. Currently available single‐cell multiomics databases include Single Cell Portal [337], Human Cell Atlas [338], UCSC Cell Browser [339], and Tabula Sapiens [340]; spatial multiomics databases encompass HuBMAP [341] and SpatialOMx (Table 3).

**TABLE 3:** Open‐source database for single‐cell and spatial multiomics.

Category	Database name	Omics type	Advantage	Website	References
Single‐cell multiomics	Single Cell Portal	Transcriptomics, proteomics, genomics	Cross‐omics integration analysis support multiomics data superposition display Contain datasets on cancer, COVID‐19, neurodegenerative diseases and more	https://singlecell.broadinstitute.org/	[337]
	Human Cell Atlas	Transcriptomics, proteomics, genomics, immunomics	Contain all human organ atlas, standardized processes and data anonymous	https://data.humancellatlas.org/	[338]
	UCSC Cell Browser	Transcriptomics, proteomics, genomics	Enable uploading custom single‐cell datasets and generate interactive browsers, multiomics visualizations, and cross‐species compatibility	https://cells.ucsc.edu/	[339]
	Tabula Sapiens	Transcriptomics, proteomics, immunomics	Support organ cell interactions analysis with built‐in CellPhoneDB tools. Allow directly downloading raw data matrix or the results of the analysis	https://tabula‐sapiens.sf.czbiohub.org/	[340]
Spatial multiomics	Human BioMolecular Atlas Program	Transcriptomics, proteomics, metabolomics	Integrate tissue slide data to build 3D molecular maps, providing tools to map spatial omics to single‐cell data	https://portal.hubmapconsortium.org/	[341]
	SpatialOMx	Transcriptomics, proteomics, metabolomics	High sensitivity detection of metabolites and multiple process platform integrating Clinical transformation: support the heterogeneity of tumor metabolic analysis and drug response prediction	https://www.bruker.com/en/applications/academia‐life‐science/imaging/maldi‐imaging/SpatialOMx.html	/

ScRNA‐seq and ST technologies have strengthened the study of CNS diseases with unprecedented power by combining multiomics sequencing, including proteomics, metabolomics, and epigenetics, to decipher the cellular and molecular landscape and even spatially parse these data. We believe that scRNA‐seq and ST technologies will enable the characterization of diseased brains at cell type and even subcellular resolution and provide valuable information for designing new therapies for patients with CNS disorders. Moreover, the emerging single‐cell multiomics technologies will lead to the discovery of new vistas in studying disease mechanisms, precision treatment, and clinical applications.

## Author Contributions

Mingkai Xia and Quan Liu wrote the manuscript. Zhigang Mei and Jinwen Ge conceived and supervised this work and revised the manuscript. Wenli Zhang provided some positive suggestions and amended the manuscript. All authors approved the final version.

## Ethics Statement

The authors have nothing to report.

## Conflicts of Interest

The authors declare no conflicts of interest.

## Data Availability

The authors have nothing to report.

## References

[1] V. L. Feigin , T. Vos , E. Nichols , et al., “The Global Burden of Neurological Disorders: Translating Evidence Into Policy, ” Lancet Neurology 19, no. 3 (2020): 255‐265. 31813850 10.1016/S1474-4422(19)30411-9 PMC9945815

[2] E. Z. Macosko , A. Basu , R. Satija , et al., “Highly Parallel Genome‐wide Expression Profiling of Individual Cells Using Nanoliter Droplets, ” Cell 161, no. 5 (2015): 1202‐1214. 26000488 10.1016/j.cell.2015.05.002 PMC4481139

[3] S. G. Rodriques , L. M. Chen , S. Liu , et al., “RNA Timestamps Identify the Age of Single Molecules in RNA Sequencing, ” Nature Biotechnology 39, no. 3 (2021): 320‐325. 10.1038/s41587-020-0704-z PMC7956158 33077959

[4] P. L. Ståhl , F. Salmén , S. Vickovic , et al., “Visualization and Analysis of Gene Expression in Tissue Sections by Spatial Transcriptomics, ” Science 353, no. 6294 (2016): 78‐82. 27365449 10.1126/science.aaf2403

[5] H. Mathys , C. A. Boix , L. A. Akay , et al., “Single‐cell Multiregion Dissection of Alzheimer's Disease, ” Nature 632, no. 8026 (2024): 858‐868. 39048816 10.1038/s41586-024-07606-7 PMC11338834

[6] T. Kamath , A. Abdulraouf , S. J. Burris , et al., “Single‐cell Genomic Profiling of human Dopamine Neurons Identifies a Population That Selectively Degenerates in Parkinson's Disease, ” Nature Neuroscience 25, no. 5 (2022): 588‐595. 35513515 10.1038/s41593-022-01061-1 PMC9076534

[7] S. Ferri‐Borgogno , Y. Zhu , J. Sheng , et al., “Spatial Transcriptomics Depict Ligand‐Receptor Cross‐talk Heterogeneity at the Tumor‐Stroma Interface in Long‐Term Ovarian Cancer Survivors, ” Cancer Research 83, no. 9 (2023): 1503‐1516. 36787106 10.1158/0008-5472.CAN-22-1821 PMC10159916

[8] H. Zeng , J. Huang , H. Zhou , et al., “Integrative in Situ Mapping of Single‐cell Transcriptional States and Tissue Histopathology in a Mouse Model of Alzheimer's Disease, ” Nature Neuroscience 26, no. 3 (2023): 430‐446. 36732642 10.1038/s41593-022-01251-x PMC11332722

[9] R. Argelaguet , A. S. E. Cuomo , O. Stegle , J. C. Marioni , “Computational Principles and Challenges in Single‐cell Data Integration, ” Nature Biotechnology 39, no. 10 (2021): 1202‐1215. 10.1038/s41587-021-00895-7 33941931

[10] Y. Hasin , M. Seldin , A. Lusis , “Multi‐omics Approaches to Disease, ” Genome Biology 18, no. 1 (2017): 83. 28476144 10.1186/s13059-017-1215-1 PMC5418815

[11] S. Dujardin , C. Commins , A. Lathuiliere , et al., “Tau Molecular Diversity Contributes to Clinical Heterogeneity in Alzheimer's Disease, ” Nature Medicine 26, no. 8 (2020): 1256‐1263. 10.1038/s41591-020-0938-9 PMC7603860 32572268

[12] D. Lähnemann , J. Köster , E. Szczurek , et al., “Eleven Grand Challenges in Single‐cell Data Science, ” Genome Biology 21, no. 1 (2020): 31. 32033589 10.1186/s13059-020-1926-6 PMC7007675

[13] L. Moses , L. Pachter , “Museum of Spatial Transcriptomics, ” Nature Methods 19, no. 5 (2022): 534‐546. 35273392 10.1038/s41592-022-01409-2

[14] T. R. Hammond , C. Dufort , L. Dissing‐Olesen , et al., “Single‐Cell RNA Sequencing of Microglia throughout the Mouse Lifespan and in the Injured Brain Reveals Complex Cell‐State Changes, ” Immunity 50, no. 1 (2019): 253‐271. e6. 30471926 10.1016/j.immuni.2018.11.004 PMC6655561

[15] P. J. Magistretti , I. Allaman , “Lactate in the Brain: From Metabolic End‐product to Signalling Molecule, ” Nature Reviews Neuroscience 19, no. 4 (2018): 235‐249. 29515192 10.1038/nrn.2018.19

[16] B. Diaz‐Castro , M. R. Gangwani , X. Yu , G. Coppola , B. S. Khakh , “Astrocyte Molecular Signatures in Huntington's disease, ” Science Translational Medicine 11, no. 514 (2019): eaaw8546. 31619545 10.1126/scitranslmed.aaw8546

[17] D. Gosselin , D. Skola , N. G. Coufal , et al., “An Environment‐dependent Transcriptional Network Specifies human Microglia Identity, ” Science 356, no. 6344 (2017): eaal3222. 28546318 10.1126/science.aal3222 PMC5858585

[18] Y. Hao , S. Hao , E. Andersen‐Nissen , et al., “Integrated Analysis of Multimodal Single‐cell Data, ” Cell 184, no. 13 (2021): 3573‐3587. e29. 34062119 10.1016/j.cell.2021.04.048 PMC8238499

[19] R. W. Yeo , O. Y. Zhou , B. L. Zhong , et al., “Chromatin Accessibility Dynamics of Neurogenic Niche Cells Reveal Defects in Neural Stem Cell Adhesion and Migration During Aging, ” Nat Aging 3, no. 7 (2023): 866‐893. 37443352 10.1038/s43587-023-00449-3 PMC10353944

[20] Y. Pan , W. Cao , Y. Mu , Q. Zhu , “Microfluidics Facilitates the Development of Single‐Cell RNA Sequencing, ” Biosensors (Basel) 12, no. 7 (2022): 450. 35884253 10.3390/bios12070450 PMC9312765

[21] N. Navin , J. Hicks , “Future Medical Applications of Single‐cell Sequencing in Cancer, ” Genome Med 3, no. 5 (2011): 31. 21631906 10.1186/gm247 PMC3219072

[22] A. A. Kolodziejczyk , J. K. Kim , V. Svensson , J. C. Marioni , S. A. Teichmann , “The Technology and Biology of Single‐Cell RNA Sequencing, ” Molecular Cell 58, no. 4 (2015): 610‐620. 26000846 10.1016/j.molcel.2015.04.005

[23] F. W. Townes , R. A. Irizarry , “Quantile Normalization of Single‐cell RNA‐seq Read Counts Without Unique Molecular Identifiers, ” Genome biology 21 (2020): 160. 32620142 10.1186/s13059-020-02078-0 PMC7333325

[24] L. Liang , Y. Tian , L. Feng , et al., “Single‐cell Transcriptomics Reveals the Cell Fate Transitions of human Dopaminergic Progenitors Derived From hESCs, ” Stem Cell Res Ther 13 (2022): 412. 35964138 10.1186/s13287-022-03104-7 PMC9375405

[25] S. Aibar , C. B. González‐Blas , T. Moerman , et al., “SCENIC: Single‐cell Regulatory Network Inference and Clustering, ” Nature Methods 14, no. 11 (2017): 1083‐1086. 28991892 10.1038/nmeth.4463 PMC5937676

[26] J. Baran‐Gale , T. Chandra , K. Kirschner , “Experimental Design for Single‐cell RNA Sequencing, ” Brief Funct Genomics 17, no. 4 (2017): 233‐239. 10.1093/bfgp/elx035 PMC6063265 29126257

[27] Fernandes H. J. R. , N. Patikas , S. Foskolou , et al., “Single‐Cell Transcriptomics of Parkinson's Disease Human in Vitro Models Reveals Dopamine Neuron‐Specific Stress Responses, ” Cell Reports 33, no. 2 (2020): 108263. 33053338 10.1016/j.celrep.2020.108263

[28] X. Wang , Y. He , Q. Zhang , X. Ren , Z. Zhang , “Direct Comparative Analyses of 10X Genomics Chromium and Smart‐seq2, ” Genomics, Proteomics & Bioinformatics 19, no. 2 (2021): 253‐266. 10.1016/j.gpb.2020.02.005 PMC8602399 33662621

[29] C. Ziegenhain , B. Vieth , S. Parekh , et al., “Comparative Analysis of Single‐Cell RNA Sequencing Methods, ” Molecular Cell 65, no. 4 (2017): 631‐643. e4. 28212749 10.1016/j.molcel.2017.01.023

[30] B. B. Liau , C. Sievers , L. K. Donohue , et al., “Adaptive Chromatin Remodeling Drives Glioblastoma Stem Cell Plasticity and Drug Tolerance, ” Cell Stem Cell 20, no. 2 (2017): 233‐246. e7. 27989769 10.1016/j.stem.2016.11.003 PMC5291795

[31] R. G. W. Verhaak , K. A. Hoadley , E. Purdom , et al., “An Integrated Genomic Analysis Identifies Clinically Relevant Subtypes of Glioblastoma Characterized by Abnormalities in PDGFRA, IDH1, EGFR and NF1, ” Cancer Cell 17, no. 1 (2010): 98. 20129251 10.1016/j.ccr.2009.12.020 PMC2818769

[32] D. B. Mahat , N. D. Tippens , J. D. Martin‐Rufino , “Single‐cell Nascent RNA Sequencing Unveils Coordinated Global Transcription, ” Nature 631, no. 8019 (2024): 216‐223. 38839954 10.1038/s41586-024-07517-7 PMC11222150

[33] Y. Li , Z. Huang , L. Xu , et al., “UDA‐seq: Universal Droplet Microfluidics‐based Combinatorial Indexing for Massive‐scale Multimodal Single‐cell Sequencing, ” Nature Methods 22, no. 6 (2025): 1199‐1212. 39833568 10.1038/s41592-024-02586-y PMC12165859

[34] C. K. Mo , J. Liu , S. Chen , et al., “Tumour Evolution and Microenvironment Interactions in 2D and 3D Space, ” Nature 634, no. 8036 (2024): 1178‐1186. 39478210 10.1038/s41586-024-08087-4 PMC11525187

[35] J. L. Guo , M. Griffin , J. K. Yoon , et al., “Histological Signatures Map Anti‐fibrotic Factors in Mouse and human Lungs, ” Nature 641, no. 8064 (2025): 993‐1004. 40108456 10.1038/s41586-025-08727-3 PMC12105817

[36] L. A. Huuki‐Myers , A. Spangler , N. J. Eagles , et al., “A Data‐driven Single‐cell and Spatial Transcriptomic Map of the human Prefrontal Cortex, ” Science 384, no. 6698 (2024): eadh1938. 38781370 10.1126/science.adh1938 PMC11398705

[37] A. Sampath Kumar , L. Tian , A. Bolondi , et al., “Spatiotemporal Transcriptomic Maps of Whole Mouse Embryos at the Onset of Organogenesis, ” Nature Genetics 55, no. 7 (2023): 1176‐1185. 37414952 10.1038/s41588-023-01435-6 PMC10335937

[38] J. Langlieb , N. S. Sachdev , K. S. Balderrama , et al., “The Molecular Cytoarchitecture of the Adult Mouse Brain, ” Nature 624, no. 7991 (2023): 333‐342. 38092915 10.1038/s41586-023-06818-7 PMC10719111

[39] S. G. Rodriques , R. R. Stickels , A. Goeva , et al., “Slide‐seq: A Scalable Technology for Measuring Genome‐wide Expression at High Spatial Resolution, ” Science 363, no. 6434 (2019): 1463‐1467. 30923225 10.1126/science.aaw1219 PMC6927209

[40] S. Vickovic , G. Eraslan , F. Salmén , et al., “High‐definition Spatial Transcriptomics for in Situ Tissue Profiling, ” Nature Methods 16, no. 10 (2019): 987‐990. 31501547 10.1038/s41592-019-0548-y PMC6765407

[41] A. Enninful , Z. Zhang , D. Klymyshyn , et al., “Integration of Imaging‐based and Sequencing‐based Spatial Omics Mapping on the Same Tissue Section via DBiTplus, ” Res Sq (2024). Published online November 11, 2024. 10.1038/s41592-025-02948-0 PMC13541625 41540123

[42] Y. Liu , M. Yang , Y. Deng , et al., “High‐Spatial‐Resolution Multi‐Omics Sequencing via Deterministic Barcoding in Tissue, ” Cell 183, no. 6 (2020): 1665‐1681. 33188776 10.1016/j.cell.2020.10.026 PMC7736559

[43] Y. Lei , X. Liang , Y. Sun , et al., “Region‐specific Transcriptomic Responses to Obesity and Diabetes in Macaque Hypothalamus, ” Cell metabolism 36, no. 2 (2024): 438‐453. e6. 38325338 10.1016/j.cmet.2024.01.003

[44] Y. Zhang , G. Liu , Q. Zeng , et al., “CCL19‐producing Fibroblasts Promote Tertiary Lymphoid Structure Formation Enhancing Anti‐tumor IgG Response in Colorectal Cancer Liver Metastasis, ” Cancer Cell 42, no. 8 (2024): 1370‐1385. e9. 39137726 10.1016/j.ccell.2024.07.006

[45] A. Chen , S. Liao , M. Cheng , et al., “Spatiotemporal Transcriptomic Atlas of Mouse Organogenesis Using DNA Nanoball‐patterned Arrays, ” Cell 185, no. 10 (2022): 1777‐1792. e21. 35512705 10.1016/j.cell.2022.04.003

[46] Y. Fan , Ž. Andrusivová , Y. Wu , et al., “Expansion Spatial Transcriptomics, ” Nature Methods 20, no. 8 (2023): 1179‐1182. 37349575 10.1038/s41592-023-01911-1 PMC11078125

[47] M. Schott , D. León‐Periñán , E. Splendiani , et al., “Open‐ST: High‐resolution Spatial Transcriptomics in 3D, ” Cell 187, no. 15 (2024): 3953‐3972. e26. 38917789 10.1016/j.cell.2024.05.055

[48] M. Schott , D. León‐Periñán , E. Splendiani , et al., “Protocol for High‐resolution 3D Spatial Transcriptomics Using Open‐ST, ” STAR Protoc 6, no. 1 (2025): 103521. 39708325 10.1016/j.xpro.2024.103521 PMC11731217

[49] S. R. Srivatsan , M. C. Regier , E. Barkan , et al., “Embryo‐scale, Single Cell Spatial Transcriptomics, ” Science 373, no. 6550 (2021): 111‐117. 34210887 10.1126/science.abb9536 PMC9118175

[50] J. Ding , L. Li , Q. Lu , et al., “SpatialCTD: A Large‐Scale Tumor Microenvironment Spatial Transcriptomic Dataset to Evaluate Cell Type Deconvolution for Immuno‐Oncology, ” Journal of computational biology 31, no. 9 (2024): 871‐885. 39117342 10.1089/cmb.2024.0532

[51] Y. Lee , D. Bogdanoff , Y. Wang , et al., “XYZeq: Spatially Resolved Single‐cell RNA Sequencing Reveals Expression Heterogeneity in the Tumor Microenvironment, ” Science Advances 7, no. 17 (2021): eabg4755. 33883145 10.1126/sciadv.abg4755 PMC8059935

[52] J. Yang , Z. Zheng , Y. Jiao , et al., “Spotiphy Enables Single‐cell Spatial Whole Transcriptomics Across an Entire Section, ” Nature Methods 22, no. 4 (2025): 724‐736. Published online March 12, 2025. 40074951 10.1038/s41592-025-02622-5 PMC11978521

[53] S. Mallik , J. Venezian , A. Lobov , et al., “Structural Determinants of co‐translational Protein Complex Assembly, ” Cell 188, no. 3 (2025): 764‐777. e22. 39708808 10.1016/j.cell.2024.11.013

[54] A. M. Femino , F. S. Fay , K. Fogarty , R. H. Singer , “Visualization of Single RNA Transcripts in Situ, ” Science 280, no. 5363 (1998): 585‐590. 9554849 10.1126/science.280.5363.585

[55] T. Lu , M. Wang , W. Zhou , et al., “Decoding Transcriptional Identity in Developing human Sensory Neurons and Organoid Modeling, ” Cell 187, no. 26 (2024): 7374‐7393. e28. 39536745 10.1016/j.cell.2024.10.023

[56] E. Lubeck , A. F. Coskun , T. Zhiyentayev , M. Ahmad , L. Cai , “Single‐cell in Situ RNA Profiling by Sequential Hybridization, ” Nature Methods 11, no. 4 (2014): 360‐361. 24681720 10.1038/nmeth.2892 PMC4085791

[57] A. Sarfatis , Y. Wang , N. Twumasi‐Ankrah , J. R. Moffitt , “Highly Multiplexed Spatial Transcriptomics in Bacteria, ” Science 387, no. 6732 (2025): eadr0932. 39847624 10.1126/science.adr0932 PMC12278067

[58] Z. Yao , C. T. J. van Velthoven , M. Kunst , et al., “A High‐resolution Transcriptomic and Spatial Atlas of Cell Types in the Whole Mouse Brain, ” Nature 624, no. 7991 (2023): 317‐332. 38092916 10.1038/s41586-023-06812-z PMC10719114

[59] K. H. Chen , A. N. Boettiger , J. R. Moffitt , S. Wang , X. Zhuang , “RNA Imaging. Spatially Resolved, Highly Multiplexed RNA Profiling in Single Cells, ” Science 348, no. 6233 (2015): aaa6090. 25858977 10.1126/science.aaa6090 PMC4662681

[60] S. Kanatani , J. C. Kreutzmann , Y. Li , et al., “Whole‐brain Spatial Transcriptional Analysis at Cellular Resolution, ” Science 386, no. 6724 (2024): 907‐915. 39571016 10.1126/science.adn9947

[61] P. Kukanja , C. M. Langseth , L. A. Rubio Rodríguez‐Kirby , et al., “Cellular Architecture of Evolving Neuroinflammatory Lesions and Multiple Sclerosis Pathology, ” Cell 187, no. 8 (2024): 1990‐2009. e19. 38513664 10.1016/j.cell.2024.02.030

[62] R. Ke , M. Mignardi , A. Pacureanu , et al., “In Situ Sequencing for RNA Analysis in Preserved Tissue and Cells, ” Nature Methods 10, no. 9 (2013): 857‐860. 23852452 10.1038/nmeth.2563

[63] H. Q. Nguyen , S. Chattoraj , D. Castillo , et al., “3D mapping and Accelerated Super‐resolution Imaging of the human Genome Using in Situ Sequencing, ” Nature Methods 17, no. 8 (2020): 822‐832. 32719531 10.1038/s41592-020-0890-0 PMC7537785

[64] J. H. Lee , E. R. Daugharthy , J. Scheiman , et al., “Fluorescent in Situ Sequencing (FISSEQ) of RNA for Gene Expression Profiling in Intact Cells and Tissues, ” Nature Protocols 10, no. 3 (2015): 442‐458. 25675209 10.1038/nprot.2014.191 PMC4327781

[65] H. Shi , Y. He , Y. Zhou , et al., “Spatial Atlas of the Mouse central Nervous System at Molecular Resolution, ” Nature 622, no. 7983 (2023): 552‐561. 37758947 10.1038/s41586-023-06569-5 PMC10709140

[66] X. Wang , W. E. Allen , M. A. Wright , et al., “Three‐dimensional Intact‐tissue Sequencing of Single‐cell Transcriptional States, ” Science 361, no. 6400 (2018): eaat5691. 29930089 10.1126/science.aat5691 PMC6339868

[67] X. Chen , Y. C. Sun , G. M. Church , J. H. Lee , A. M. Zador , “Efficient in Situ Barcode Sequencing Using Padlock Probe‐based BaristaSeq, ” Nucleic Acids Res. 46, no. 4 (2018): e22. 29190363 10.1093/nar/gkx1206 PMC5829746

[68] Y. Zhang , J. A. Miller , J. Park , et al., “Reference‐based Cell Type Matching of in Situ Image‐based Spatial Transcriptomics Data on Primary Visual Cortex of Mouse Brain, ” Scientific Reports 13, no. 1 (2023): 9567. 37311768 10.1038/s41598-023-36638-8 PMC10264402

[69] X. Chen , S. Fischer , M. C. P. Rue , et al., “Whole‐cortex in Situ Sequencing Reveals Input‐dependent Area Identity, ” Nature (2024). Published online April 24, 2024. 10.1038/s41586-024-07221-6 PMC12589132 38658747

[70] X. Chen , Y. C. Sun , H. Zhan , et al., “High‐Throughput Mapping of Long‐Range Neuronal Projection Using in Situ Sequencing, ” Cell 179, no. 3 (2019): 772‐786. e19. 31626774 10.1016/j.cell.2019.09.023 PMC7836778

[71] Y. C. Sun , X. Chen , S. Fischer , et al., “Integrating Barcoded Neuroanatomy With Spatial Transcriptional Profiling Enables Identification of Gene Correlates of Projections, ” Nature Neuroscience 24, no. 6 (2021): 873‐885. 33972801 10.1038/s41593-021-00842-4 PMC8178227

[72] X. Wu , W. Xu , L. Deng , et al., “Spatial Multi‐omics at Subcellular Resolution via High‐throughput in Situ Pairwise Sequencing, ” Nat Biomed Eng 8, no. 7 (2024): 872‐889. 38745110 10.1038/s41551-024-01205-7

[73] Q. Li , X. Zhang , R. Ke , “Spatial Transcriptomics for Tumor Heterogeneity Analysis, ” Frontiers in Genetics 13 (2022): 906158. 35899203 10.3389/fgene.2022.906158 PMC9309247

[74] J. R. Moffitt , E. Lundberg , H. Heyn , “The Emerging Landscape of Spatial Profiling Technologies, ” Nature Reviews Genetics 23, no. 12 (2022): 741‐759. 10.1038/s41576-022-00515-3 35859028

[75] C. G. Williams , H. J. Lee , T. Asatsuma , R. Vento‐Tormo , A. Haque , “An Introduction to Spatial Transcriptomics for Biomedical Research, ” Genome Med 14, no. 1 (2022): 68. 35761361 10.1186/s13073-022-01075-1 PMC9238181

[76] A. Lyubimova , S. Itzkovitz , J. P. Junker , Z. P. Fan , X. Wu , A. van Oudenaarden , “Single‐molecule mRNA Detection and Counting in Mammalian Tissue, ” Nature Protocols 8, no. 9 (2013): 1743‐1758. 23949380 10.1038/nprot.2013.109

[77] C. H. L. Eng , M. Lawson , Q. Zhu , et al., “Transcriptome‐scale Super‐resolved Imaging in Tissues by RNA seqFISH, ” Nature 568, no. 7751 (2019): 235‐239. 30911168 10.1038/s41586-019-1049-y PMC6544023

[78] N. Jung , T. K. Kim , “Spatial Transcriptomics in Neuroscience, ” Experimental & Molecular Medicine 55, no. 10 (2023): 2105‐2115. 37779145 10.1038/s12276-023-01093-y PMC10618223

[79] H. S. Kaya‐Okur , S. J. Wu , C. A. Codomo , et al., “CUT&Tag for Efficient Epigenomic Profiling of Small Samples and Single Cells, ” Nature Communications 10, no. 1 (2019): 1930. 10.1038/s41467-019-09982-5 PMC6488672 31036827

[80] J. D. Buenrostro , B. Wu , U. M. Litzenburger , et al., “Single‐cell Chromatin Accessibility Reveals Principles of Regulatory Variation, ” Nature 523, no. 7561 (2015): 486‐490. 26083756 10.1038/nature14590 PMC4685948

[81] T. Lu , C. E. Ang , X. Zhuang , “Spatially Resolved Epigenomic Profiling of Single Cells in Complex Tissues, ” Cell 185, no. 23 (2022): 4448‐4464. e17. 36272405 10.1016/j.cell.2022.09.035 PMC9691621

[82] Y. Deng , M. Bartosovic , P. Kukanja , et al., “Spatial‐CUT&Tag: Spatially Resolved Chromatin Modification Profiling at the Cellular Level, ” Science 375, no. 6581 (2022): 681‐686. 35143307 10.1126/science.abg7216 PMC7612972

[83] P. Ma , S. Duan , W. Ma , et al., “Single‐cell Chromatin Accessibility Landscape Profiling Reveals the Diversity of Epigenetic Regulation in the Rat Nervous System, ” Scientific Data 12, no. 1 (2025): 140. 39856121 10.1038/s41597-025-04432-y PMC11761061

[84] M. Stoeckius , C. Hafemeister , W. Stephenson , et al., “Simultaneous Epitope and Transcriptome Measurement in Single Cells, ” Nature Methods 14, no. 9 (2017): 865‐868. 28759029 10.1038/nmeth.4380 PMC5669064

[85] V. M. Peterson , K. X. Zhang , N. Kumar , et al., “Multiplexed Quantification of Proteins and Transcripts in Single Cells, ” Nature Biotechnology 35, no. 10 (2017): 936‐939. 10.1038/nbt.3973 28854175

[86] M. Angelo , S. C. Bendall , R. Finck , et al., “Multiplexed Ion Beam Imaging of human Breast Tumors, ” Nature Medicine 20, no. 4 (2014): 436‐442. 10.1038/nm.3488 PMC4110905 24584119

[87] Y. Goltsev , N. Samusik , J. Kennedy‐Darling , et al., “Deep Profiling of Mouse Splenic Architecture With CODEX Multiplexed Imaging, ” Cell 174, no. 4 (2018): 968‐981. e15. 30078711 10.1016/j.cell.2018.07.010 PMC6086938

[88] H. S. Bhatia , A. D. Brunner , F. Öztürk , et al., “Spatial Proteomics in Three‐dimensional Intact Specimens, ” Cell 185, no. 26 (2022): 5040‐5058. e19. 36563667 10.1016/j.cell.2022.11.021

[89] E. M. Unterauer , S. Shetab Boushehri , K. Jevdokimenko , et al., “Spatial Proteomics in Neurons at Single‐protein Resolution, ” Cell 187, no. 7 (2024): 1785‐1800. e16. 38552614 10.1016/j.cell.2024.02.045

[90] R. Sankowski , P. Süß , A. Benkendorff , et al., “Multiomic Spatial Landscape of Innate Immune Cells at human central Nervous System Borders, ” Nature Medicine 30, no. 1 (2024): 186‐198. 10.1038/s41591-023-02673-1 PMC10803260 38123840

[91] R. L. Hansen , Y. J. Lee , “High‐Spatial Resolution Mass Spectrometry Imaging: Toward Single Cell Metabolomics in Plant Tissues, ” Chemical Record 18, no. 1 (2018): 65‐77. 28685965 10.1002/tcr.201700027

[92] P. J. Ahl , R. A. Hopkins , W. W. Xiang , et al., “Met‐Flow, a Strategy for Single‐cell Metabolic Analysis Highlights Dynamic Changes in Immune Subpopulations, ” Communications Biology 3, no. 1 (2020): 305. 32533056 10.1038/s42003-020-1027-9 PMC7292829

[93] Z. Yuan , Q. Zhou , L. Cai , et al., “SEAM Is a Spatial Single Nuclear Metabolomics Method for Dissecting Tissue Microenvironment, ” Nature Methods 18, no. 10 (2021): 1223‐1232. 34608315 10.1038/s41592-021-01276-3

[94] Y. Zhu , Q. Zang , Z. Luo , J. He , R. Zhang , Z. Abliz , “An Organ‐Specific Metabolite Annotation Approach for Ambient Mass Spectrometry Imaging Reveals Spatial Metabolic Alterations of a Whole Mouse Body, ” Analytical Chemistry 94, no. 20 (2022): 7286‐7294. 35548855 10.1021/acs.analchem.2c00557

[95] R. de Ceglia , A. Ledonne , D. G. Litvin , et al., “Specialized Astrocytes Mediate Glutamatergic Gliotransmission in the CNS, ” Nature 622, no. 7981 (2023): 120‐129. 37674083 10.1038/s41586-023-06502-w PMC10550825

[96] S. Song , H. Oft , S. Metwally , et al., “Deletion of Slc9a1 in Cx3cr1+ Cells Stimulated Microglial Subcluster CREB1 Signaling and Microglia‑Oligodendrocyte Crosstalk, ” J Neuroinflammation 21, no. 1 (2024): 69. Published online 2024. 38509618 10.1186/s12974-024-03065-z PMC10953158

[97] E. Candelario‐Jalil , R. M. Dijkhuizen , T. Magnus , “Neuroinflammation, Stroke, Blood‐Brain Barrier Dysfunction, and Imaging Modalities, ” Stroke; A Journal of Cerebral Circulation 53, no. 5 (2022): 1473‐1486. 10.1161/STROKEAHA.122.036946 PMC9038693 35387495

[98] Y. Zhang , J. Li , Y. Zhao , et al., “Arresting the Bad Seed: HDAC3 Regulates Proliferation of Different Microglia After Ischemic Stroke, ” Science Advances 10, no. 10 (2024): eade6900. 38446877 10.1126/sciadv.ade6900 PMC10917353

[99] H. Li , P. Liu , B. Zhang , et al., “Acute Ischemia Induces Spatially and Transcriptionally Distinct Microglial Subclusters, ” Genome Med 15 (2023): 109. 38082331 10.1186/s13073-023-01257-5 PMC10712107

[100] X. Li , J. Lyu , R. Li , et al., “Single‐cell Transcriptomic Analysis of the Immune Cell Landscape in the Aged Mouse Brain After Ischemic Stroke, ” J Neuroinflammation 19 (2022): 83. 35392936 10.1186/s12974-022-02447-5 PMC8988369

[101] G. S. Kim , E. Harmon , M. C. Gutierrez , et al., “Single‐cell Analysis Identifies Ifi27l2a as a Gene Regulator of Microglial Inflammation in the Context of Aging and Stroke in Mice, ” Nature Communications 16, no. 1 (2025): 1639. 10.1038/s41467-025-56847-1 PMC11828888 39953063

[102] P. Zong , J. Feng , C. X. Li , et al., “Activation of Endothelial TRPM2 Exacerbates Blood–brain Barrier Degradation in Ischemic Stroke, ” Cardiovascular Research 120, no. 2 (2023): 188‐202. 10.1093/cvr/cvad126 PMC10936752 37595268

[103] X. Jiang , A. V. Andjelkovic , L. Zhu , et al., “Blood‐brain Barrier Dysfunction and Recovery After Ischemic Stroke, ” Progress in Neurobiology 163‐164 (2018): 144‐171. 10.1016/j.pneurobio.2017.10.001 PMC5886838 28987927

[104] Y. Li , B. Liu , T. Zhao , et al., “Comparative Study of Extracellular Vesicles Derived From Mesenchymal Stem Cells and Brain Endothelial Cells Attenuating Blood‐brain Barrier Permeability via Regulating Caveolin‐1‐dependent ZO‐1 and Claudin‐5 Endocytosis in Acute Ischemic Stroke, ” J Nanobiotechnology 21, no. 1 (2023): 70. 36855156 10.1186/s12951-023-01828-z PMC9976550

[105] Y. Zou , Y. Xu , X. Chen , Y. Wu , L. Fu , Y. Lv , “Research Progress on Leucine‐Rich Alpha‐2 Glycoprotein 1: A Review, ” Frontiers in Pharmacology 12 (2022): 809225. 35095520 10.3389/fphar.2021.809225 PMC8797156

[106] Z. Ruan , G. Cao , Y. Qian , et al., “Single‐cell RNA Sequencing Unveils Lrg1's Role in Cerebral Ischemia‒Reperfusion Injury by Modulating Various Cells, ” J Neuroinflammation 20, no. 1 (2023): 285. 38037097 10.1186/s12974-023-02941-4 PMC10687904

[107] S. Muhammad , W. Barakat , S. Stoyanov , et al., “The HMGB1 Receptor RAGE Mediates Ischemic Brain Damage, ” Journal of Neuroscience 28, no. 46 (2008): 12023‐12031. 19005067 10.1523/JNEUROSCI.2435-08.2008 PMC4597312

[108] Y. Xh , W. Y , G. Pp , “Lipoxin A4 Analogue Protects Brain and Reduces Inflammation in a Rat Model of Focal Cerebral Ischemia Reperfusion, ” Brain Research 1323 (2010): 174‐83. 20138164 10.1016/j.brainres.2010.01.079

[109] Y. Liu , X. Li , C. Cao , et al., “Critical Role of Slc22a8 in Maintaining Blood‐brain Barrier Integrity After Experimental Cerebral Ischemia‐reperfusion, ” Journal of Cerebral Blood Flow and Metabolism 45, no. 1 (2025): 85‐101. Published online July 28, 2024:271678×241264401. 39068534 10.1177/0271678X241264401 PMC11572098

[110] A. Patir , J. Barrington , S. Szymkowiak , et al., “Phenotypic and Spatial Heterogeneity of Brain Myeloid Cells After Stroke Is Associated With Cell Ontogeny, Tissue Damage, and Brain Connectivity, ” Cell Reports 43, no. 5 (2024): 114250. 38762882 10.1016/j.celrep.2024.114250

[111] S. Bardehle , M. Krüger , F. Buggenthin , et al., “Live Imaging of Astrocyte Responses to Acute Injury Reveals Selective Juxtavascular Proliferation, ” Nature Neuroscience 16, no. 5 (2013): 580‐586. 23542688 10.1038/nn.3371

[112] I. B. Wanner , M. A. Anderson , B. Song , et al., “Glial Scar Borders Are Formed by Newly Proliferated, Elongated Astrocytes That Interact to Corral Inflammatory and Fibrotic Cells via STAT3‐Dependent Mechanisms After Spinal Cord Injury, ” Journal of Neuroscience 33, no. 31 (2013): 12870‐12886. 23904622 10.1523/JNEUROSCI.2121-13.2013 PMC3728693

[113] R. D. Kim , A. E. Marchildon , P. W. Frazel , P. Hasel , A. X. Guo , S. A. Liddelow , “Temporal and Spatial Analysis of Astrocytes Following Stroke Identifies Novel Drivers of Reactivity, ” BioRxiv (2023). Published online November 20, 2023:2023.11.12.566710.

[114] E. Y. Scott , N. Safarian , D. L. Casasbuenas , et al., “Integrating Single‐cell and Spatially Resolved Transcriptomic Strategies to Survey the Astrocyte Response to Stroke in Male Mice, ” Nature Communications 15, no. 1 (2024): 1584. 10.1038/s41467-024-45821-y PMC10882052 38383565

[115] D. Bormann , M. Knoflach , E. Poreba , et al., “Single‐nucleus RNA Sequencing Reveals Glial Cell Type‐specific Responses to Ischemic Stroke in Male Rodents, ” Nature Communications 15, no. 1 (2024): 6232. 10.1038/s41467-024-50465-z PMC11266704 39043661

[116] Y. Jin , S. E. Dougherty , K. Wood , et al., “Regrowth of Serotonin Axons in the Adult Mouse Brain Following Injury, ” Neuron 91, no. 4 (2016): 748‐762. 27499084 10.1016/j.neuron.2016.07.024 PMC4990493

[117] P. Langhorne , J. Bernhardt , G. Kwakkel , “Stroke Rehabilitation, ” Lancet 377, no. 9778 (2011): 1693‐1702. 21571152 10.1016/S0140-6736(11)60325-5

[118] H. Abe , S. Jitsuki , W. Nakajima , et al., “CRMP2‐binding Compound, Edonerpic Maleate, Accelerates Motor Function Recovery From Brain Damage, ” Science 360, no. 6384 (2018): 50‐57. 29622647 10.1126/science.aao2300

[119] M. T. Joy , E. Ben Assayag , D. Shabashov‐Stone , et al., “CCR5 Is a Therapeutic Target for Recovery After Stroke and Traumatic Brain Injury, ” Cell 176, no. 5 (2019): 1143‐1157. e13. 30794775 10.1016/j.cell.2019.01.044 PMC7259116

[120] J. Mt , C. St , “Encouraging an Excitable Brain state: Mechanisms of Brain Repair in Stroke, ” Nature Reviews Neuroscience 22, no. 1 (2021): 38‐53. 33184469 10.1038/s41583-020-00396-7 PMC10625167

[121] G. H. D. Poplawski , R. Kawaguchi , E. Van Niekerk , et al., “Injured Adult Neurons Regress to an Embryonic Transcriptional Growth state, ” Nature 581, no. 7806 (2020): 77‐82. 32376949 10.1038/s41586-020-2200-5

[122] B. Han , S. Zhou , Y. Zhang , et al., “Integrating Spatial and Single‐cell Transcriptomics to Characterize the Molecular and Cellular Architecture of the Ischemic Mouse Brain, ” Science Translational Medicine 16, no. 733 (2024): eadg1323. 38324639 10.1126/scitranslmed.adg1323

[123] S. Song , L. Yu , M. N. Hasan , et al., “Elevated Microglial Oxidative Phosphorylation and Phagocytosis Stimulate Post‐stroke Brain Remodeling and Cognitive Function Recovery in Mice, ” Communications Biology 5, no. 1 (2022): 35. 35017668 10.1038/s42003-021-02984-4 PMC8752825

[124] S. Jin , C. F. Guerrero‐Juarez , L. Zhang , et al., “Inference and Analysis of Cell‐cell Communication Using CellChat, ” Nature Communications 12, no. 1 (2021): 1088. 10.1038/s41467-021-21246-9 PMC7889871 33597522

[125] C. Jin , Y. Shi , L. Shi , et al., “Leveraging Single‐cell RNA Sequencing to Unravel the Impact of Aging on Stroke Recovery Mechanisms in Mice, ” PNAS 120, no. 25 (2023): e2300012120. 37307473 10.1073/pnas.2300012120 PMC10288588

[126] J. Magid‐Bernstein , R. Girard , S. Polster , et al., “Cerebral Hemorrhage: Pathophysiology, Treatment, and Future Directions, ” Circulation Research 130, no. 8 (2022): 1204‐1229. 35420918 10.1161/CIRCRESAHA.121.319949 PMC10032582

[127] L. Gu , H. Chen , M. Sun , et al., “Unraveling Dynamic Immunological Landscapes in Intracerebral Hemorrhage: Insights From Single‐cell and Spatial Transcriptomic Profiling, ” MedComm 5, no. 7 (2024): e635. 38988493 10.1002/mco2.635 PMC11233862

[128] L. Ye , X. Tang , J. Zhong , et al., “Unraveling the Complex Pathophysiology of White Matter Hemorrhage in Intracerebral Stroke: A Single‐cell RNA Sequencing Approach, ” CNS neuroscience & therapeutics 30, no. 3 (2024): e14652. 38433011 10.1111/cns.14652 PMC10909628

[129] P. Zhang , C. Gao , Q. Guo , et al., “Single‐cell RNA Sequencing Reveals the Evolution of the Immune Landscape During Perihematomal Edema Progression After Intracerebral Hemorrhage, ” J Neuroinflammation 21, no. 1 (2024): 140. 38807233 10.1186/s12974-024-03113-8 PMC11131315

[130] Y. Huang , R. W. Mahley , “Apolipoprotein E: Structure and Function in Lipid Metabolism, Neurobiology, and Alzheimer's Diseases, ” Neurobiology of Disease 72, no. Pt A (2014): 3‐12. 25173806 10.1016/j.nbd.2014.08.025 PMC4253862

[131] Y. Huang , L. Mucke , “Alzheimer Mechanisms and Therapeutic Strategies, ” Cell 148, no. 6 (2012): 1204‐1222. 22424230 10.1016/j.cell.2012.02.040 PMC3319071

[132] N. Koutsodendris , M. R. Nelson , A. Rao , Y. Huang , “Apolipoprotein E and Alzheimer's Disease: Findings, Hypotheses, and Potential Mechanisms, ” Annu Rev Pathol 17 (2022): 73‐99. 34460318 10.1146/annurev-pathmechdis-030421-112756

[133] Y. Shi , M. Manis , J. Long , et al., “Microglia Drive APOE‐dependent Neurodegeneration in a Tauopathy Mouse Model, ” Journal of Experimental Medicine 216, no. 11 (2019): 2546‐2561. 31601677 10.1084/jem.20190980 PMC6829593

[134] J. Therriault , A. L. Benedet , T. A. Pascoal , et al., “APOEε4 potentiates the Relationship Between Amyloid‐β and Tau Pathologies, ” Molecular Psychiatry 26, no. 10 (2021): 5977‐5988. 32161362 10.1038/s41380-020-0688-6 PMC8758492

[135] C. Wang , R. Najm , Q. Xu , et al., “Gain of Toxic Apolipoprotein E4 Effects in human iPSC‐derived Neurons Is Ameliorated by a Small‐molecule Structure Corrector, ” Nature Medicine 24, no. 5 (2018): 647‐657. 10.1038/s41591-018-0004-z PMC5948154 29632371

[136] J. Zhao , Y. Fu , Y. Yamazaki , et al., “APOE4 exacerbates Synapse Loss and Neurodegeneration in Alzheimer's disease Patient iPSC‐derived Cerebral Organoids, ” Nature Communications 11, no. 1 (2020): 5540. 10.1038/s41467-020-19264-0 PMC7608683 33139712

[137] J. W. Blanchard , L. A. Akay , J. Davila‐Velderrain , et al., “APOE4 impairs Myelination via Cholesterol Dysregulation in Oligodendrocytes, ” Nature 611, no. 7937 (2022): 769‐779. 36385529 10.1038/s41586-022-05439-w PMC9870060

[138] G. Barisano , K. Kisler , B. Wilkinson , et al., “A “Multi‐omics” Analysis of Blood–brain Barrier and Synaptic Dysfunction in *APOE4* Mice, ” Journal of Experimental Medicine 219, no. 11 (2022): e20221137. 36040482 10.1084/jem.20221137 PMC9435921

[139] L. Brase , S. F. You , R. D'Oliveira Albanus , et al., “Single‐nucleus RNA‐sequencing of Autosomal Dominant Alzheimer Disease and Risk Variant Carriers, ” Nature Communications 14, no. 1 (2023): 2314. 10.1038/s41467-023-37437-5 PMC10121712 37085492

[140] M. S. Haney , R. Pálovics , C. N. Munson , et al., “APOE4/4 is Linked to Damaging Lipid Droplets in Alzheimer's Disease Microglia, ” Nature 628, no. 8006 (2024): 154‐161. 38480892 10.1038/s41586-024-07185-7 PMC10990924

[141] Z. S. Ji , R. E. Pitas , R. W. Mahley , “Differential Cellular Accumulation/Retention of Apolipoprotein E Mediated by Cell Surface Heparan Sulfate Proteoglycans. Apolipoproteins E3 and E2 Greater Than e4, ” Journal of Biological Chemistry 273, no. 22 (1998): 13452‐13460. 9593678 10.1074/jbc.273.22.13452

[142] Y. Yamauchi , N. Deguchi , C. Takagi , et al., “Role of the N‐ and C‐terminal Domains in Binding of Apolipoprotein E Isoforms to Heparan Sulfate and Dermatan Sulfate: A Surface Plasmon Resonance Study, ” Biochemistry 47, no. 25 (2008): 6702‐6710. 18507396 10.1021/bi8003999 PMC2844924

[143] J. F. Arboleda‐Velasquez , F. Lopera , M. O'Hare , “Resistance to Autosomal Dominant Alzheimer's Disease in an APOE3 Christchurch Homozygote: A Case Report, ” Nature Medicine 25, no. 11 (2019): 1680‐1683. 10.1038/s41591-019-0611-3 PMC6898984 31686034

[144] C. C. Liu , M. E. Murray , X. Li , et al., “APOE3‐Jacksonville (V236E) Variant Reduces Self‐aggregation and Risk of Dementia, ” Science Translational Medicine 13, no. 613 (2021): eabc9375. 34586832 10.1126/scitranslmed.abc9375 PMC8824726

[145] M. C. Almeida , S. J. Eger , C. He , et al., “Single‐nucleus RNA Sequencing Demonstrates an Autosomal Dominant Alzheimer's Disease Profile and Possible Mechanisms of Disease Protection, ” Neuron 112, no. 11 (2024): 1778‐1794. e7. 38417436 10.1016/j.neuron.2024.02.009 PMC11156559

[146] B. Chambraud , E. Sardin , J. Giustiniani , et al., “A Role for FKBP52 in Tau Protein Function, ” PNAS 107, no. 6 (2010): 2658‐2663. 20133804 10.1073/pnas.0914957107 PMC2823896

[147] J. N. Rauch , G. Luna , E. Guzman , et al., “LRP1 is a Master Regulator of Tau Uptake and Spread, ” Nature 580, no. 7803 (2020): 381‐385. 32296178 10.1038/s41586-020-2156-5 PMC7687380

[148] M. R. Nelson , “The APOE‐R136S Mutation Protects Against APOE4‐driven Tau Pathology, Neurodegeneration and Neuroinflammation, ” Nature Neuroscience 26 (2023): 2104‐2121. 37957317 10.1038/s41593-023-01480-8 PMC10689245

[149] S. Alford , D. Patel , N. Perakakis , C. S. Mantzoros , “Obesity as a Risk Factor for Alzheimer's Disease: Weighing the Evidence, ” Obesity Reviews 19, no. 2 (2018): 269‐280. 29024348 10.1111/obr.12629

[150] A. Singh‐Manoux , A. Dugravot , M. Shipley , et al., “Obesity Trajectories and Risk of Dementia: 28 Years of Follow‐up in the Whitehall II Study, ” Alzheimers Dement 14, no. 2 (2018): 178‐186. 28943197 10.1016/j.jalz.2017.06.2637 PMC5805839

[151] S. Suzzi , T. Croese , A. Ravid , et al., “N‐acetylneuraminic Acid Links Immune Exhaustion and Accelerated Memory Deficit in Diet‐induced Obese Alzheimer's disease Mouse Model, ” Nature Communications 14, no. 1 (2023): 1293. 10.1038/s41467-023-36759-8 PMC9998639 36894557

[152] B. M. Bettcher , M. G. Tansey , G. Dorothée , M. T. Heneka , “Peripheral and central Immune System Crosstalk in Alzheimer Disease — a Research Prospectus, ” Nature reviews Neurology 17, no. 11 (2021): 689‐701. 34522039 10.1038/s41582-021-00549-x PMC8439173

[153] Y. Lu , C. Saibro‐Girardi , N. F. Fitz , et al., “Multi‐transcriptomics Reveals Brain Cellular Responses to Peripheral Infection in Alzheimer's disease Model Mice, ” Cell reports 42, no. 7 (2023): 112785. 37436901 10.1016/j.celrep.2023.112785 PMC10530196

[154] Y. Zhang , H. Chen , R. Li , K. Sterling , W. Song , “Amyloid β‐based Therapy for Alzheimer's Disease: Challenges, Successes and Future, ” Signal Transduct Target Ther 8, no. 1 (2023): 248. 37386015 10.1038/s41392-023-01484-7 PMC10310781

[155] V. Gazestani , T. Kamath , N. M. Nadaf , et al., “Early Alzheimer's Disease Pathology in human Cortex Involves Transient Cell States, ” Cell 186, no. 20 (2023): 4438‐4453. e23. 37774681 10.1016/j.cell.2023.08.005 PMC11107481

[156] A. Ishii , J. A. Pathoulas , O. MoustafaFathy Omar , et al., “Contribution of Amyloid Deposition From Oligodendrocytes in a Mouse Model of Alzheimer's Disease, ” Mol Neurodegeneration 19, no. 1 (2024): 83. 10.1186/s13024-024-00759-z PMC11568619 39548583

[157] N. Habib , C. McCabe , S. Medina , et al., “Disease‐associated Astrocytes in Alzheimer's Disease and Aging, ” Nature Neuroscience 23, no. 6 (2020): 701‐706. 32341542 10.1038/s41593-020-0624-8 PMC9262034

[158] J. Xie , Y. Lan , C. Zou , et al., “Single‐nucleus Analysis Reveals Microenvironment‐specific Neuron and Glial Cell Enrichment in Alzheimer's Disease, ” BMC Genomics [Electronic Resource] 25, no. 1 (2024): 526. 38807051 10.1186/s12864-024-10447-3 PMC11134750

[159] E. Giraldo , A. Lloret , T. Fuchsberger , J. Viña , “Aβ and Tau Toxicities in Alzheimer's Are Linked via Oxidative Stress‐induced p38 Activation: Protective Role of Vitamin E, ” Redox Biology 2 (2014): 873‐877. 25061569 10.1016/j.redox.2014.03.002 PMC4099506

[160] S. Tsartsalis , H. Sleven , N. Fancy , et al., “A Single Nuclear Transcriptomic Characterisation of Mechanisms Responsible for Impaired Angiogenesis and Blood‐brain Barrier Function in Alzheimer's Disease, ” Nature Communications 15, no. 1 (2024): 2243. 10.1038/s41467-024-46630-z PMC10933340 38472200

[161] N. N. Fancy , A. M. Smith , A. Caramello , et al., “Characterisation of Premature Cell Senescence in Alzheimer's Disease Using Single Nuclear Transcriptomics, ” Acta Neuropathologica 147, no. 1 (2024): 78. 38695952 10.1007/s00401-024-02727-9 PMC11065703

[162] Y. Chen , Y. Yu , “Tau and Neuroinflammation in Alzheimer's Disease: Interplay Mechanisms and Clinical Translation, ” J Neuroinflammation 20, no. 1 (2023): 165. 37452321 10.1186/s12974-023-02853-3 PMC10349496

[163] A. Wachter , M. E. Woodbury , S. Lombardo , et al., “Landscape of Brain Myeloid Cell Transcriptome Along the Spatiotemporal Progression of Alzheimer's Disease Reveals Distinct Sequential Responses to Aβ and Tau, ” Acta Neuropathologica 147, no. 1 (2024): 65. 38557897 10.1007/s00401-024-02704-2 PMC10984903

[164] R. Duan , A. Liu , Y. Sun , et al., “Loss of Smek1 Induces Tauopathy and Triggers Neurodegeneration by Regulating Microtubule Stability, ” Advanced Science 11, no. 40 (2024): 2400584. 39206808 10.1002/advs.202400584 PMC11516166

[165] H. Fu , J. Hardy , K. E. Duff , “Selective Vulnerability in Neurodegenerative Diseases, ” Nature Neuroscience 21, no. 10 (2018): 1350‐1358. 30250262 10.1038/s41593-018-0221-2 PMC6360529

[166] R. Praschberger , S. Kuenen , N. Schoovaerts , et al., “Neuronal Identity Defines α‐synuclein and Tau Toxicity, ” Neuron 111, no. 10 (2023): 1577‐1590. e11. 36948206 10.1016/j.neuron.2023.02.033 PMC10191620

[167] J. Aguila , S. Cheng , N. Kee , et al., “Spatial RNA Sequencing Identifies Robust Markers of Vulnerable and Resistant Human Midbrain Dopamine Neurons and Their Expression in Parkinson's Disease, ” Front Mol Neurosci 14 (2021): 699562. 34305528 10.3389/fnmol.2021.699562 PMC8297217

[168] L. Lin , O. Isacson , “Axonal Growth Regulation of Fetal and Embryonic Stem Cell‐derived Dopaminergic Neurons by Netrin‐1 and Slits, ” Stem Cells 24, no. 11 (2006): 2504‐2513. 16840550 10.1634/stemcells.2006-0119 PMC2613222

[169] I. Bezprozvanny , “Calcium Signaling and Neurodegenerative Diseases, ” Trends in Molecular Medicine 15, no. 3 (2009): 89‐100. 19230774 10.1016/j.molmed.2009.01.001 PMC3226745

[170] M. Maor‐Nof , Z. Shipony , R. Lopez‐Gonzalez , et al., “53 is a central Regulator Driving Neurodegeneration Caused by C9orf72 Poly(PR), ” Cell 184, no. 3 (2021): 689. 33482083 10.1016/j.cell.2020.12.025 PMC7886018

[171] C. Y. Kao , M. Xu , L. Wang , et al., “Elevated COUP‐TFII Expression in Dopaminergic Neurons Accelerates the Progression of Parkinson's Disease Through Mitochondrial Dysfunction, ” PLos Genet 16, no. 6 (2020): e1008868. 32579581 10.1371/journal.pgen.1008868 PMC7340320

[172] S. Smajić , C. A. Prada‐Medina , Z. Landoulsi , et al., “Single‐cell Sequencing of human Midbrain Reveals Glial Activation and a Parkinson‐specific Neuronal state, ” Brain 145, no. 3 (2022): 964‐978. 34919646 10.1093/brain/awab446 PMC9050543

[173] I. Brunk , C. Blex , D. Speidel , N. Brose , G. Ahnert‐Hilger , “Ca2+‐dependent Activator Proteins of Secretion Promote Vesicular Monoamine Uptake, ” Journal of Biological Chemistry 284, no. 2 (2009): 1050‐1056. 19008227 10.1074/jbc.M805328200

[174] P. Reinhardt , B. Schmid , L. F. Burbulla , et al., “Genetic Correction of a LRRK2 Mutation in human iPSCs Links Parkinsonian Neurodegeneration to ERK‐dependent Changes in Gene Expression, ” Cell Stem Cell 12, no. 3 (2013): 354‐367. 23472874 10.1016/j.stem.2013.01.008

[175] K. Tiklová , Å. K. Björklund , L. Lahti , et al., “Single‐cell RNA Sequencing Reveals Midbrain Dopamine Neuron Diversity Emerging During Mouse Brain Development, ” Nature Communications 10, no. 1 (2019): 581. 10.1038/s41467-019-08453-1 PMC6362095 30718509

[176] H. Braak , U. Rüb , K. Del Tredici , “Cognitive Decline Correlates With Neuropathological Stage in Parkinson's Disease, ” Journal of the Neurological Sciences 248, no. 1 (2006): 255‐258. 16814807 10.1016/j.jns.2006.05.011

[177] D. J. Irwin , M. Grossman , D. Weintraub , et al., “Neuropathological and Genetic Correlates of Survival and Dementia Onset in Synucleinopathies: A Retrospective Analysis, ” Lancet Neurology 16, no. 1 (2017): 55‐65. 27979356 10.1016/S1474-4422(16)30291-5 PMC5181646

[178] C. Smith , N. Malek , K. Grosset , B. Cullen , S. Gentleman , D. G. Grosset , “Neuropathology of Dementia in Patients With Parkinson's Disease: A Systematic Review of Autopsy Studies, ” Journal of Neurology, Neurosurgery, and Psychiatry 90, no. 11 (2019): 1234‐1243. 31444276 10.1136/jnnp-2019-321111

[179] L. Yu , T. Wang , R. S. Wilson , et al., “Common Age‐related Neuropathologies and Yearly Variability in Cognition, ” Ann Clin Transl Neurol 6, no. 11 (2019): 2140‐2149. 31568713 10.1002/acn3.50857 PMC6856601

[180] T. M. Goralski , L. Meyerdirk , L. Breton , et al., “Spatial Transcriptomics Reveals Molecular Dysfunction Associated With Cortical Lewy Pathology, ” Nature Communications 15 (2024): 2642. 10.1038/s41467-024-47027-8 PMC10966039 38531900

[181] L. Horan‐Portelance , M. Iba , D. J. Acri , J. R. Gibbs , M. R. Cookson , “Imaging Spatial Transcriptomics Reveals Molecular Patterns of Vulnerability to Pathology in a Transgenic α‐synucleinopathy Model, ” BioRxiv (2024). Published online December 14, 2024.

[182] L. Rojanathammanee , E. J. Murphy , C. K. Combs , “Expression of Mutant Alpha‐synuclein Modulates Microglial Phenotype in Vitro, ” J Neuroinflammation 8 (2011): 44. 21554732 10.1186/1742-2094-8-44 PMC3104357

[183] A. S. Harms , C. J. Barnum , K. A. Ruhn , et al., “Delayed Dominant‐Negative TNF Gene Therapy Halts Progressive Loss of Nigral Dopaminergic Neurons in a Rat Model of Parkinson's Disease, ” Molecular Therapy 19, no. 1 (2011): 46‐52. 20959812 10.1038/mt.2010.217 PMC3017447

[184] Q. Liu , Z. Liu , W. Xie , et al., “Single‐cell Sequencing of the substantia nigra Reveals Microglial Activation in a Model of MPTP, ” Frontiers in aging neuroscience 16 (2024): 1390310. 38952478 10.3389/fnagi.2024.1390310 PMC11215054

[185] H. L. Smith , O. J. Freeman , A. J. Butcher , et al., “Astrocyte Unfolded Protein Response Induces a Specific Reactivity State That Causes Non‐Cell‐Autonomous Neuronal Degeneration, ” Neuron 105, no. 5 (2020): 855. 31924446 10.1016/j.neuron.2019.12.014 PMC7054837

[186] J. G. Sheng , K. Ito , R. D. Skinner , “In Vivo and In Vitro Evidence Supporting a Role for the Inflammatory Cytokine Interleukin‐1 as a Driving Force in Alzheimer Pathogenesis, ” Neurobiology of Aging 17, no. 5 (1996): 761‐766, Accessed January 25, 2024 https://www.ncbi.nlm.nih.gov/pmc/articles/PMC3886636/. 8892349 10.1016/0197-4580(96)00104-2 PMC3886636

[187] K. Sathe , W. Maetzler , J. D. Lang , et al., “S100B is Increased in Parkinson's Disease and Ablation Protects Against MPTP‐induced Toxicity Through the RAGE and TNF‐α Pathway, ” Brain 135, no. Pt 11 (2012): 3336‐3347. 23169921 10.1093/brain/aws250 PMC3501971

[188] D. Agarwal , C. Sandor , V. Volpato , et al., “A Single‐cell Atlas of the human Substantia nigra Reveals Cell‐specific Pathways Associated With Neurological Disorders, ” Nature Communications 11 (2020): 4183. 10.1038/s41467-020-17876-0 PMC7442652 32826893

[189] P. Wang , L. Yao , M. Luo , et al., “Single‐cell Transcriptome and TCR Profiling Reveal Activated and Expanded T Cell Populations in Parkinson's Disease, ” Cell Discovery 7, no. 1 (2021): 52. 34282123 10.1038/s41421-021-00280-3 PMC8289849

[190] P. Wang , M. Luo , W. Zhou , et al., “Global Characterization of Peripheral B Cells in Parkinson's Disease by Single‐Cell RNA and BCR Sequencing, ” Frontiers in Immunology 13 (2022), Accessed January 30, 2024 https://www.frontiersin.org/articles/10.3389/fimmu.2022.814239. 10.3389/fimmu.2022.814239 PMC8888848 35250991

[191] A. M. Schonhoff , D. A. Figge , G. P. Williams , et al., “Border‐associated Macrophages Mediate the Neuroinflammatory Response in an Alpha‐synuclein Model of Parkinson disease, ” Nature Communications 14, no. 1 (2023): 3754. 10.1038/s41467-023-39060-w PMC10293214 37365181

[192] G. P. Williams , A. M. Schonhoff , A. Jurkuvenaite , N. J. Gallups , D. G. Standaert , A. S. Harms , “CD4 T Cells Mediate Brain Inflammation and Neurodegeneration in a Mouse Model of Parkinson's Disease, ” Brain 144, no. 7 (2021): 2047‐2059. 33704423 10.1093/brain/awab103 PMC8370411

[193] R. Stupp , W. P. Mason , M. J. van den Bent , et al., “Radiotherapy plus Concomitant and Adjuvant Temozolomide for Glioblastoma, ” New England Journal of Medicine 352, no. 10 (2005): 987‐996. 15758009 10.1056/NEJMoa043330

[194] C. Neftel , J. Laffy , M. G. Filbin , et al., “An Integrative Model of Cellular States, Plasticity, and Genetics for Glioblastoma, ” Cell 178, no. 4 (2019): 835‐849. e21. 31327527 10.1016/j.cell.2019.06.024 PMC6703186

[195] Y. Ren , Z. Huang , L. Zhou , et al., “Spatial Transcriptomics Reveals Niche‐specific Enrichment and Vulnerabilities of Radial Glial Stem‐Like Cells in Malignant Gliomas, ” Nature Communications 14 (2023): 1028. 10.1038/s41467-023-36707-6 PMC9950149 36823172

[196] R. Lm , W. Okn , M. G , et al., “Gradient of Developmental and Injury Response Transcriptional States Defines Functional Vulnerabilities Underpinning Glioblastoma Heterogeneity, ” Nature Cancer 2, no. 2 (2021): 157‐173. 35122077 10.1038/s43018-020-00154-9

[197] M. L. Suvà , I. Tirosh , “The Glioma Stem Cell Model in the Era of Single‐Cell Genomics, ” Cancer Cell 37, no. 5 (2020): 630‐636. 32396858 10.1016/j.ccell.2020.04.001

[198] A. Bhaduri , E. Di Lullo , D. Jung , et al., “Outer Radial Glia‐Like Cancer Stem Cells Contribute to Heterogeneity of Glioblastoma, ” Cell Stem Cell 26, no. 1 (2020): 48‐63. e6. 31901251 10.1016/j.stem.2019.11.015 PMC7029801

[199] A. A. Pollen , T. J. Nowakowski , J. Chen , et al., “Molecular Identity of Human Outer Radial Glia during Cortical Development, ” Cell 163, no. 1 (2015): 55‐67. 26406371 10.1016/j.cell.2015.09.004 PMC4583716

[200] A. A. Hamed , D. J. Kunz , I. El‐Hamamy , et al., “A Brain Precursor Atlas Reveals the Acquisition of Developmental‐Like States in Adult Cerebral Tumours, ” Nature Communications 13 (2022): 4178. 10.1038/s41467-022-31408-y PMC9296666 35853870

[201] M. Castellan , A. Guarnieri , A. Fujimura , et al., “Single‐cell Analyses Reveal YAP/TAZ as Regulators of Stemness and Cell Plasticity in Glioblastoma, ” Nat Cancer 2, no. 2 (2021): 174‐188. 33644767 10.1038/s43018-020-00150-z PMC7116831

[202] C. Guetta‐Terrier , D. Karambizi , B. Akosman , et al., “Chi3l1 Is a Modulator of Glioma Stem Cell States and a Therapeutic Target in Glioblastoma, ” Cancer Research 83, no. 12 (2023): 1984‐1999. 37101376 10.1158/0008-5472.CAN-21-3629 PMC10267676

[203] L. Wang , J. Jung , H. Babikir , et al., “A Single‐cell Atlas of Glioblastoma Evolution Under Therapy Reveals Cell‐intrinsic and Cell‐extrinsic Therapeutic Targets, ” Nat Cancer 3, no. 12 (2022): 1534‐1552. 36539501 10.1038/s43018-022-00475-x PMC9767870

[204] D. R. Grimes , M. Jansen , R. J. Macauley , J. G. Scott , D. Basanta , “Evidence for Hypoxia Increasing the Tempo of Evolution in Glioblastoma, ” British Journal of Cancer 123, no. 10 (2020): 1562‐1569. 32848201 10.1038/s41416-020-1021-5 PMC7653934

[205] V. Bhandari , C. Hoey , L. Y. Liu , et al., “Molecular Landmarks of Tumor Hypoxia Across Cancer Types, ” Nature Genetics 51, no. 2 (2019): 308‐318. 30643250 10.1038/s41588-018-0318-2

[206] D. H. Heiland , A. Gaebelein , M. Börries , et al., “Microenvironment‐Derived Regulation of HIF Signaling Drives Transcriptional Heterogeneity in Glioblastoma Multiforme, ” Molecular Cancer Research 16, no. 4 (2018): 655‐668. 29330292 10.1158/1541-7786.MCR-17-0680

[207] K. R. Luoto , R. Kumareswaran , R. G. Bristow , “Tumor Hypoxia as a Driving Force in Genetic Instability, ” Genome Integrity 4, no. 1 (2013): 5. 24152759 10.1186/2041-9414-4-5 PMC4016142

[208] K. C. Johnson , K. J. Anderson , E. T. Courtois , et al., “Single‐cell Multimodal Glioma Analyses Identify Epigenetic Regulators of Cellular Plasticity and Environmental Stress Response, ” Nature Genetics 53, no. 10 (2021): 1456‐1468. 34594038 10.1038/s41588-021-00926-8 PMC8570135

[209] A. Kathagen , A. Schulte , G. Balcke , et al., “Hypoxia and Oxygenation Induce a Metabolic Switch Between Pentose Phosphate Pathway and Glycolysis in Glioma Stem‐Like Cells, ” Acta Neuropathologica 126, no. 5 (2013): 763‐780. 24005892 10.1007/s00401-013-1173-y

[210] V. M. Ravi , P. Will , J. Kueckelhaus , et al., “Spatially Resolved Multi‐omics Deciphers Bidirectional Tumor‐host Interdependence in Glioblastoma, ” Cancer Cell 40, no. 6 (2022): 639‐655. e13. 35700707 10.1016/j.ccell.2022.05.009

[211] E. F. Simonds , E. D. Lu , O. Badillo , et al., “Deep Immune Profiling Reveals Targetable Mechanisms of Immune Evasion in Immune Checkpoint Inhibitor‐refractory Glioblastoma, ” Journal for ImmunoTherapy of Cancer 9, no. 6 (2021): e002181. 34083417 10.1136/jitc-2020-002181 PMC8183210

[212] A. Xiong , J. Zhang , Y. Chen , Y. Zhang , F. Yang , “Integrated Single‐cell Transcriptomic Analyses Reveal That GPNMB‐high Macrophages Promote PN‐MES Transition and Impede T Cell Activation in GBM, ” EBioMedicine 83 (2022): 104239. 36054938 10.1016/j.ebiom.2022.104239 PMC9437813

[213] Q. W. Wang , L. H. Sun , Y. Zhang , et al., “MET Overexpression Contributes to STAT4‐PD‐L1 Signaling Activation Associated With Tumor‐associated, Macrophages‐mediated Immunosuppression in Primary Glioblastomas, ” Journal for ImmunoTherapy of Cancer 9, no. 10 (2021): e002451. 34667077 10.1136/jitc-2021-002451 PMC8527154

[214] A. H. Lee , L. Sun , A. Y. Mochizuki , et al., “Neoadjuvant PD‐1 Blockade Induces T Cell and cDC1 Activation but Fails to Overcome the Immunosuppressive Tumor Associated Macrophages in Recurrent Glioblastoma, ” Nature Communications 12 (2021): 6938. 10.1038/s41467-021-26940-2 PMC8626557 34836966

[215] X. Jin , L. J. Y. Kim , Q. Wu , et al., “Targeting Glioma Stem Cells Through Combined BMI1 and EZH2 Inhibition, ” Nature Medicine 23, no. 11 (2017): 1352‐1361. 10.1038/nm.4415 PMC5679732 29035367

[216] T. Hara , R. Chanoch‐Myers , N. D. Mathewson , et al., “Interactions Between Cancer Cells and Immune Cells Drive Transitions to Mesenchymal‐Like States in Glioblastoma, ” Cancer Cell 39, no. 6 (2021): 779‐792. e11. 34087162 10.1016/j.ccell.2021.05.002 PMC8366750

[217] C. E. Eyler , H. Matsunaga , V. Hovestadt , S. J. Vantine , P. van Galen , B. E. Bernstein , “Single‐cell Lineage Analysis Reveals Genetic and Epigenetic Interplay in Glioblastoma Drug Resistance, ” Genome biology 21 (2020): 174. 32669109 10.1186/s13059-020-02085-1 PMC7364565

[218] Z. Chen , N. Soni , G. Pinero , et al., “Monocyte Depletion Enhances Neutrophil Influx and Proneural to Mesenchymal Transition in Glioblastoma, ” Nature Communications 14, no. 1 (2023): 1839. 10.1038/s41467-023-37361-8 PMC10070461 37012245

[219] H. J. Kim , J. H. Park , H. C. Kim , C. W. Kim , I. Kang , H. K. Lee , “Blood Monocyte‐derived CD169+ Macrophages Contribute to Antitumor Immunity Against Glioblastoma, ” Nature Communications 13 (2022): 6211. 10.1038/s41467-022-34001-5 PMC9585054 36266311

[220] Z. Chen , C. J. Herting , J. L. Ross , et al., “Genetic Driver Mutations Introduced in Identical Cell‐of‐origin in Murine Glioblastoma Reveal Distinct Immune Landscapes but Similar Response to Checkpoint Blockade, ” Glia 68, no. 10 (2020): 2148. 32639068 10.1002/glia.23883 PMC7512141

[221] D. Douillet , C. C. Sze , C. Ryan , et al., “Uncoupling Histone H3K4 Trimethylation From Developmental Gene Expression via an Equilibrium of COMPASS, Polycomb and DNA Methylation, ” Nature Genetics 52, no. 6 (2020): 615‐625. 32393859 10.1038/s41588-020-0618-1 PMC7790509

[222] R. Chaligne , F. Gaiti , D. Silverbush , et al., “Epigenetic Encoding, Heritability and Plasticity of Glioma Transcriptional Cell States, ” Nature Genetics 53, no. 10 (2021): 1469‐1479. 34594037 10.1038/s41588-021-00927-7 PMC8675181

[223] C. M. Pretzsch , M. Arenella , J. P. Lerch , et al., “Patterns of Brain Maturation in Autism and Their Molecular Associations, ” JAMA Psychiatry 81, no. 12 (2024): 1253‐1264. 39412777 10.1001/jamapsychiatry.2024.3194 PMC11581727

[224] O. Peñagarikano , B. S. Abrahams , E. I. Herman , et al., “Absence of CNTNAP2 Leads to Epilepsy, Neuronal Migration Abnormalities, and Core Autism‐related Deficits, ” Cell 147, no. 1 (2011): 235‐246. 21962519 10.1016/j.cell.2011.08.040 PMC3390029

[225] M. T. Lazaro , J. Taxidis , T. Shuman , et al., “Reduced Prefrontal Synaptic Connectivity and Disturbed Oscillatory Population Dynamics in the CNTNAP2 Model of Autism, ” Cell reports 27, no. 9 (2019): 2567‐2578. e6. 31141683 10.1016/j.celrep.2019.05.006 PMC6553483

[226] W. E. Jang , J. H. Park , G. Park , et al., “Cntnap2‐dependent Molecular Networks in Autism Spectrum Disorder Revealed Through an Integrative Multi‐omics Analysis, ” Molecular Psychiatry 28, no. 2 (2023): 810‐821. 36253443 10.1038/s41380-022-01822-1 PMC9908544

[227] F. Şimşek , Ü. Işık , E. Aktepe , F. Kılıç , F. B. Şirin , M. Bozkurt , “Comparison of Serum VEGF, IGF‐1, and HIF‐1α Levels in Children With Autism Spectrum Disorder and Healthy Controls, ” Journal of Autism and Developmental Disorders 51, no. 10 (2021): 3564‐3574. 33389301 10.1007/s10803-020-04820-w

[228] J. Cui , H. Li , C. Hu , et al., “Unraveling Pathogenesis and Potential Biomarkers for Autism Spectrum Disorder Associated With HIF1A Pathway Based on Machine Learning and Experiment Validation, ” Neurobiology of Disease 204 (2025): 106763. 39657846 10.1016/j.nbd.2024.106763

[229] D. Majerczyk , E. G. Ayad , K. L. Brewton , P. Saing , P. C. Hart , “Systemic Maternal Inflammation Promotes ASD via IL‐6 and IFN‐γ, ” Bioscience Reports 42, no. 11 (2022): BSR20220713. 36300375 10.1042/BSR20220713 PMC9670245

[230] B. Wamsley , L. Bicks , Y. Cheng , et al., “Molecular Cascades and Cell Type–specific Signatures in ASD Revealed by Single‐cell Genomics, ” Science 384, no. 6698 (2024): eadh2602. 38781372 10.1126/science.adh2602

[231] T. T. Logan , S. Villapol , A. J. Symes , “TGF‐β Superfamily Gene Expression and Induction of the Runx1 Transcription Factor in Adult Neurogenic Regions After Brain Injury, ” PLoS ONE 8, no. 3 (2013): e59250. 23555640 10.1371/journal.pone.0059250 PMC3605457

[232] K. M. Dhandapani , M. Hadman , L. De Sevilla , M. F. Wade , V. B. Mahesh , D. W. Brann , “Astrocyte Protection of Neurons: Role of Transforming Growth Factor‐beta Signaling via a c‐Jun‐AP‐1 Protective Pathway, ” Journal of Biological Chemistry 278, no. 44 (2003): 43329‐43339. 12888549 10.1074/jbc.M305835200

[233] L. Wang , C. Wang , J. A. Moriano , et al., “Molecular and Cellular Dynamics of the Developing human Neocortex, ” Nature (2025). Published online January 8, 2025. 10.1038/s41586-024-08351-7 PMC12589127 39779846

[234] M. Neumann , D. M. Sampathu , L. K. Kwong , et al., “Ubiquitinated TDP‐43 in Frontotemporal Lobar Degeneration and Amyotrophic Lateral Sclerosis, ” Science 314, no. 5796 (2006): 130‐133. 17023659 10.1126/science.1134108

[235] F. Limone , D. A. Mordes , A. Couto , et al., “Single‐nucleus Sequencing Reveals Enriched Expression of Genetic Risk Factors in Extratelencephalic Neurons Sensitive to Degeneration in ALS, ” Nat Aging 4, no. 7 (2024): 984‐997. 38907103 10.1038/s43587-024-00640-0 PMC11257952

[236] S. S. Pineda , H. Lee , M. J. Ulloa‐Navas , et al., “Single‐cell Dissection of the human Motor and Prefrontal Cortices in ALS and FTLD, ” Cell 187, no. 8 (2024): 1971‐1989. e16. 38521060 10.1016/j.cell.2024.02.031 PMC11086986

[237] S. Coupel , A. Moreau , M. Hamidou , V. Horejsi , J. P. Soulillou , B. Charreau , “Expression and Release of Soluble HLA‐E Is an Immunoregulatory Feature of Endothelial Cell Activation, ” Blood 109, no. 7 (2007): 2806‐2814. 17179229 10.1182/blood-2006-06-030213

[238] Y. Shi , L. Huang , H. Dong , et al., “Decoding the Spatiotemporal Regulation of Transcription Factors During human Spinal Cord Development, ” Cell Research 34, no. 3 (2024): 193‐213. 38177242 10.1038/s41422-023-00897-x PMC10907391

[239] T. Itou , K. Fujita , Y. Okuzono , et al., “Th17 and Effector CD8 T Cells Relate to Disease Progression in Amyotrophic Lateral Sclerosis: A Case Control Study, ” J Neuroinflammation 21, no. 1 (2024): 331. 39731185 10.1186/s12974-024-03327-w PMC11674182

[240] E. Álvarez‐Sánchez , Á. Carbayo , N. Valle‐Tamayo , et al., “Single‐cell RNA Sequencing Highlights the Role of Distinct Natural Killer Subsets in Sporadic Amyotrophic Lateral sclerosis, ” J Neuroinflammation 22, no. 1 (2025): 15. 39849490 10.1186/s12974-025-03347-0 PMC11756089

[241] I. Blumcke , R. Spreafico , G. Haaker , et al., “Histopathological Findings in Brain Tissue Obtained During Epilepsy Surgery, ” New England Journal of Medicine 377, no. 17 (2017): 1648‐1656. 29069555 10.1056/NEJMoa1703784

[242] I. Blumcke , F. Cendes , H. Miyata , M. Thom , E. Aronica , I. Najm , “Toward a Refined Genotype‐phenotype Classification Scheme for the International Consensus Classification of Focal Cortical Dysplasia, ” Brain Pathology 31, no. 4 (2021): e12956. 34196989 10.1111/bpa.12956 PMC8412090

[243] C. Chung , X. Yang , T. Bae , et al., “Comprehensive Multi‐omic Profiling of Somatic Mutations in Malformations of Cortical Development, ” Nature Genetics 55, no. 2 (2023): 209‐220. 36635388 10.1038/s41588-022-01276-9 PMC9961399

[244] Y. Wang , Y. Wang , L. Guo , et al., “Spatial Transcriptomics in Focal Cortical Dysplasia Type IIb, ” Acta Neuropathol Commun 12, no. 1 (2024): 185. 39614299 10.1186/s40478-024-01897-7 PMC11607982

[245] S. Baldassari , E. Klingler , L. G. Teijeiro , et al., “Single‐cell Genotyping and Transcriptomic Profiling of Mosaic Focal Cortical Dysplasia, ” Nature Neuroscience 28, no. 5 (2025): 964‐972. 40307383 10.1038/s41593-025-01936-z PMC12081288

[246] B. Puhahn‐Schmeiser , K. Leicht , F. Gessler , T. M. Freiman , “Aberrant Hippocampal Mossy Fibers in Temporal Lobe Epilepsy Target Excitatory and Inhibitory Neurons, ” Epilepsia 62, no. 10 (2021): 2539‐2550. 34453315 10.1111/epi.17035

[247] Q. Liu , C. Shen , Y. Dai , et al., “Single‐cell, Single‐nucleus and Xenium‐based Spatial Transcriptomics Analyses Reveal Inflammatory Activation and Altered Cell Interactions in the Hippocampus in Mice With Temporal Lobe Epilepsy, ” Biomarker Research 12, no. 1 (2024): 103. 39272194 10.1186/s40364-024-00636-3 PMC11396644

[248] Q. Ge , J. Yang , F. Huang , et al., “Multimodal Single‐cell Analyses Reveal Molecular Markers of Neuronal Senescence in human Drug‐resistant Epilepsy, ” Journal of Clinical Investigation 135, no. 5 (2025): e188942. 40026248 10.1172/JCI188942 PMC11870744

[249] Y. Wang , M. Eddison , G. Fleishman , et al., “EASI‐FISH for Thick Tissue Defines Lateral Hypothalamus Spatio‐molecular Organization, ” Cell 184, no. 26 (2021): 6361‐6377. e24. 34875226 10.1016/j.cell.2021.11.024

[250] Y. Ma , K. Zheng , C. Zhao , et al., “Microglia LILRB4 Upregulation Reduces Brain Damage After Acute Ischemic Stroke by Limiting CD8+ T Cell Recruitment, ” J Neuroinflammation 21, no. 1 (2024): 214. 39217343 10.1186/s12974-024-03206-4 PMC11366150

[251] N. Yu , Y. Zhao , P. Wang , F. Zhang , C. Wen , S. Wang , “Changes in Border‐associated Macrophages After Stroke: Single‐cell Sequencing Analysis, ” Neural Regeneration Research 21, no. 1 (2025): 346‐356. Published online January 29, 2025. 39927762 10.4103/NRR.NRR-D-24-01092 PMC12094533

[252] T. Liu , M. Bai , M. Liu , et al., “Novel Synergistic Mechanism of 11‐keto‐β‐boswellic Acid and Z‐Guggulsterone on Ischemic Stroke Revealed by Single‐cell Transcriptomics, ” Pharmacological Research 193 (2023): 106803. 37230158 10.1016/j.phrs.2023.106803

[253] Y. Zhao , Q. Li , J. Niu , et al., “Neutrophil Membrane‐Camouflaged Polyprodrug Nanomedicine for Inflammation Suppression in Ischemic Stroke Therapy, ” Advanced Materials 36, no. 21 (2024): e2311803. 38519052 10.1002/adma.202311803

[254] W. Cai , M. Hu , C. Li , et al., “FOXP3+ macrophage Represses Acute Ischemic Stroke‐induced Neural Inflammation, ” Autophagy 19, no. 4 (2023): 1144‐1163. 36170234 10.1080/15548627.2022.2116833 PMC10012925

[255] C. Liu , H. Sui , Z. Li , et al., “THBS1 in Macrophage‐derived Exosomes Exacerbates Cerebral Ischemia–reperfusion Injury by Inducing Ferroptosis in Endothelial Cells, ” J Neuroinflammation 22, no. 1 (2025): 48. 39994679 10.1186/s12974-025-03382-x PMC11854006

[256] J. E. Kim , R. P. Lee , E. Yazigi , et al., “Soluble PD‐L1 Reprograms Blood Monocytes to Prevent Cerebral Edema and Facilitate Recovery After Ischemic Stroke, ” Brain, Behavior, and Immunity 116 (2024): 160‐174. 38070624 10.1016/j.bbi.2023.12.007 PMC11220828

[257] A. Nakamura , S. Sakai , Y. Taketomi , et al., “PLA2G2E‐mediated Lipid Metabolism Triggers Brain‐autonomous Neural Repair After Ischemic Stroke, ” Neuron 111, no. 19 (2023): 2995‐3010. e9. 37490917 10.1016/j.neuron.2023.06.024

[258] Z. Chen , X. Wang , H. Wu , et al., “X‐box Binding Protein 1 as a Key Modulator in “Healing Endothelial Cells”, a Novel EC Phenotype Promoting Angiogenesis After MCAO, ” Cellular & Molecular Biology Letters 27, no. 1 (2022): 97. 36348288 10.1186/s11658-022-00399-5 PMC9644469

[259] A. Loan , N. Awaja , M. Lui , et al., “Single‐cell Profiling of Brain Pericyte Heterogeneity Following Ischemic Stroke Unveils Distinct Pericyte Subtype‐targeted Neural Reprogramming Potential and Its Underlying Mechanisms, ” Theranostics 14, no. 16 (2024): 6110‐6137. 39431007 10.7150/thno.97165 PMC11488099

[260] X. Wang , A. Zhang , Q. Yu , et al., “Single‐Cell RNA Sequencing and Spatial Transcriptomics Reveal Pathogenesis of Meningeal Lymphatic Dysfunction After Experimental Subarachnoid Hemorrhage, ” Adv Sci (Weinh) 10, no. 21 (2023): e2301428. 37211686 10.1002/advs.202301428 PMC10375135

[261] J. Zheng , H. Wu , X. Wang , et al., “Temporal Dynamics of Microglia‐astrocyte Interaction in Neuroprotective Glial Scar Formation After Intracerebral Hemorrhage, ” Journal of Pharmaceutical Analysis 13, no. 8 (2023): 862‐879. 37719195 10.1016/j.jpha.2023.02.007 PMC10499589

[262] X. S. Li , W. Liu , G. Jiang , et al., “Celastrol Ameliorates Neuronal Mitochondrial Dysfunction Induced by Intracerebral Hemorrhage via Targeting cAMP‐Activated Exchange Protein‐1, ” Adv Sci (Weinh) 11, no. 19 (2024): e2307556. 38482725 10.1002/advs.202307556 PMC11109624

[263] Y. Niu , X. Chen , Y. Zhang , Y. Ge , J. Gao , T. Huang , “Decoding Neuronal Genes in Stroke‐induced Pain: Insights From Single‐nucleus Sequencing in Mice, ” BMC Neurology [Electronic Resource] 24, no. 1 (2024): 459. 39581982 10.1186/s12883-024-03965-w PMC11587673

[264] C. Wang , M. Xiong , M. Gratuze , et al., “Selective Removal of Astrocytic APOE4 Strongly Protects Against Tau‐mediated Neurodegeneration and Decreases Synaptic Phagocytosis by Microglia, ” Neuron 109, no. 10 (2021): 1657‐1674. e7. 33831349 10.1016/j.neuron.2021.03.024 PMC8141024

[265] N. Koutsodendris , J. Blumenfeld , A. Agrawal , et al., “Neuronal APOE4 Removal Protects Against Tau‐mediated Gliosis, Neurodegeneration and Myelin Deficits, ” Nat Aging 3, no. 3 (2023): 275‐296. 37118426 10.1038/s43587-023-00368-3 PMC10154214

[266] A. Rao , N. Chen , M. J. Kim , et al., “Microglia Depletion Reduces human Neuronal APOE4‐related Pathologies in a Chimeric Alzheimer's Disease Model, ” Cell Stem Cell 32, no. 1 (2025): 86‐104. e7. 39500314 10.1016/j.stem.2024.10.005 PMC11701721

[267] C. S. McAlpine , J. Park , A. Griciuc , et al., “Astrocytic Interleukin‐3 Programs Microglia and Limits Alzheimer's Disease, ” Nature 595, no. 7869 (2021): 701‐706. 34262178 10.1038/s41586-021-03734-6 PMC8934148

[268] B. van Lengerich , L. Zhan , D. Xia , et al., “A TREM2‐activating Antibody With a Blood‐brain Barrier Transport Vehicle Enhances Microglial Metabolism in Alzheimer's disease Models, ” Nature Neuroscience 26, no. 3 (2023): 416‐429. 36635496 10.1038/s41593-022-01240-0 PMC9991924

[269] L. Van Olst , B. Simonton , A. J. Edwards , et al., “Microglial Mechanisms Drive Amyloid‐β Clearance in Immunized Patients With Alzheimer's Disease, ” Nature Medicine 31, no. 5 (2025): 1604‐1616. Published online March 6, 2025. 10.1038/s41591-025-03574-1 PMC12092304 40050704

[270] F. A. Sayed , L. Kodama , L. Fan , et al., “AD‐linked R47H‐TREM2 Mutation Induces Disease‐enhancing Microglial States via AKT Hyperactivation, ” Science Translational Medicine 13, no. 622 (2021): eabe3947. 34851693 10.1126/scitranslmed.abe3947 PMC9345574

[271] Ö. İş , X. Wang , J. S. Reddy , et al., “Gliovascular Transcriptional Perturbations in Alzheimer's Disease Reveal Molecular Mechanisms of Blood Brain Barrier Dysfunction, ” Nature Communications 15, no. 1 (2024): 4758. 10.1038/s41467-024-48926-6 PMC11190273 38902234

[272] S. De Schepper , J. Z. Ge , G. Crowley , et al., “Perivascular Cells Induce Microglial Phagocytic States and Synaptic Engulfment via SPP1 in Mouse Models of Alzheimer's Disease, ” Nature Neuroscience 26, no. 3 (2023): 406‐415. 36747024 10.1038/s41593-023-01257-z PMC9991912

[273] W. Qu , M. Lam , J. J. McInvale , et al., “Xenografted human iPSC‐derived Neurons With the Familial Alzheimer's disease APPV717I Mutation Reveal Dysregulated Transcriptome Signatures Linked to Synaptic Function and Implicate LINGO2 as a Disease Signaling Mediator, ” Acta Neuropathologica 147, no. 1 (2024): 107. 38918213 10.1007/s00401-024-02755-5 PMC11199265

[274] M. Jorfi , J. Park , C. K. Hall , et al., “Infiltrating CD8+ T Cells Exacerbate Alzheimer's Disease Pathology in a 3D human Neuroimmune Axis Model, ” Nature Neuroscience 26, no. 9 (2023): 1489‐1504. 37620442 10.1038/s41593-023-01415-3 PMC11184920

[275] P. Xu , H. He , Q. Gao , et al., “Human Midbrain Dopaminergic Neuronal Differentiation Markers Predict Cell Therapy Outcomes in a Parkinson's disease Model, ” Journal of Clinical Investigation 132, no. 14 (2022): e156768. 35700056 10.1172/JCI156768 PMC9282930

[276] K. Nishimura , S. Yang , K. W. Lee , et al., “Single‐cell Transcriptomics Reveals Correct Developmental Dynamics and High‐quality Midbrain Cell Types by Improved hESC Differentiation, ” Stem Cell Reports 18, no. 1 (2022): 337‐353. 36400027 10.1016/j.stemcr.2022.10.016 PMC9860082

[277] J. Giehrl‐Schwab , F. Giesert , B. Rauser , et al., “Parkinson's Disease Motor Symptoms Rescue by CRISPRa‐reprogramming Astrocytes Into GABAergic Neurons, ” EMBO Molecular Medicine 14, no. 5 (2022): e14797. 35373464 10.15252/emmm.202114797 PMC9081909

[278] Y. Zhuo , W. S. Li , W. Lu , et al., “TGF‐β1 Mediates Hypoxia‐preconditioned Olfactory Mucosa Mesenchymal Stem Cells Improved Neural Functional Recovery in Parkinson's disease Models and Patients, ” Mil Med Res 11 (2024): 48. 39034405 10.1186/s40779-024-00550-7 PMC11265117

[279] W. Kong , Y. Liu , P. Ai , et al., “Genetically Modified E. Coli Secreting Melanin (E.melanin) Activates the Astrocytic PSAP‐GPR37L1 Pathway and Mitigates the Pathogenesis of Parkinson's Disease, ” J Nanobiotechnology 22 (2024): 690. 39523310 10.1186/s12951-024-02955-x PMC11552183

[280] R. X. Zhu , Y. H. Chen , X. Xia , et al., “Formation of CSE‐YAP Complex Drives FOXD3‐mediated Transition of Neurotoxic Astrocytes in Parkinson's Disease, ” Pharmacological Research 210 (2024): 107507. 39547464 10.1016/j.phrs.2024.107507

[281] H. Hong , Y. Wang , M. Menard , et al., “Suppression of the JAK/STAT Pathway Inhibits Neuroinflammation in the Line 61‐PFF Mouse Model of Parkinson's Disease, ” J Neuroinflammation 21 (2024): 216. 39218899 10.1186/s12974-024-03210-8 PMC11368013

[282] L. H. Geraldo , Y. Xu , L. Jacob , et al., “SLIT2/ROBO Signaling in Tumor‐associated Microglia and Macrophages Drives Glioblastoma Immunosuppression and Vascular Dysmorphia, ” The Journal of Clinical Investigation 131, no. 16 (2021): e141083. 34181595 10.1172/JCI141083 PMC8363292

[283] J. Li , M. M. Kaneda , J. Ma , et al., “PI3Kγ inhibition Suppresses Microglia/TAM Accumulation in Glioblastoma Microenvironment to Promote Exceptional Temozolomide Response, ” Proceedings of the National Academy of Sciences of the United States of America 118, no. 16 (2021): e2009290118. 33846242 10.1073/pnas.2009290118 PMC8072253

[284] P. Chen , W. H. Hsu , A. Chang , et al., “Circadian Regulator CLOCK Recruits Immune Suppressive Microglia Into the GBM Tumor Microenvironment, ” Cancer Discovery 10, no. 3 (2020): 371. 31919052 10.1158/2159-8290.CD-19-0400 PMC7058515

[285] W. Xuan , W. H. Hsu , F. Khan , et al., “Circadian Regulator CLOCK Drives Immunosuppression in Glioblastoma, ” Cancer immunology research 10, no. 6 (2022): 770‐784. 35413115 10.1158/2326-6066.CIR-21-0559 PMC9177794

[286] S. Goswami , T. Walle , A. E. Cornish , et al., “Immune Profiling of human Tumors Identifies CD73 as a Combinatorial Target in Glioblastoma, ” Nature Medicine 26, no. 1 (2020): 39‐46. 10.1038/s41591-019-0694-x PMC7182038 31873309

[287] V. M. Ravi , N. Neidert , P. Will , et al., “T‐cell Dysfunction in the Glioblastoma Microenvironment Is Mediated by Myeloid Cells Releasing Interleukin‐10, ” Nature Communications 13 (2022): 925. 10.1038/s41467-022-28523-1 PMC8854421 35177622

[288] E. A. Winkler , C. N. Kim , J. M. Ross , et al., “A Single‐cell Atlas of the Normal and Malformed human Brain Vasculature, ” Science 375, no. 6584 (2022): eabi7377. 35084939 10.1126/science.abi7377 PMC8995178

[289] X. Hong , Y. Jian , S. Ding , et al., “Kir4.1 channel Activation in NG2 Glia Contributes to Remyelination in Ischemic Stroke, ” Ebiomedicine 87 (2023): 104406. 36527899 10.1016/j.ebiom.2022.104406 PMC9791134

[290] Y. Liao , J. Wang , C. Guo , et al., “Combination of Systems Pharmacology and Experimental Evaluation to Explore the Mechanism of Synergistic Action of Frankincense‐Myrrh in the Treatment of Cerebrovascular Diseases, ” Frontiers in Pharmacology 12 (2022): 796224. 35082676 10.3389/fphar.2021.796224 PMC8784887

[291] Z. Zhu , Y. Fu , D. Tian , et al., “Combination of the Immune Modulator Fingolimod with Alteplase in Acute Ischemic Stroke: A Pilot Trial, ” Circulation 132, no. 12 (2015): 1104‐1112. 26202811 10.1161/CIRCULATIONAHA.115.016371 PMC4580515

[292] W. Cai , X. Dai , J. Chen , et al., “STAT6/Arg1 promotes Microglia/Macrophage Efferocytosis and Inflammation Resolution in Stroke Mice, ” JCI Insight 4, no. 20 (2019): e131355. 31619589 10.1172/jci.insight.131355 PMC6824303

[293] R. Liu , P. Song , X. Gu , et al., “Comprehensive Landscape of Immune Infiltration and Aberrant Pathway Activation in Ischemic Stroke, ” Frontiers in immunology 12 (2021): 766724. 35140708 10.3389/fimmu.2021.766724 PMC8818702

[294] H. Sato , Y. Taketomi , A. Ushida , et al., “The Adipocyte‐inducible Secreted Phospholipases PLA2G5 and PLA2G2E Play Distinct Roles in Obesity, ” Cell metabolism 20, no. 1 (2014): 119‐132. 24910243 10.1016/j.cmet.2014.05.002 PMC4079757

[295] C. Ma , G. CB , H. S. Rp , et al., “Citrullination Regulates Pluripotency and Histone H1 Binding to Chromatin, ” Nature 507, no. 7490 (2014): 104‐108. 24463520 10.1038/nature12942 PMC4843970

[296] S. C. Stadler , C. T. Vincent , V. D. Fedorov , et al., “Dysregulation of PAD4‐mediated Citrullination of Nuclear GSK3β Activates TGF‐β Signaling and Induces Epithelial‐to‐mesenchymal Transition in Breast Cancer Cells, ” PNAS 110, no. 29 (2013): 11851‐11856. 23818587 10.1073/pnas.1308362110 PMC3718105

[297] L. Shi , Z. Sun , W. Su , et al., “Treg Cell‐derived Osteopontin Promotes Microglia‐mediated White Matter Repair After Ischemic Stroke, ” Immunity 54, no. 7 (2021): 1527‐1542. e8. 34015256 10.1016/j.immuni.2021.04.022 PMC8282725

[298] Q. Duan , L. Ni , P. Wang , et al., “Deregulation of XBP1 Expression Contributes to Myocardial Vascular Endothelial Growth Factor‐A Expression and Angiogenesis During Cardiac Hypertrophy in Vivo, ” Aging Cell 15, no. 4 (2016): 625‐633. 27133203 10.1111/acel.12460 PMC4933664

[299] J. H. Ahn , H. Cho , J. H. Kim , et al., “Meningeal Lymphatic Vessels at the Skull Base Drain Cerebrospinal Fluid, ” Nature 572, no. 7767 (2019): 62‐66. 31341278 10.1038/s41586-019-1419-5

[300] A. Louveau , I. Smirnov , T. J. Keyes , et al., “Structural and Functional Features of central Nervous System Lymphatic Vessels, ” Nature 523, no. 7560 (2015): 337‐341. 26030524 10.1038/nature14432 PMC4506234

[301] J. Chen , L. Wang , H. Xu , et al., “Meningeal Lymphatics Clear Erythrocytes That Arise From Subarachnoid Hemorrhage, ” Nature Communications 11, no. 1 (2020): 3159. 10.1038/s41467-020-16851-z PMC7308412 32572022

[302] G. Oliver , J. Kipnis , G. J. Randolph , N. L. Harvey , “The Lymphatic Vasculature in the 21st Century: Novel Functional Roles in Homeostasis and Disease, ” Cell 182, no. 2 (2020): 270‐296. 32707093 10.1016/j.cell.2020.06.039 PMC7392116

[303] D. Pham , X. Tan , B. Balderson , et al., “Robust Mapping of Spatiotemporal Trajectories and Cell–cell Interactions in Healthy and Diseased Tissues, ” Nature Communications 14, no. 1 (2023): 7739. 10.1038/s41467-023-43120-6 PMC10676408 38007580

[304] Z. Hong , J. Cao , D. Liu , et al., “Celastrol Targeting Nedd4 Reduces Nrf2‐mediated Oxidative Stress in Astrocytes After Ischemic Stroke, ” Journal of Pharmaceutical Analysis 13, no. 2 (2023): 156‐169. 36908855 10.1016/j.jpha.2022.12.002 PMC9999302

[305] H. Xu , H. Zhao , C. Ding , et al., “Celastrol Suppresses Colorectal Cancer via Covalent Targeting Peroxiredoxin 1, ” Signal Transduct Target Ther 8, no. 1 (2023): 51. 36732502 10.1038/s41392-022-01231-4 PMC9895061

[306] C. Y. Yan , S. H. Ouyang , X. Wang , et al., “Celastrol Ameliorates Propionibacterium Acnes/LPS‐induced Liver Damage and MSU‐induced Gouty Arthritis via Inhibiting K63 Deubiquitination of NLRP3, ” Phytomedicine 80 (2021): 153398. 33130474 10.1016/j.phymed.2020.153398

[307] M. Jiang , X. Liu , D. Zhang , et al., “Celastrol Treatment Protects Against Acute Ischemic Stroke‐induced Brain Injury by Promoting an IL‐33/ST2 Axis‐mediated Microglia/Macrophage M2 Polarization, ” J Neuroinflammation 15, no. 1 (2018): 78. 29540209 10.1186/s12974-018-1124-6 PMC5853059

[308] W. Zhang , J. Wang , C. Yang , “Celastrol, a TFEB (transcription factor EB) Agonist, Is a Promising Drug Candidate for Alzheimer Disease, ” Autophagy 18, no. 7 (2022): 1740‐1742. 35253615 10.1080/15548627.2022.2046437 PMC9298436

[309] E. Parnell , T. M. Palmer , S. J. Yarwood , “The Future of EPAC‐targeted Therapies: Agonism versus Antagonism, ” Trends in Pharmacological Sciences 36, no. 4 (2015): 203‐214. 25744542 10.1016/j.tips.2015.02.003 PMC4392396

[310] T. K. Ulland , W. M. Song , S. C. C. Huang , et al., “TREM2 Maintains Microglial Metabolic Fitness in Alzheimer's Disease, ” Cell 170, no. 4 (2017): 649‐663. e13. 28802038 10.1016/j.cell.2017.07.023 PMC5573224

[311] E. Morenas‐Rodríguez , Y. Li , B. Nuscher , et al., “Soluble TREM2 in CSF and Its Association With Other Biomarkers and Cognition in Autosomal‐dominant Alzheimer's Disease: A Longitudinal Observational Study, ” Lancet Neurology 21, no. 4 (2022): 329‐341. 35305339 10.1016/S1474-4422(22)00027-8 PMC8926925

[312] Y. Zhou , W. M. Song , P. Andhey , et al., “Human and Mouse Single‐nucleus Transcriptomics Reveal TREM2‐dependent and ‐independent Cellular Responses in Alzheimer's Disease, ” The Journal of Immunology 204, _Supplement no. 1 (2020): 64.2‐64.2.

[313] D. Wang , F. Chen , Z. Han , Z. Yin , X. Ge , P. Lei , “Relationship between Amyloid‐β Deposition and Blood‐Brain Barrier Dysfunction in Alzheimer's Disease, ” Front Cell Neurosci 15 (2021): 695479. 34349624 10.3389/fncel.2021.695479 PMC8326917

[314] R. Browaeys , W. Saelens , Y. Saeys , “NicheNet: Modeling Intercellular Communication by Linking Ligands to Target Genes, ” Nature Methods 17, no. 2 (2020): 159‐162. 31819264 10.1038/s41592-019-0667-5

[315] D. Gate , N. Saligrama , O. Leventhal , et al., “Clonally Expanded CD8 T Cells Patrol the Cerebrospinal Fluid in Alzheimer's Disease, ” Nature 577, no. 7790 (2020): 399‐404. 31915375 10.1038/s41586-019-1895-7 PMC7445078

[316] M. S. Unger , E. Li , L. Scharnagl , et al., “CD8(+) T‐cells Infiltrate Alzheimer's disease Brains and Regulate Neuronal‐ and Synapse‐related Gene Expression in APP‐PS1 Transgenic Mice, ” Brain, Behavior, and Immunity 89 (2020): 67‐86. 32479993 10.1016/j.bbi.2020.05.070

[317] M. Merlini , T. Kirabali , L. Kulic , R. M. Nitsch , M. T. Ferretti , “Extravascular CD3+ T Cells in Brains of Alzheimer Disease Patients Correlate With Tau but Not With Amyloid Pathology: An Immunohistochemical Study, ” Neurodegener Dis 18, no. 1 (2018): 49‐56. 29402847 10.1159/000486200

[318] T. Togo , H. Akiyama , E. Iseki , et al., “Occurrence of T Cells in the Brain of Alzheimer's Disease and Other Neurological Diseases, ” Journal of Neuroimmunology 124, no. 1‐2 (2002): 83‐92. 11958825 10.1016/s0165-5728(01)00496-9

[319] F. Nilsson , P. Storm , E. Sozzi , et al., “Single‐Cell Profiling of Coding and Noncoding Genes in Human Dopamine Neuron Differentiation, ” Cells 10, no. 1 (2021): 137. 33445654 10.3390/cells10010137 PMC7827700

[320] M. Birtele , P. Storm , Y. Sharma , et al., “Single‐cell Transcriptional and Functional Analysis of Dopaminergic Neurons in Organoid‐Like Cultures Derived From human Fetal Midbrain, ” Development (Cambridge, England) 149, no. 23 (2022): dev200504. 36305490 10.1242/dev.200504 PMC10114107

[321] X. Zeng , W. Geng , J. Jia , Z. Wang , “Advances in Stem Cells Transplantation for the Therapy of Parkinson's Disease, ” Curr Stem Cell Res Ther 16, no. 8 (2021): 958‐969. 33687901 10.2174/1574888X16666210309153949

[322] Y. Chen , J. Shen , K. Ke , X. Gu , “Clinical Potential and Current Progress of Mesenchymal Stem Cells for Parkinson's disease: A Systematic Review, ” Neurol Sci 41, no. 5 (2020): 1051‐1061. 31919699 10.1007/s10072-020-04240-9

[323] Y. Zhuo , W. Chen , W. Li , “Ischemic‐hypoxic Preconditioning Enhances the Mitochondrial Function Recovery of Transplanted Olfactory Mucosa Mesenchymal Stem Cells via miR‐181a Signaling in Ischemic Stroke, ” Aging (Albany NY) 13, no. 8 (2021): 11234‐11256. 33820869 10.18632/aging.202807 PMC8109091

[324] D. Kempuraj , R. Thangavel , P. Natteru , et al., “Neuroinflammation Induces Neurodegeneration, ” J Neurol Neurosurg Spine 1, no. 1 (2016): 1003. 28127589 PMC5260818

[325] K. Aslan , V. Turco , J. Blobner , et al., “Heterogeneity of Response to Immune Checkpoint Blockade in Hypermutated Experimental Gliomas, ” Nature Communications 11 (2020): 931. 10.1038/s41467-020-14642-0 PMC7028933 32071302

[326] J. Zhao , A. X. Chen , R. D. Gartrell , et al., “Immune and Genomic Correlates of Response to anti‐PD‐1 Immunotherapy in Glioblastoma, ” Nature Medicine 25, no. 3 (2019): 462. 10.1038/s41591-019-0349-y PMC6810613 30742119

[327] I. S. C. Verploegh , A. Conidi , R. W. W. Brouwer , et al., “Comparative Single‐cell RNA‐sequencing Profiling of BMP4‐treated Primary Glioma Cultures Reveals Therapeutic Markers, ” Neuro‐oncol 24, no. 12 (2022): 2133‐2145. 35639831 10.1093/neuonc/noac143 PMC9713526

[328] A. R. Pombo Antunes , I. Scheyltjens , F. Lodi , et al., “Single‐cell Profiling of Myeloid Cells in Glioblastoma Across Species and Disease Stage Reveals Macrophage Competition and Specialization, ” Nature Neuroscience 24, no. 4 (2021): 595‐610. 33782623 10.1038/s41593-020-00789-y

[329] S. Coy , S. Wang , S. A. Stopka , et al., “Single Cell Spatial Analysis Reveals the Topology of Immunomodulatory Purinergic Signaling in Glioblastoma, ” Nature Communications 13, no. 1 (2022): 4814. 10.1038/s41467-022-32430-w PMC9381513 35973991

[330] L. Antonioli , S. V. Novitskiy , K. F. Sachsenmeier , M. Fornai , C. Blandizzi , G. Haskó , “Switching off CD73: A Way to Boost the Activity of Conventional and Targeted Antineoplastic Therapies, ” Drug Discovery Today 22, no. 11 (2017): 1686‐1696. 28676406 10.1016/j.drudis.2017.06.005

[331] P. I , M. Ha , G. P. M , “Blocking Antibodies Targeting the CD39/CD73 Immunosuppressive Pathway Unleash Immune Responses in Combination Cancer Therapies, ” Cell Reports 27, no. 8 (2019): 2411‐2425. 31116985 10.1016/j.celrep.2019.04.091

[332] L. Song , W. Chen , J. Hou , M. Guo , J. Yang , “Spatially Resolved Mapping of Cells Associated With human Complex Traits, ” Nature 641, no. 8064 (2025): 932‐941. 40108460 10.1038/s41586-025-08757-x PMC12095064

[333] K. Lebrigand , V. Magnone , P. Barbry , R. Waldmann , “High Throughput Error Corrected Nanopore Single Cell Transcriptome Sequencing, ” Nature Communications 11, no. 1 (2020): 4025. 10.1038/s41467-020-17800-6 PMC7423900 32788667

[334] M. Hagemann‐Jensen , C. Ziegenhain , P. Chen , et al., “Single‐cell RNA Counting at Allele and Isoform Resolution Using Smart‐seq3, ” Nature Biotechnology 38, no. 6 (2020): 708‐714. 10.1038/s41587-020-0497-0 32518404

[335] I. Gupta , P. G. Collier , B. Haase , et al., “Single‐cell Isoform RNA Sequencing Characterizes Isoforms in Thousands of Cerebellar Cells, ” Nature Biotechnology (2018). Published online October 15, 2018. 10.1038/nbt.4259 30320766

[336] K. Lebrigand , J. Bergenstråhle , K. Thrane , et al., “The Spatial Landscape of Gene Expression Isoforms in Tissue Sections, ” Nucleic Acids Research 51, no. 8 (2023): e47‐e47. 36928528 10.1093/nar/gkad169 PMC10164556

[337] L. Tarhan , J. Bistline , J. Chang , B. Galloway , E. Hanna , E. Weitz , “Single Cell Portal: An Interactive Home for Single‐cell Genomics Data, ” BioRxiv 17 (2023). Published online July.

[338] A. Regev , S. A. Teichmann , E. S. Lander , et al., “The Human Cell Atlas, ” Elife 6 (2017): e27041. 29206104 10.7554/eLife.27041 PMC5762154

[339] M. L. Speir , A. Bhaduri , N. S. Markov , et al., “UCSC Cell Browser: Visualize Your Single‐cell Data, ” Bioinformatics 37, no. 23 (2021): 4578‐4580. 34244710 10.1093/bioinformatics/btab503 PMC8652023

[340] R. C. Jones , J. Karkanias , M. A. Krasnow , et al., “The Tabula Sapiens: A Multiple‐organ, Single‐cell Transcriptomic Atlas of Humans, ” Science 376, no. 6594 (2022): eabl4896. 35549404 10.1126/science.abl4896 PMC9812260

[341] HuBMAP Consortium . The human Body at Cellular Resolution: The NIH Human Biomolecular Atlas Program. Nature 2019;574(7777):187‐192. 31597973 10.1038/s41586-019-1629-x PMC6800388

